# Targeted Inactivation of an *α/β* Hydrolase Gene Enables Discovery of Heterodimeric Nenestatins

**DOI:** 10.3390/md24030103

**Published:** 2026-03-08

**Authors:** Wenzheng Wei, Xiaodong Jiang, Yiguang Zhu, Wenjun Zhang, Chunfang Yang, Qingbo Zhang, Changsheng Zhang

**Affiliations:** 1 State Key Laboratory of Tropical Oceanography, Guangdong Key Laboratory of Marine Materia Medica, South China Sea Institute of Oceanology, Chinese Academy of Sciences, 164 West Xingang Road, Guangzhou 510301, China; weiwenzheng23@mails.ucas.ac.cn (W.W.); ygzhu@scsio.ac.cn (Y.Z.); wzhang@scsio.ac.cn (W.Z.); yangchunfang@scsio.ac.cn (C.Y.); 2 University of Chinese Academy of Sciences, Beijing 100049, China; 3 Guangxi Key Laboratory of Marine Drugs, University Engineering Research Center of High-Efficient Utilization of Marine Traditional Chinese Medicine Resources, Institute of Marine Drugs, Guangxi University of Chinese Medicine, Nanning 530200, China; jxd374487986@163.com

**Keywords:** marine natural products, angucyclines, *α/β* hydrolase, dimerization

## Abstract

Nenestatins (NENs) belong to benzo[*b*]fluorene-containing atypical angucyclines, a structurally diverse class of microbial natural products. Bioinformatic analysis of the NEN biosynthetic gene cluster (*nes* BGC) from the deep-sea sediment-derived *Micromonospora echinospora* SCSIO 04089 implicated Nes5 as an *α/β* hydrolase. The targeted inactivation of the *nes5* gene led to the accumulation of five new analogs, NENs E–I (**1**–**5**), together with the known monomer homo-dehydrorabelomycin E (**6**). Their structures were elucidated by comprehensive spectroscopic analysis and electronic circular dichroism calculations. Notably, both NEN A and NEN B were absent in the Δ*nes5* mutant, indicating that Nes5 is essential for their biosynthesis; however, the exact function of Nes5 requires further exploration.

## 1. Introduction

Benzo[*b*]fluorene-containing atypical angucyclines are a structurally diverse family of microbial natural products with significant biological activities [1,2]. Their biosynthesis typically originates from type II polyketide synthases (PKSs) [2,3,4]. The resulting tetracyclic precursors undergo a B-ring cleavage and subsequent rearrangement to form the characteristic benzofluorene core, which is further diversified by tailoring modifications such as oxidation, glycosylation, and dimerization [2,3,4,5]. Among all post-tailoring modifications, dimerization is especially critical for enhancing biological activity. For instance, lomaiviticin A exhibits nanomolar to picomolar cytotoxicity against a range of human cancer cell lines, with potency two orders of magnitude greater than that of its monomers [6,7,8]. Similarly, the asymmetric heterodimer difluostatin A shows superior antibacterial activity compared to its constituent monomers [9]. These cases highlight that dimerization is critical for the biological activities of this class of compounds.

**Figure 1:**
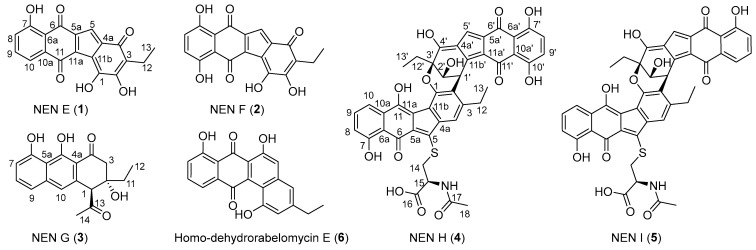
The structures of new NENs, **1**–**5**, and homo-dehydrorabelomycin E (**6**).

Nenestatins (NENs) are a family of benzo[*b*]fluorene-containing atypical angucyclines isolated from the deep-sea sediment-derived actinomycete *Micromonospora echinospora* SCSIO 04089, with NEN B featuring an asymmetric dimeric architecture [10,11]. Although a previous study suggested that the NmrA-family protein Nes18 might be responsible for the C-2–C-1′ dimerization to generate NEN B, this enzymatic activity has yet to be biochemically confirmed in vitro [11]. Recently, in our ongoing effort to investigate the biosynthetic pathway of NENs, an *α/β* hydrolase encoding gene, *nes5*, was disrupted, which led to the accumulation of a series of novel NEN congeners (Figure S1). Herein, we report the targeted inactivation of *nes5* in *M. echinospora* SCSIO 04089 and the isolation, structure elucidation, and bioactivity evaluation of five new nenestatin analogs, NENs E–I (**1**–**5**), together with the known analog homo-dehydrorabelomycin E (**6**) [12], from the Δ*nes5* mutant (Figure 1). Notably, NENs H and I (**4** and **5**) represent two novel dimers.

## 2. Results and Discussion

The *nes5* gene was inactivated in *M. echinospora* SCSIO 04089 to generate the Δ*nes5* mutant. Fermentation of this mutant in a medium supplemented with macroporous resin HP20 yielded six nenestatin analogs (**1**–**6**), including three new monomers, NENs E–G (**1**–**3**), two new dimers, NENs H and I (**4** and **5**), and the known compound homo-dehydrorabelomycin E (**6**) [12] (Figure 1).

**Figure 2:**
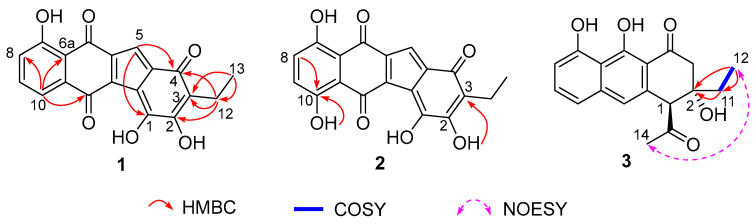
Key 2D NMR correlations of compounds **1**–**3**.

NEN E (**1**) was obtained as a red amorphous powder. Its molecular formula was established as C_19_H_12_O_6_ by HRMS (*m/z*: 335.0567 [M − H]^−^, calcd for C_19_H_11_O_6_, 335.0561), indicating fourteen degrees of unsaturation. Analysis of the NMR data of **1** (Table 1 and Figure S2) revealed signals for an ABC three-spin system (*δ*_C_/*δ*_H_ 134.6/7.53, 121.5/7.05, and 118.0/7.50), a sp^2^-hybridized methine (*δ*_C_/*δ*_H_ 115.0/6.88), a quartet methylene (*δ*_C_/*δ*_H_ 15.8/2.32), a triplet methyl group (*δ*_C_/*δ*_H_ 13.1/0.97), and thirteen sp^2^-hybridized non-protonated carbons. Comparative NMR analysis showed that **1** is highly similar to nenestatin C [11]. The signals for an AB-spin system (*δ*_C_/*δ*_H_ 121.7/7.22, C-1; *δ*_C_/δ_H_ 137.7/5.94, C-2) and a non-protonated carbon (*δ*_C_ 76.3, C-3) were present in nenestatin C, while absent in **1**. Instead, three sp^2^-hybridized methines (*δ*_C_ 175.1, 156.1, 120.2), two of which are oxygenated, were observed in **1**. The HMBC correlations from H_3_-13 to C-12 (*δ*_C_ 15.8)/C-3 (*δ*_C_ 120.2), from H-5 to C-4, and from H_2_-12 to C-2 (*δ*_C_ 156.1)/C-3/C-4 (*δ*_C_ 183.9) suggested the presence of a double bond between C-2 and C-3 (Figure 2). Additionally, the carbon at *δ*_C_ 175.1 was assigned as C-1 by the ^4^*J* long-distance HMBC correlation from H-5 to C-1. Furthermore, the 10-OH in nenestatin C was absent in **1**. The assignment of **1** was further supported by HMBC correlations from H-10 to C-6a/C-8/C-11 (Figure 2).

NEN F (**2**) was isolated as a red amorphous powder. The molecular formula of **2** was established as C_19_H_12_O_7_ through HRMS (*m/z*: 351.0515 [M − H]^−^, calcd for C_19_H_11_O_7_, 351.0510), indicating the incorporation of an additional oxygen atom compared to **1**. Analysis of the NMR data of **2** (Table 1 and Figure S3) revealed that the structures of **2** and **1** were highly similar. Different from the olefinic methine CH-10 (*δ*_C_/*δ*_H_ 118.0/7.50) present in **1**, an oxygenated non-protonated C-10 (*δ*_C_ 155.6) was found in **2**. The assignment was confirmed by the HMBC correlations from 10-OH and H-8 to C-10 (Figure 2). Additionally, the exchangeable proton at *δ*_H_ 9.59 was assigned to the 2-OH group based on its HMBC correlation to C-3 (*δ*_C_ 120.1) (Figure 2). Therefore, **2** was determined as a C-10 hydroxylated derivative of **1**.

**Figure 3:**
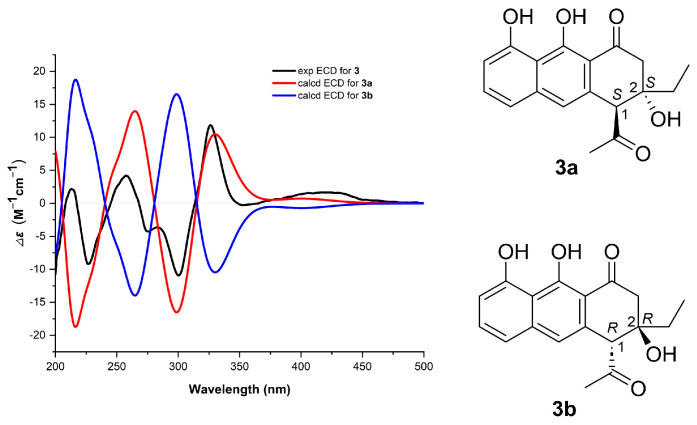
Comparison of the experimental (Exp) ECD of **3** with those calculated (Calcd) for **3a** and **3b**.

NEN G (**3**) was isolated as a brown amorphous powder. Its molecular formula was established to be C_18_H_18_O_5_ through HRMS (*m/z*: 313.1081 [M − H]^−^, calcd for C_18_H_17_O_5_, 313.1081). The NMR data of **3** (Table 1 and Figure S4) were highly similar to those of huanglongmycin H [13]. The only difference was that the methyl group in huanglongmycin H was replaced with an ethyl group (*δ*_C_/*δ*_H_ 32.1/1.51; 1.64, CH_2_-11; 6.80/0.97, CH_3_-12) in **3**. The assignment was supported by the COSY correlate between H_2_-11/H_3_-12, together with HMBC correlations from H_3_-12 to C-2/C-11 and from H_2_-11 to C-2 (Figure 2). The NOESY correlation between H_3_-14 and H_3_-12 indicated a *syn* orientation of the acetyl group at C-1 and the ethyl group at C-2 (Figure 2). Finally, the absolute configuration of **3** was assigned to be 1*S*,2*S*, given that the experimental electronic circular dichroism (ECD) spectrum of **3** matched well with that calculated for the model compound **3a** (1*S*,2*S*) (Figure 3 and Figure S5). The assignment was further corroborated by the closely matched specific rotation values of **3** ${\left([\alpha \right]}_{D}^{25}$ = −21.0°) and huanglongmycin H (${\left[\alpha \right]}_{D}^{25}$ = −21.4°).

**Figure 4:**
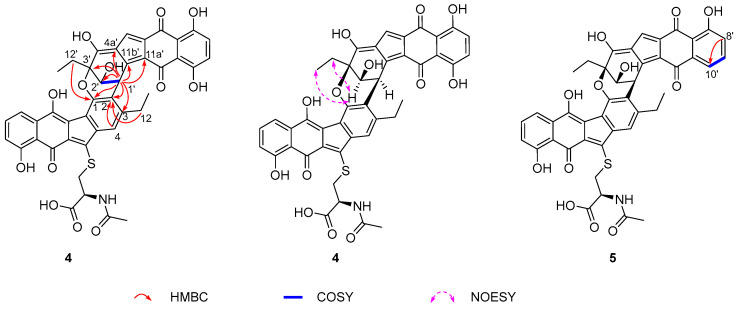
Key 2D NMR correlations of compounds **4** and **5**.

**Figure 5:**
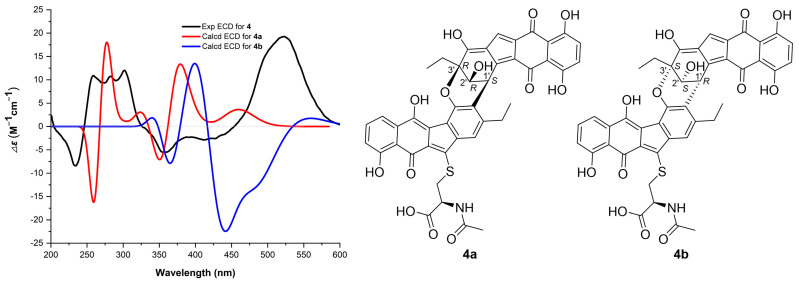
Comparison of the Exp ECD of **4** with those Calcd for **4a** and **4b**.

NEN H (**4**) was isolated as a purple amorphous powder. Its molecular formula was established as C_43_H_33_NO_13_S through HRMS (*m/z*: 802.1607 [M − H]^−^, calcd for C_43_H_32_NO_13_S, 802.1600), corresponding to 28 degrees of unsaturation. Analysis of the ^1^H, ^13^C and 2D NMR data of **4** revealed the presence of two structural units, A and B (Table 2 and Figure S6). The NMR data for the unit A closely resembled those of (-)-homoseongomycin [14]. The key difference was at the C-2 position: while (-)-homoseongomycin has a sp^2^-hybridized methine (*δ*_C_*/δ*_H_ 114.3/6.63, CH-3), the unit A exhibited a non-protonated carbon at *δ*_C_ 126.5 (C-2). The assignment was supported by HMBC correlations from H_2_-12 and H-4 to C-2 in **4** (Figure 4). The unit B displayed ^1^H and ^13^C NMR data consistent with those of that in nenestatin D [11]. The obvious difference was the presence of a sp^3^-hybridized CH-1′ (*δ*_C_*/δ*_H_ 36.1/5.12) in the unit B of **4**, instead of the sp^2^-hybridized and non-protonated C-1′ (*δ*_C_ 141.3) in nenestatin D [11]. The assignment was supported by the COSY correlation between H-1′ and H-2′, and the HMBC correlations from H-1′ to C-2′/C-3′/C-4a′/C-11a′/C-11b′. Moreover, HMBC correlations from H-1′ to C-1, C-2, and C-3 indicated a connection between CH-1′ and C-2 (Figure 4). The proposed structure accounted for only 27 degrees of unsaturation, one fewer than that determined by HRMS data. Further analysis of the HMBC spectrum revealed a long-range HMBC correlation from CH_2_-12′ to C-1, suggesting an ether linkage between C-1 and C-3′. This additional ring satisfied the final degree of unsaturation, thereby completing the determination of the planar structure of **4**. The relative configuration of H-2′ and CH_2_-12′ was assigned as *syn* by NOESY correlations between H-2′/H_2_-12′ and H-2′/H_3_-13′ (Figure 4). Therefore, four possible absolute configurations could be assigned to **4**, namely, 1′*S*,2′*R*,3′*R* (**4a**), 1′*R*,2′*S*,3′*S* (**4b**), 1′*S*,2′*S*,3′*S* (**4c**), and 1′*R*,2′*R*,3′*R* (**4d**). Finally, the absolute configuration of **4** was assigned to be 1′*S*,2′*R*,3′*R*, given that the experimental ECD spectrum of **4** matched well with that calculated for the model compound **4a** but was different from that computed for **4b**, **4c**, and **4d** (Figure 5, Figures S7 and S8).

NEN I (**5**) was isolated as a purple amorphous powder. Its molecular formula was established to be C_43_H_33_NO_12_S through HRMS (*m/z*: 786.1658 [M − H]^−^, calcd for C_43_H_32_NO_12_S, 786.1651), indicating one oxygen atom fewer than that of **4**. A detailed comparison of the NMR data of **5** and **4** revealed their high structural similarity (Table 3 and Figure S9). A key difference was observed at the C-10′ position. Compound **4** features a sp^2^-hybridized and oxygenated carbon at C-10′ (*δ*_C_ 155.8), while compound **5** exhibited signals for a sp^2^-hybridized methine group at this position (*δ*_C_*/δ*_H_ 117.4/7.54, CH-10′). The assignment was confirmed by the COSY correlation between H-9′ (*δ*_H_ 7.47) and H-10′ (*δ*_H_ 7.54), and HMBC correlations from H-8′ to C-10′. The absolute configuration of **5** was assigned as 1′*S*,2′*R*,3′*R* based on the identical experimental ECD spectra of **5** and **4**, and their comparable specific rotation values: ${\left[\alpha \right]}_{D}^{25}$ = −251.0° for **5** and ${\left[\alpha \right]}_{D}^{25}$ = −262.7° for **4** (Figure S7). Finally, **5** was determined as a C-10′ dehydroxylation derivative of **4**.

Compound **6** was determined to be homo-dehydrorabelomycin E [12], based on comparing its NMR data with those previously reported (Figure S10).

Bioinformatic analysis identified Nes5 as a homologue (62.7% identity) of the deacylase FlsH (Figure S11) [8]. In the fluostatin biosynthetic pathway, FlsH is characterized to catalyze the hydrolysis of acyl fluostatins to prevent their spontaneous conversion into toxic quinone methides, which act as key transient intermediates that non-enzymatically drive the formation of fluostatin dimers [8]. We therefore hypothesize that disruption of the *nes5* gene would result in the accumulation of acyl nenestatins. However, no acyl nenestatins were detected in the Δ*nes5* mutant, implying that Nes5 does not possess a deacylase function similar to that of FlsH. Given that the abolished production of NENs A and B (Figure S1 for their structures) in the Δ*nes5* mutant, Nes5 should be essential for their biosynthesis. However, the exact function of Nes5 in the biosynthesis of NENs A and B requires further exploration.

**Figure 6:**
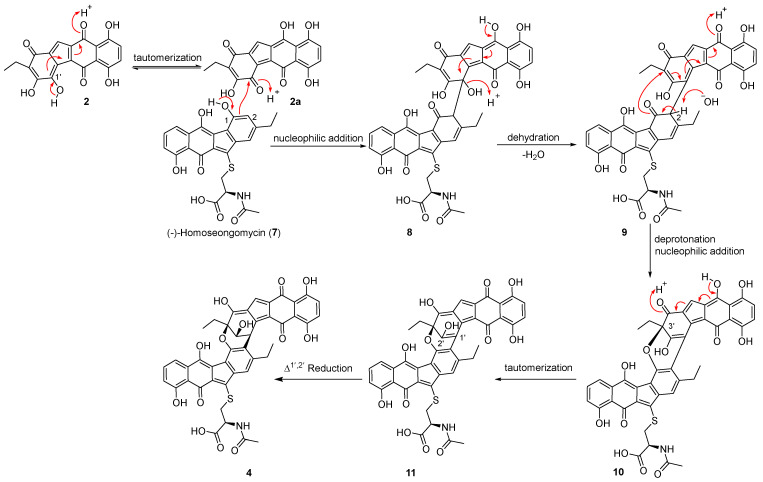
The proposed biosynthetic pathway of **4**.

Notably, five new nenestatin congeners, including two novel dimers, **4** and **5**, were isolated. This suggests that Nes5 may catalyze or regulate an as-yet-unidentified key step, and its absence leads to precursor diversion and the subsequent formation of **4** and **5**. Finally, we propose a plausible nonenzymatic way for the dimerization of compounds **4** and **5** (Figure 6) [11]. The formation of **4** is likely initiated by deprotonation of the 1-OH group in (-)-homoseongomycin (**7**) to generate a phenolate anion, which undergoes resonance and nucleophilic addition to **2a,** the keto-enol tautomer of compound **2**, forming the C-2–C-1′ bond in the intermediate **8**. Next, **8** undergoes tautomerization followed by dehydration to yield intermediate **9**. Deprotonation at CH-2 of **9** induces a nucleophilic attack by O-1 on C-3′ to form the key C-1–C-3′ ether bond in **10**. Finally, a tautomerization step and subsequent enzymatic or nonenzymatic reduction in the Δ^1′,2′^ double bond in the intermediate **11** affords compound **4**. Alternatively, a coupling reaction between **7** and **1** could lead to the formation of compound **5**. The proposed formation of **4** and **5** resembles the nonenzymatic C−C coupling to produce several natural product artifacts [8,15]. However, the proposed pathway requires further in vitro chemical experiments to confirm.

The biosynthetic machinery of compound **3** is likely encoded by the *nes* BGC. The *nes* chain length factor (CLF) probably exhibits loose control in the chain length, and thus allows the formation of both decaketide and nonaketide precursors [16,17,18]. Therefore, we propose that a shunt nonaketide intermediate is processed via a pathway analogous to that of 4-acetylchrysophanol to yield compound **3**, as previously observed in the heterologous expression of the fluostatin BGC (Figure S12) [19].

The compounds **1**–**6** were evaluated for antibacterial activity against six bacterial strains (*Staphylococcus aureus* ATCC 29213, *Enterococcus faecalis* ATCC 29212, *Acinetobacter baumannii*, *Klebsiella pneumoniae* ATCC 13883, *Mycobacterium smegmatis* MC^2^ 155, and *Micrococcus luteus*) via broth microdilution [20]. None of the compounds showed inhibitory activity at a concentration of 64 µg·mL^−1^. Separately, the cytotoxicity of compounds **1**–**5** was assessed against four human cancer cell lines (SF-268, HepG-2, MCF-7, A549) using the SRB method [21]. None of the compounds exhibited cytotoxicity against these cell lines at concentrations up to 100 µM. All tested compounds were inactive in both the antibacterial and cytotoxicity assays under the conditions employed, indicating that their structural features do not confer activity against the bacterial strains or cell lines tested. Future studies could focus on structural diversification to enhance bioactivity or on screening against additional biological targets.

## 3. Materials and Methods

### 3.1. General Experimental Procedures

Optical rotations were measured with an MCP 500 polarimeter (Anton, Graz, Austria). UV spectra were recorded on a UV-2600 spectrophotometer (Shimadzu, Kyoto, Japan). Circular dichroism (CD) spectra were recorded on a Chirascan CD spectropolarimeter (Applied Photophysics Ltd., Surrey, UK). IR spectra were measured on an IR Affinity-1 FT-IR spectrometer (Shimadzu, Kyoto, Japan). ^1^H, ^13^C, and 2D NMR spectra were recorded on a Bruker AVANCE III HD 700 MHz NMR spectrometer (Bruker Biospin GmbH & Co. KG., Rheinstetten, Baden-Württemberg, Germany), with TMS as an internal standard. HRESIMS data were measured using a MaXis 4G UHR-TOFMS spectrometer (Bruker Daltonics GmbH & Co. KG., Bremen, Germany). Materials for column chromatography (CC) were silica gel (100–200 mesh; 300–400 mesh; Jiangyou Silica Gel Development Co., Ltd., Yantai, Shandong, China), Sephadex LH-20 (40–70 μm; Amersham Pharmacia Biotech AB, Uppsala, Sweden), and YMC*GEL ODS-A (12 nm S-50 μm; YMC Company Ltd., Kyoto, Japan). Thin layer chromatography (TLC, 0.1–0.2 or 0.3–0.4 mm) was conducted with precoated glass plates (silica gel GF254, 10–40 nm, Jiangyou Silica Gel Development Co., Ltd., Yantai, Shandong, China), Medium pressure liquid chromatography (MPLC) was performed with automatic flash chromatography (Cheetahtmmp 200, Bonna-Agela Technologies Co., Ltd., Tianjin, China) with a monitoring wavelength of 220 nm and a collecting wavelength of 254 nm. Semipreparative HPLC was performed on a Hitachi HPLC station (Hitachi-L2130, Hitachi, Tokyo, Japan) with a diode array detector (Hitachi L-2455, Hitachi, Tokyo, Japan) using a ODS column (Kinetex C18, 250 mm × 10.0 mm, 5 μm; Phenomenex, Torrance, California, USA).

### 3.2. Construction of M. echinospora SCSIO 04089/Δnes5 Mutant

To generate a *nes5* in-frame deletion mutant, two homologous DNA fragments flanking the target gene were amplified from *M. echinospora* SCSIO 04089 genomic DNA using primer pairs Nes5-UF/Nes5-UR and Nes5-DF/Nes5-DR (Table S1). The PCR products were purified and ligated into the pre-digested thermal-sensitive *Streptomyces*–*Escherichia coli* shuttle vector pKC1139, resulting in the construction of the gene knockout plasmid pKC1139/Δ*nes5*. Following sequence confirmation, the deletion plasmid was introduced into *E. coli* ET12567/pUZ8002 by transformation and subsequently transferred into *M. echinospora* SCSIO 04089 via conjugation. Exconjugants were cultured at 28 °C for two successive generations to facilitate double-crossover recombination and then shifted to 37 °C to cure the plasmid. Gene knockout was verified by PCR using the flanking primers Nes5-TF and Nes5-TR (Table S1).

### 3.3. Fermentation, Extraction, and Isolation

The mutant strain *M. echinospora* SCSIO 04089*/*Δ*nes5* was cultured on an ATCC172 agar plate (soluble starch 20.0 g L^−1^, glucose 10.0 g L^−1^, yeast extract 5.0 g L^−1^, Aobox casein 5.0 g L^−1^, CaCO_3_ 19.0 g L^−1^, artificial sea salt 10.0 g L^−1^, pH 7.0) at 28 °C for 7 days. A piece of mycelia was then inoculated into a 250 mL Erlenmeyer flask containing 50 mL of A1 medium (soluble starch 10.0 g L^−1^, yeast extract 4.0 g L^−1^, bacterial peptone 2.0 g L^−1^, artificial sea salt 10.0 g L^−1^, pH 7.0) and incubated at 28 °C with shaking at 200 rpm for 3 days to prepare seed cultures. The seed cultures were subsequently transferred into 1000 mL Erlenmeyer flask containing 200 mL of the N4 medium (soluble starch 15.0 g L^−1^, fish peptone 8.0 g L^−1^, bacterial peptone 5.0 g L^−1^, glycerol 7.5 g L^−1^, CaCO_3_ 2.0 g L^−1^, KBr 0.2 g L^−1^, artificial sea salt 30.0 g L^−1^, prewashed HP20 resin 5%, *v/v*, pH 7.0). A total of 21 L of culture was prepared and incubated under the same conditions (28 °C, 200 rpm) for 7 days. During fermentation, the secondary metabolites were adsorbed by the HP20 resin, which changed color from white to black. The colored HP20 resin was collected by filtration and washed several times with 4 L of CH_3_CN. The combined solvents were concentrated under vacuum to yield 3.5 g of crude extract. The extract was fractionated by MPLC with an ODS column. Elution was performed using a linear gradient under the following program: solvent system (solvent A, H_2_O containing 5% CH_3_OH; solvent B, CH_3_OH); 0% B to 60% B (0–40 min), 60% B to 85% B (40–80 min), 85% B to 100% B (80–100 min), 100% B (100–120 min); flow rate at 20 mL·min^−1^ to yield seven fractions (Fr.1–Fr.7). Further purification of Fr.1–Fr.7 was carried out by semi-preparative HPLC (Kinetex C18, 250 mm × 10.0 mm, 5 μm; Phenomenex, Torrance, California, USA) using a linear gradient under the following program: solvent system (solvent A, H_2_O; solvent B, CH_3_CN); 10% to 80% B (0–25 min), 100% B (25.1–30 min), 100% B to 10% B (30–30.1 min), 10%B (30.1–35 min); flow rate at 2.5 mL min^−1^. This process resulted in the isolation of compounds **1** (4.6 mg, *t*_R_ = 21.0 min) and **2** (2.1 mg, *t*_R_ = 20.3 min) from Fr.2, compound **3** (7.1 mg, *t*_R_ = 23.0 min) from Fr.3, compounds **4** (2.6 mg, *t*_R_ = 24.7 min) and **5** (5.4 mg, *t*_R_ = 27.2 min) from Fr.4, and compound **6** (6.6 mg, *t*_R_ = 28.8 min) from Fr.7.

**Compound 1**: red amorphous powder. UV (CH_3_OH) *λ*_max_ (log *ε*) 209 (2.74), 278 (2.01), 424 (0.74), 488 (0.60) nm; IR (film) *ν*_max_ 3750, 3387, 1560, 1456, 1313, 1213, 1004 cm^−1^; ^1^H and ^13^C NMR data, Table 1; HRESIMS *m/z*: 335.0567 [M − H]^−^ (calcd for C_19_H_11_O_6_, 335.0561).

**Compound 2**: red amorphous powder. UV (CH_3_OH) *λ*_max_ (log *ε*) 205 (2.16), 277 (1.17) nm; IR (film) *ν*_max_ 3749, 3444, 1682, 1541, 1206, 1026 cm^−1^; ^1^H and ^13^C NMR data, Table 1; HRESIMS *m/z*: 351.0515 [M − H]^−^ (calcd for C_19_H_11_O_7_, 351.0510).

**Compound 3**: brown amorphous powder. ${\left[\alpha \right]}_{D}^{25}\;$= −21.0° (*c* 0.03, CH_3_OH); UV (CH_3_OH) *λ*_max_ (log *ε*) 223 (2.93), 268 (2.38), 407 (0.73) nm; IR (film) *ν*_max_ 3749, 1690, 1541, 1204, 1024 cm^−1^; ^1^H and ^13^C NMR data, Table 1; HRESIMS *m/z*: 313.1081 [M − H]^−^ (calcd for C_18_H_17_O_5_, 313.1081).

**Compound 4**: purple amorphous powder. ${\left[\alpha \right]}_{D}^{25}$ = −262.7° (*c* 0.03, CH_3_OH); UV (CH_3_OH) *λ*_max_ (log *ε*) 207 (2.36), 284 (1.24), 327 (0.90), 508 (0.60) nm; IR (film) *ν*_max_ 3408, 1681, 1204, 1024, 710 cm^−1^; ^1^H and ^13^C NMR data, Table 2; HRESIMS *m/z*: 802.1607 [M − H]^−^ (calcd for C_43_H_32_NO_13_S, 802.1600).

**Compound 5**: purple amorphous powder. ${\left[\alpha \right]}_{D}^{25}$ *=* −251.0° (*c* 0.03, CH_3_OH); UV (CH_3_OH) λ_max_ (log ε) 207 (2.26), 284 (1.18), 330 (0.88), 498 (0.50) nm; IR (film) *ν*_max_ 3397, 1672, 1204, 1024, 709 cm^−1^; ^1^H and ^13^C NMR data, Table 3; HRESIMS *m/z*: 786.1658 [M − H]^−^ (calcd for C_43_H_32_NO_12_S, 786.1651).

**Compound 6**: brown amorphous powder. UV (CH_3_OH) λ_max_ (log ε) 208 (2.62), 232 (2.30), 451 (0.44) nm; IR (film) *ν*_max_ 3741, 1601, 1456, 1250, 746 cm^−1^; HRESIMS *m/z*: 333.0076 [M − H]^−^ (calcd for C_20_H_13_O_5_, 333.0768).

### 3.4. TDDFT-ECD Calculations

All quantum chemical calculations were performed using the Gaussian 09 (Revision D.01) software package [22]. Conformational searches were conducted using the Molecular Merck Force Field (MMFF) as implemented in Spartan’14 V1.1.4 software (Wavefunction Inc., Irvine, CA, USA). For compound **3**, all conformers with a population greater than 1% were re-optimized at the B3LYP/6-311G (d,p) level of theory, while for compound **4**, the CAM-B3LYP/6-311G(d,p) level was employed. These geometry optimizations utilized the IEFPCM solvation model to simulate a CH_3_OH environment. Subsequently, time-dependent density functional theory (TDDFT) calculations at the corresponding theory levels (i.e., B3LYP/6-311G (d,p)/IEFPCM(CH_3_OH) for **3** and CAM-B3LYP/6-311G(d,p)/IEFPCM(CH_3_OH) for **4**) were conducted to obtain the ECD spectra for the stable conformers. Finally, the overall theoretical ECD spectrum for each compound was generated by Boltzmann averaging the individual conformer spectra using SpecDis 1.71 [23].

### 3.5. Antibacterial Assays

The antibacterial activities of the compounds **1**–**6** were evaluated against six indicator strains: *Staphylococcus aureus* ATCC 29213, *Enterococcus faecalis* ATCC 29212, *Acinetobacter baumannii*, *Klebsiella pneumoniae* ATCC 13883, *Mycobacterium smegmatis* MC^2^-155, and *Micrococcus luteus*, using the broth microdilution method [20]. Indicator strains were grown on a rotary shaker at 37 °C for 12 h. The cultures were diluted with sterilized medium to an optical density (OD_600_) of 0.04–0.06 and then further diluted 1000-fold before being dispensed into 96-well microtiter plates. Each compound was tested in triplicate over a dilution series ranging from 64 to 0.25 µg mL^−1^. After 16 h of incubation, the minimum concentrations that completely inhibited visible growth of the tested strains were determined from two independent experiments.

### 3.6. Cytotoxic Activity Assays

The cytotoxicities of compounds **1**–**5** were evaluated against SF-268 (human glioma cell line), HepG-2 (human liver carcinoma cell line), MCF-7 (human breast adenocarcinoma cell line), and A549 (human lung adenocarcinoma cell line) (The cell lines were purchased from the National Collection of Authenticated Cell Cultures in Shanghai, China) by the SRB method [21]. The cells were cultivated in RPMI 1640 medium [24]. Cells (180 µL) with a density of 3 × 10^4^ cells mL were seeded onto 96-well plates and incubated for 24 h at 37 °C, 5% CO_2_. Subsequently, 20 µL of different concentrations of **1**–**5**, ranging from 0 to 100 µM in DMSO, was added to each plate well. An equal volume of DMSO was used as a negative control. After further incubation for 72 h, the cell monolayers were fixed with 50% (*w/v*) trichloroacetic acid (50 µL) and then stained for 30 min with 0.4% (*w/v*) SRB dissolved in 1% acetic acid. The unbound dye was removed by repeatedly washing with 1% acetic acid. The protein-bound dye was dissolved in a 10 mM Tris-base solution (200 µL) for the determination of the OD_570_ value using a microplate reader. The cytotoxic compound cisplatin was used as a positive control. All data were obtained in triplicate and are presented as means ± S.D. IC_50_ values were calculated with the SigmaPlot 14.0 software using the non-linear curve-fitting method.

## Figures and Tables

**Table 1:** ^1^H (700 MHz) and ^13^C NMR data (175 MHz) for **1**–**3** in DMSO-*d*_6_.

No.	1		2			3	
	*δ*_C_, Type	*δ*_H_, Mult (*J* in Hz)	*δ*_C_, Type	*δ*_H_, Mult (*J* in Hz)		*δ*_C_, Type	*δ*_H_, Mult (*J* in Hz)
1	175.1, C		175.2, C		1	60.7, CH	4.33, s
2	156.1, C		156.0, C		2	72.3, C	
2-OH				9.59, s			
3	120.2, C		120.1, C		3	45.2, CH_2_	2.60, d (18.2); 3.12, d (17.5)
4	183.9, C		183.9, C		4	204.3, C	
4a	128.4, C		128.9, C		4a	109.0, C	
5	115.0, CH	6.88, s	115.4, CH	6.92, s	5	164.6, C	
5a	130.9, C		131.8, C		5-OH		15.7
6	186.6, C		183.4, C		5a	112.6, C	
6a	116.6, C		115.2, C		6	157.3, C	
7	161.1, C		156.2, C		6-OH		9.81
7-OH		13.49, s		14.07, s	7	111.1, CH	6.85, d (7.7)
8	121.5, CH	7.05, dd (7.7, 1.4)	127.0, CH	7.09, d (9.1)	8	132.4, CH	7.53, dd (7.7, 7.7)
9	134.6, CH	7.53, dd (7.7)	126.2, CH	7.08, d (9.1)	9	118.2, CH	7.25, d (7.7)
10	118.0, CH	7.50, dd (7.7, 1.4)	155.6, C		9a	138.9, C	
10-OH				13.58, s	10	118.6, CH	7.29, s
10a	137.0, C		114.1, C		10a	109.0, C	
11	177.1, C		185.3, C		11	32.1, CH_2_	1.51, m; 1.64, m
11a	125.7, C		124.1, C		12	6.80, CH_3_	0.97, t (7.0)
11b	121.4, C		121.7, C		13	207.2, C	
12	15.8, CH_2_	2.32, q (7.7)	15.7, CH_2_	2.32, q (7.0)	14	32.0, CH_3_	2.38, s
13	13.1, CH_3_	0.97, t (7.7)	13.1, CH_3_	0.97, t (7.0)			

**Table 2:** ^1^H (700 MHz) and ^13^C NMR data (175 MHz) for **4** in DMSO-*d*_6_.

No.	4 (Unit A)		No.	4 (Unit B)	
	*δ*_C_, Type	*δ*_H_, Mult (*J* in Hz)		*δ*_c_, Type	*δ*_H_, Mult (*J* in Hz)
1	145.6, C		1′	36.1, CH	5.12, d (2.8)
2	126.5, C		2′	67.3, CH	4.44, overlap
			2′-OH		5.92, s
3	144.2, C		3′	81.6, C	
4	118.5, CH	7.27, s	4′	186.2, C	
4a	120.1, C		4a′	125.7, C	
5	146.9, C		5′	119.8, CH	7.00, overlap
5a	128.1, C		5a′	125.8, C	
6	183.4, C		6′	184.0, C	
6a	115.6, C		6a′	115.4, C	
7	162.8, C		7′	155.6, C	
7-OH		13.42, s	7′-OH		13.84, s
8	119.8, CH	7.00, overlap	8′	125.8, CH	7.00, overlap
9	136.1, CH	7.57, dd (8.4, 7.7)	9′	125.9, CH	7.00, overlap
10	116.3, CH	7.41, dd (7.7, 0.7)	10′	155.8, C	
			10′-OH		14.16, s
10a	133.2, C		10a′	116.2, C	
11	147.7, C		11′	183.4, C	
11-OH		10.65, s			
11a	113.5, C		11a′	120.2, C	
11b	139.9, C		11b′	141.8, C	
12	25.6, CH_2_	3.40, m; 2.95, m	12′	22.1, CH_2_	2.41, m; 1.98, m
13	16.3, CH_3_	1.16, t (7.0)	13′	6.19, CH_3_	1.02, t (7.0)
14	34.7, CH_2_	3.88, dd (13.3, 4.2); 3.50, m			
15	52.5, CH	4.44, overlap			
16	171.6, C				
NH		8.27, d (8.4)			
17	169.4, C				
18	22.1, CH_3_	1.68, s			

**Table 3:** ^1^H (700 MHz) and ^13^C NMR data (175 MHz) for **5** in DMSO-*d*_6_.

No.	5 (Unit A)		No.	5 (Unit B)	
	*δ*_C_, Type	*δ*_H_, Mult (*J* in Hz)		*δ*_C_, Type	*δ*_H_, Mult (*J* in Hz)
1	145.6, C		1′	35.6, CH	5.14, d (2.8)
2	126.8, C		2′	67.3, CH	4.44, overlap
			2′-OH		5.86, s
3	144.4, C		3′	81.7, C	
4	118.5, CH	7.24, s	4′	186.0, C	
4a	120.0, C		4a′	125.0, C	
5	147.0, C		5′	116.9, CH	6.96, s
5a	125.5, C		5a′	128.1, C	
6	183.4, C		6′	184.9, C	
6a	115.6, C		6a′	117.8, C	
7	162.8, C		7′	161.6, C	
7-OH		13.43, s	7′-OH		13.98, s
8	119.8, CH	6.99, d (8.4)	8′	121.0, CH	6.98, dd (7.7, 1.4)
9	136.1, CH	7.57, dd (8.4, 7.7)	9′	134.1, CH	7.47, dd (7.7, 7.7)
10	116.3, CH	7.40, d (7.7)	10′	117.4, CH	7.54, dd (7.7, 1.4)
10a	133.3, C		10a′	138.2, C	
11	147.7, C		11′	178.1, C	
11-OH		10.67, s			
11a	113.6, C		11a′	121.2, C	
11b	139.8, C		11b′	140.8, C	
12	25.6, CH_2_	3.57, m; 2.85, m	12′	22.1, CH_2_	2.40, m; 1.96, m
13	16.5, CH_3_	1.16, t (7.0)	13′	6.2, CH_3_	1.00, t (7.7)
14	34.7, CH_2_	3.87, dd (13.3, 4.9); 3.50, m			
15	52.5, CH	4.44, overlap			
16	171.5, C				
NH		8.27, d (8.4)			
17	169.4, C				
18	22.1, CH_3_	1.68, s			

## Data Availability

The original data presented in the study are included in the article and Supplementary Materials; further inquiries can be directed to the corresponding authors.

## Supplementary Materials

The following supporting information can be downloaded at https://www.mdpi.com/article/10.3390/md24030103/s1. Figure S1: HPLC profile of the crude extracts from *M. echinospora* SCSIO 04089 and Δ*nes5* mutant fermented on N4 medium with HP20 resin; Figure S2: The spectroscopic data of NEN E (**1**); Figure S3: The spectroscopic data of NEN F (**2**); Figure S4: The spectroscopic data of NEN G (**3**); Figure S5: The optimized conformers above 1% population of **3a**; Figure S6: The spectroscopic data of NEN H (**4**); Figure S7: Comparison of the Exp ECD of **4** and **5** with those Calcd for **4c** and **4d**; Figure S8: The optimized conformers above 1% population of **4a**; Figure S9: The spectroscopic data of NEN I (**5**); Figure S10: The spectroscopic data of homo-dehydrorabelomycin E (**6**); Figure S11: Sequence alignment of Nes5, Lom6, FlsH and Alp1U; Figure S12: The proposed biosynthetic pathway of **3**; Table S1: List of primers used in this study; Table S2: Cartesian coordinates for the re-optimized conformers of **3a** at the B3LYP/6-311G(d,p) level in CH_3_OH; Table S3: Cartesian coordinates for the re-optimized conformers of **4a** at the CAM-B3LYP/6-311G(d,p) level in CH_3_OH; Table S4: Imaginary frequencies and absolute energy values of the re-optimized conformers of **3a** at the B3LYP/6-311G(d,p) level in CH_3_OH; Table S5: Imaginary frequencies and absolute energy values of the re-optimized conformers of **4a** at the CAM-B3LYP/6-311G(d,p) level in CH_3_OH.
