# Supplementary material for: Targeted Inactivation of an α/β Hydrolase Gene Enables Discovery of Heterodimeric Nenestatins
Source: Mar Drugs. 2026 Mar 8;24(3):103. doi: 10.3390/md24030103 (PMC13028485; doi:10.3390/md24030103)

Supporting Information

Targeted Inactivation of an α/β Hydrolase Gene Enables Discovery of Heterodimeric Nenestatins

Wenzheng Wei [1,2,†], Xiaodong Jiang [3,†], Yiguang Zhu [1,2], Wenjun Zhang [1,2], Chunfang Yang [1,2], Qingbo Zhang [1,2,*], and Changsheng Zhang [1,2,*]

[1] State Key Laboratory of Tropical Oceanography, Guangdong Key Laboratory of Marine Materia Medica, South China Sea Institute of Oceanology, Chinese Academy of Sciences, 164 West Xingang Road, Guangzhou 510301, China; weiwenzheng23@mails.ucas.ac.cn (W.W.); ygzhu@scsio.ac.cn (Y.Z.); wzhang@scsio.ac.cn (W.Z.); yangchunfang@scsio.ac.cn (C.Y.)

[2] University of Chinese Academy of Sciences, Beijing 100049, China

[3] Guangxi Key Laboratory of Marine Drugs, University Engineering Research Center of High-efficient Utilization of Marine Traditional Chinese Medicine Resources, Institute of Marine Drugs, Guangxi University of Chinese Medicine, Nanning 530200, China; jxd374487986@163.com (X.J.)

* Correspondence: zhangqingbo@scsio.ac.cn (Q.Z.); czhang@scsio.ac.cn (C.Z.); Tel.: +86-20-8902-3038 (C.Z.)

† These authors contribute equally to this work.

Content

Table S1. List of primers used in this study. 3

Table S2. Cartesian coordinates for the re-optimized conformers of **3a** at the B3LYP/6-311G(d,p) level in CH_3OH. 4

Table S3. Cartesian coordinates for the re-optimized conformers of **4a** at the CAM-B3LYP/6-311G(d,p) level in CH_3OH. 16

Table S4. Imaginary frequencies and absolute energy values of the re-optimized conformers of **3a** at the B3LYP/6-311G(d,p) level in CH_3OH. 19

Table S5. Imaginary frequencies and absolute energy values of the re-optimized conformers of **4a** at the CAM-B3LYP/6-311G(d,p) level in CH_3OH. 21

Figure S1. HPLC profile of the crude extracts from *M. echinospora* SCSIO 04089 and Δ*nes5* mutant fermented on N4 medium with HP20 resin. 22

Figure S2. The spectroscopic data of NEN E (**1**). 23

Figure S3. The spectroscopic data of NEN F (**2**). 32

Figure S4. The spectroscopic data of NEN G (**3**). 42

Figure S5. The optimized conformers above 1% population of **3a**. 55

Figure S6. The spectroscopic data of NEN H (**4**). 56

Figure S7. Comparison of the Exp ECD of **4** and **5** with those Calcd for 4c and 4d... 66

Figure S8. The optimized conformers above 1% population of **4a**. 67

Figure S9. The spectroscopic data of NEN I (**5**). 68

Figure S10. The spectroscopic data of homo-dehydrorabelomycin E (**6**). 78

Figure S11. Sequence alignment of Nes5, Lom6, FlsH and Alp1U. 82

Figure S12. The proposed biosynthetic pathway of **3**. 83

Table S1. List of primers used in this study.

Primers	Restriction sites	Sequences
Nes5-UF	*Hind*III	5'TATGACATGATTACGAATTCCGGCGACG CAGACGGACCG3'
Nes5-UR		5'GCGAGGATTTCGGTCTCCACGGCTCGTT CATAGGCGATC3'
Nes5-DF	*EcoR*I	5'GACGGCCAGTGCCAAGCTTGGAGGCTC GCCGCAGCAAGA3'
Nes5-DR		5'GATCGCCTATGAACGAGCCGTGGAGACC GAAATCCTCGC3'
Nes5-TF		5'TGTCACGCTACTGACAGAC3'
Nes5-TR		5'GCAAGGTCGTTTCGCGGGAC3'

Table S2. Cartesian coordinates for the re-optimized conformers of **3a** at the B3LYP/6-311G(d,p) level in CH_3OH.

**3a** Conf. 1			Coordinates (Angstroms)		
Center Number	Atomic Number	Atomic Type	X	Y	Z
1	8	0	-0.5104	7.6958	4.3817
2	8	0	-4.7614	6.419	5.4639
3	6	0	-5.8753	5.2561	3.7295
4	6	0	-5.0108	6.3765	4.281
5	8	0	0.2682	6.4407	2.2212
6	8	0	0.8486	5.0873	0.1363
7	6	0	-4.4818	7.4163	3.2995
8	6	0	-3.9791	8.7326	3.9204
9	6	0	-2.6884	8.494	4.7154
10	6	0	-1.6492	7.6795	3.9691
11	6	0	-3.7663	6.1273	1.2728
12	6	0	-3.3999	6.8144	2.4286
13	6	0	-2.0433	6.9246	2.7446
14	6	0	-1.0589	6.3433	1.9268
15	6	0	-3.2268	4.8646	-0.6976
16	6	0	-2.8066	5.5458	0.4466
17	6	0	-1.4475	5.6475	0.7684
18	6	0	-0.4959	5.0507	-0.0801
19	6	0	-0.9406	4.3746	-1.2193
20	6	0	-2.2902	4.2742	-1.5381
21	8	0	-3.6392	9.5718	2.8361
22	6	0	-5.0266	9.4769	4.7772
23	6	0	-6.3368	9.8086	4.0495
24	1	0	-6.3485	4.6756	4.5541
25	1	0	-6.6897	5.6672	3.0912
26	1	0	-5.2567	4.5524	3.1285
27	1	0	0.4464	6.9122	3.0523
28	1	0	1.0706	5.5674	0.9503

29	1	0	-5.3436	7.6827	2.6455
30	1	0	-2.8944	7.9729	5.6772
31	1	0	-2.2359	9.4775	4.9843
32	1	0	-4.8348	6.0429	1.0136
33	1	0	-4.2971	4.7828	-0.9529
34	1	0	-0.1996	3.9049	-1.888
35	1	0	-2.6129	3.7339	-2.4442
36	1	0	-3.4295	10.4386	3.1491
37	1	0	-5.2665	8.8869	5.6916
38	1	0	-4.5776	10.4319	5.1407
39	1	0	-6.9956	10.4406	4.6894
40	1	0	-6.1487	10.3705	3.1062
41	1	0	-6.9162	8.8906	3.8007
B3LYP/6-311G(d,p) Energy = -1073.2055118 a.u.; Population = 18.34%					

**3a** Conf. 2			Coordinates (Angstroms)		
Center Number	Atomic Number	Atomic Type	X	Y	Z
1	8	0	-0.6901	7.7126	4.4118
2	8	0	-4.7666	6.3484	5.4203
3	6	0	-5.7966	5.042	3.735
4	6	0	-5.0244	6.2474	4.2427
5	8	0	0.2077	6.5197	2.2552
6	8	0	0.8961	5.178	0.1939
7	6	0	-4.5924	7.2929	3.22
8	6	0	-4.1658	8.6653	3.7804
9	6	0	-2.8794	8.5336	4.6046
10	6	0	-1.8068	7.6869	3.943
11	6	0	-3.787	6.0232	1.2101
12	6	0	-3.4755	6.7341	2.367
13	6	0	-2.134	6.9054	2.7155
14	6	0	-1.1056	6.3613	1.9275
15	6	0	-3.1444	4.7717	-0.7361
16	6	0	-2.7824	5.4789	0.4122
17	6	0	-1.4368	5.642	0.7656
18	6	0	-0.4399	5.0814	-0.055
19	6	0	-0.8269	4.3786	-1.199
20	6	0	-2.1628	4.2171	-1.5491
21	8	0	-3.8634	9.4564	2.6496
22	6	0	-5.3216	9.3531	4.5396
23	6	0	-5.0016	10.741	5.1137
24	1	0	-6.1973	4.4396	4.582
25	1	0	-6.658	5.3646	3.1076
26	1	0	-5.131	4.3839	3.1327
27	1	0	0.3406	6.9919	3.0946
28	1	0	1.0766	5.6708	1.0106
29	1	0	-5.4874	7.4768	2.5799
30	1	0	-3.0889	8.1	5.6081

31	1	0	-2.4441	9.544	4.7835
32	1	0	-4.8453	5.8891	0.9305
33	1	0	-4.2037	4.6413	-1.0162
34	1	0	-0.0502	3.937	-1.846
35	1	0	-2.4396	3.6568	-2.4583
36	1	0	-3.5472	10.3064	2.9117
37	1	0	-6.1959	9.4506	3.8529
38	1	0	-5.6539	8.7068	5.3842
39	1	0	-5.8969	11.1746	5.6171
40	1	0	-4.1918	10.6959	5.8768
41	1	0	-4.6944	11.4591	4.3201
B3LYP/6-311G(d,p) Energy = -1073.20533 a.u.; Population = 15.15%					

**3a** Conf. 3			Coordinates (Angstroms)		
Center Number	Atomic Number	Atomic Type	X	Y	Z
1	8	0	-0.6479	7.5808	4.4932
2	8	0	-4.8252	6.411	5.4565
3	6	0	-5.7168	5.0631	3.7267
4	6	0	-5.0073	6.2927	4.2667
5	8	0	0.2329	6.4545	2.2928
6	8	0	0.8927	5.1517	0.1971
7	6	0	-4.5451	7.3406	3.2597
8	6	0	-4.0783	8.6907	3.8392
9	6	0	-2.8011	8.5066	4.6689
10	6	0	-1.7559	7.6278	4.0057
11	6	0	-3.7692	6.1041	1.2162
12	6	0	-3.4428	6.7723	2.3942
13	6	0	-2.0987	6.8943	2.753
14	6	0	-1.0825	6.3429	1.9547
15	6	0	-3.153	4.8895	-0.7617
16	6	0	-2.7766	5.5533	0.4077
17	6	0	-1.4286	5.6668	0.7714
18	6	0	-0.4441	5.1008	-0.0604
19	6	0	-0.8455	4.4421	-1.2255
20	6	0	-2.1837	4.3295	-1.5857
21	8	0	-3.7128	9.498	2.7389
22	6	0	-5.194	9.4247	4.6144
23	6	0	-4.8214	10.8383	5.0844
24	1	0	-4.9995	4.4159	3.1737
25	1	0	-6.1541	4.4591	4.5542
26	1	0	-6.5454	5.3582	3.044
27	1	0	0.374	6.9092	3.1407
28	1	0	1.0834	5.6184	1.0267
29	1	0	-5.4365	7.5583	2.6256
30	1	0	-3.0305	8.0707	5.6673

31	1	0	-2.3302	9.4995	4.8555
32	1	0	-4.8296	6.0089	0.9287
33	1	0	-4.2141	4.798	-1.0501
34	1	0	-0.0786	3.9965	-1.8814
35	1	0	-2.4719	3.8034	-2.5116
36	1	0	-4.483	9.8364	2.3098
37	1	0	-6.0984	9.4989	3.9649
38	1	0	-5.4959	8.8295	5.5066
39	1	0	-5.6926	11.3358	5.5705
40	1	0	-3.9985	10.8209	5.8344
41	1	0	-4.5029	11.4834	4.2339
B3LYP/6-311G(d,p) Energy = -1073.2063148 a.u.; Population = 43.04%					

**3a** Conf. 4			Coordinates (Angstroms)		
Center Number	Atomic Number	Atomic Type	X	Y	Z
1	8	0	-0.4763	7.5646	4.4656
2	8	0	-4.8477	6.5237	5.4959
3	6	0	-5.7804	5.2745	3.7164
4	6	0	-5	6.4399	4.2991
5	8	0	0.2878	6.3732	2.2639
6	8	0	0.8438	5.0588	0.1475
7	6	0	-4.4362	7.4734	3.3301
8	6	0	-3.8929	8.7664	3.9646
9	6	0	-2.621	8.4691	4.773
10	6	0	-1.6055	7.6218	4.0306
11	6	0	-3.7459	6.2162	1.2731
12	6	0	-3.3682	6.8586	2.4508
13	6	0	-2.0117	6.9161	2.7808
14	6	0	-1.0389	6.3259	1.9557
15	6	0	-3.2288	4.9909	-0.7266
16	6	0	-2.7977	5.6266	0.4394
17	6	0	-1.439	5.6752	0.7753
18	6	0	-0.4992	5.0715	-0.0813
19	6	0	-0.9547	4.4417	-1.2424
20	6	0	-2.3038	4.3938	-1.5752
21	8	0	-3.4744	9.605	2.9072
22	6	0	-4.8979	9.5591	4.8296
23	6	0	-6.2722	9.8108	4.1934
24	1	0	-6.2883	4.6935	4.5196
25	1	0	-6.5629	5.6409	3.014
26	1	0	-5.0954	4.583	3.1763
27	1	0	0.4731	6.8242	3.105
28	1	0	1.0738	5.5101	0.9756
29	1	0	-5.2909	7.7718	2.6804
30	1	0	-2.8567	7.9421	5.7249

31	1	0	-2.1337	9.4315	5.0576
32	1	0	-4.8142	6.1734	1.0031
33	1	0	-4.2988	4.9508	-0.9932
34	1	0	-0.2231	3.9668	-1.9177
35	1	0	-2.6352	3.8893	-2.4987
36	1	0	-4.2165	9.9493	2.438
37	1	0	-5.0604	9.0344	5.7994
38	1	0	-4.4416	10.5443	5.0883
39	1	0	-6.8995	10.4545	4.853
40	1	0	-6.1877	10.3295	3.2118
41	1	0	-6.8365	8.8627	4.0407
B3LYP/6-311G(d,p) Energy = -1073.2053494 a.u.; Population = 15.47%					

**3a** Conf. 5			Coordinates (Angstroms)		
Center Number	Atomic Number	Atomic Type	X	Y	Z
1	8	0	-0.5294	7.7199	4.4129
2	8	0	-4.5486	6.2873	5.3891
3	6	0	-6.1179	5.4792	3.8041
4	6	0	-5.0096	6.4027	4.2767
5	8	0	0.2563	6.4741	2.2546
6	8	0	0.8456	5.1321	0.1662
7	6	0	-4.4983	7.4593	3.3047
8	6	0	-4.0115	8.7652	3.9558
9	6	0	-2.6972	8.5573	4.7224
10	6	0	-1.6658	7.7161	3.9948
11	6	0	-3.7729	6.1822	1.2778
12	6	0	-3.4109	6.864	2.4379
13	6	0	-2.0566	6.9659	2.766
14	6	0	-1.0694	6.3845	1.9515
15	6	0	-3.2248	4.9289	-0.6963
16	6	0	-2.8099	5.602	0.4546
17	6	0	-1.4527	5.6974	0.7862
18	6	0	-0.4976	5.1021	-0.0595
19	6	0	-0.9369	4.4346	-1.2057
20	6	0	-2.2846	4.3405	-1.5341
21	8	0	-3.7703	9.6937	2.9178
22	6	0	-5.0596	9.427	4.8785
23	6	0	-6.4093	9.7312	4.2127
24	1	0	-6.4656	4.8181	4.6304
25	1	0	-6.9915	6.0707	3.4481
26	1	0	-5.7535	4.8304	2.9765
27	1	0	0.4324	6.9113	3.1048
28	1	0	1.0646	5.5969	0.9898
29	1	0	-5.3576	7.7309	2.6494
30	1	0	-2.8805	8.0687	5.7059

31	1	0	-2.2448	9.5519	4.9472
32	1	0	-4.8401	6.1021	1.012
33	1	0	-4.2937	4.8522	-0.9592
34	1	0	-0.1931	3.9663	-1.8723
35	1	0	-2.603	3.8068	-2.4457
36	1	0	-2.9954	9.485	2.422
37	1	0	-5.2432	8.7884	5.7737
38	1	0	-4.6367	10.386	5.2624
39	1	0	-7.0717	10.3015	4.9047
40	1	0	-6.2826	10.3446	3.2914
41	1	0	-6.9562	8.8003	3.9398
B3LYP/6-311G(d,p) Energy = -1073.2046322 a.u.; Population = 7.23%					

**3a** Conf. 6			Coordinates (Angstroms)		
Center Number	Atomic Number	Atomic Type	X	Y	Z
1	8	0	-0.6384	7.7422	4.4005
2	8	0	-4.5003	6.278	5.2125
3	6	0	-6.2201	5.5799	3.7352
4	6	0	-5.0359	6.4396	4.1399
5	8	0	0.2237	6.4456	2.293
6	8	0	0.8798	5.0436	0.2633
7	6	0	-4.5511	7.487	3.1459
8	6	0	-4.0473	8.8245	3.7186
9	6	0	-2.8049	8.6167	4.5913
10	6	0	-1.7544	7.742	3.9289
11	6	0	-3.7707	6.149	1.1732
12	6	0	-3.4441	6.8602	2.3255
13	6	0	-2.1023	6.964	2.703
14	6	0	-1.09	6.3549	1.9417
15	6	0	-3.1574	4.8387	-0.7433
16	6	0	-2.7809	5.5414	0.403
17	6	0	-1.4359	5.6385	0.7821
18	6	0	-0.4543	5.0147	-0.0111
19	6	0	-0.8554	4.3178	-1.1539
20	6	0	-2.1908	4.222	-1.5292
21	8	0	-3.6121	9.5644	2.5928
22	6	0	-5.1383	9.6866	4.3901
23	6	0	-5.8385	9.1005	5.6222
24	1	0	-5.9683	4.9725	2.8373
25	1	0	-6.5053	4.8815	4.5547
26	1	0	-7.102	6.2206	3.5076
27	1	0	0.3668	6.9198	3.1295
28	1	0	1.07	5.5346	1.0788
29	1	0	-5.4154	7.7314	2.4844
30	1	0	-3.0569	8.164	5.5751

31	1	0	-2.3457	9.6076	4.8179
32	1	0	-4.8288	6.068	0.8735
33	1	0	-4.2163	4.7605	-1.0433
34	1	0	-0.0907	3.827	-1.7792
35	1	0	-2.4789	3.6648	-2.4367
36	1	0	-3.3475	10.4341	2.8518
37	1	0	-4.6807	10.6589	4.6922
38	1	0	-5.9196	9.9312	3.6315
39	1	0	-6.5317	9.8489	6.072
40	1	0	-6.4602	8.2146	5.3638
41	1	0	-5.114	8.811	6.4161
B3LYP/6-311G(d,p) Energy = -1073.2024843 a.u.; Population = 0.74%					

Table S3. Cartesian coordinates for the re-optimized conformers of **4a** at the CAM-B3LYP/6-311G(d,p) level in CH_3OH.

**4a** Conf. 1			Coordinates (Angstroms)		
Center Number	Atomic Number	Atomic Type	X	Y	Z
1	6	0	-1.967913	-2.689036	0.913626
2	8	0	2.003059	3.243205	0.151611
3	8	0	5.329664	-0.675095	-1.841528
4	6	0	0.252411	-2.019911	0.035816
5	6	0	-0.939845	-1.605027	0.664008
6	6	0	-1.107491	-0.239305	1.028614
7	6	0	-0.061974	0.679718	0.747993
8	6	0	2.579351	-1.321176	-0.845358
9	6	0	1.259722	-1.105107	-0.231161
10	6	0	1.100091	0.248042	0.108993
11	6	0	4.5456	0.179897	-1.436247
12	6	0	3.195665	-0.13071	-0.915168
13	6	0	2.342411	0.90766	-0.346953
14	6	0	2.743916	2.192258	-0.332276
15	6	0	4.071072	2.571113	-0.877459
16	6	0	4.490574	3.913836	-0.867557
17	6	0	5.738124	4.287154	-1.375165
18	6	0	6.591593	3.324139	-1.90372
19	6	0	6.197243	1.990595	-1.924094
20	6	0	4.94488	1.606844	-1.415706
21	6	0	-2.70383	-3.085817	-0.359157
22	16	0	3.116196	-2.850832	-1.371873
23	6	0	3.630331	-3.642183	0.200864
24	6	0	4.873725	-3.062676	0.895211
25	7	0	5.962316	-2.788623	-0.042768
26	6	0	7.271628	-2.829743	0.372252
27	6	0	8.250959	-2.301213	-0.640098
28	8	0	7.619526	-3.242928	1.473647

29	6	0	-2.056527	4.575797	3.08004
30	6	0	-0.916775	3.803605	2.428826
31	8	0	-1.944919	4.075896	-0.220544
32	8	0	-5.014812	-1.619933	1.560432
33	8	0	-6.659798	1.596759	-2.481104
34	8	0	-8.605074	-0.010029	-3.022483
35	6	0	-2.212578	2.87633	0.409885
36	6	0	-1.334349	2.539877	1.631194
37	6	0	-2.022055	1.504569	2.566444
38	6	0	-2.383073	0.278916	1.707214
39	6	0	-4.22551	2.207599	-0.969335
40	6	0	-3.194209	2.040652	0.031731
41	6	0	-3.393442	0.746271	0.690728
42	6	0	-6.251176	0.852757	-1.596378
43	6	0	-5.039379	1.149188	-0.826
44	6	0	-4.558201	0.270629	0.217068
45	6	0	-5.323875	-0.922553	0.60139
46	6	0	-6.523005	-1.245932	-0.215652
47	8	0	-0.092698	1.998033	1.117516
48	6	0	-7.238055	-2.423292	0.062058
49	6	0	-8.364599	-2.767508	-0.681367
50	6	0	-8.797568	-1.936572	-1.71088
51	6	0	-8.102584	-0.763641	-1.995307
52	6	0	-6.962004	-0.405816	-1.257341
53	8	0	7.095231	1.107178	-2.462957
54	6	0	4.487383	-1.858088	1.761306
55	8	0	3.588159	-1.83685	2.589945
56	8	0	5.226737	-0.747547	1.555311
57	8	0	-1.219153	1.140037	3.697361
58	8	0	-6.892814	-3.300818	1.055381
59	1	0	-1.457514	-3.571179	1.322661
60	1	0	-2.677787	-2.401873	1.688246
61	1	0	1.134389	2.904867	0.439478

62	1	0	0.38114	-3.065524	-0.236915
63	1	0	3.844236	4.690298	-0.460658
64	1	0	6.041377	5.331284	-1.357
65	1	0	7.562601	3.610212	-2.299736
66	1	0	-3.490911	-3.811321	-0.130402
67	1	0	-3.162904	-2.221319	-0.84709
68	1	0	-2.024404	-3.548563	-1.082981
69	1	0	2.772933	-3.676587	0.881615
70	1	0	3.850973	-4.68177	-0.069056
71	1	0	5.238281	-3.810807	1.61137
72	1	0	5.746022	-2.158714	-0.817543
73	1	0	9.172096	-2.8884	-0.59084
74	1	0	7.851618	-2.378649	-1.655395
75	1	0	8.468543	-1.253722	-0.416208
76	1	0	-1.666185	5.482712	3.553678
77	1	0	-2.810647	4.881225	2.349218
78	1	0	-2.546939	3.986776	3.860106
79	1	0	-0.191454	3.522281	3.203889
80	1	0	-0.35942	4.485161	1.771987
81	1	0	-2.495502	4.157485	-1.017799
82	1	0	-8.054067	0.797536	-3.130249
83	1	0	-2.945048	1.924316	2.986847
84	1	0	-2.792279	-0.479956	2.378166
85	1	0	-4.347951	3.020503	-1.6664
86	1	0	-8.906637	-3.683209	-0.45586
87	1	0	-9.67827	-2.201798	-2.291528
88	1	0	6.699283	0.209246	-2.425836
89	1	0	4.933374	-0.124887	2.253021
90	1	0	-0.34612	0.852634	3.372412
91	1	0	-6.12303	-2.940673	1.550156
CAM-B3LYP/6-311G(d,p) Energy =-3088.9031236 a.u.; Population = 100%					

Table S4. Imaginary frequencies and absolute energy values of the re-optimized conformers of **3a** at the B3LYP/6-311G(d,p) level in CH_3OH.

**3a** Conf.1	Imaginary Freq = 0
	Zero-point correction=0.326381 (Hartree/Particle)<br>Thermal correction to Energy=0.346863<br>Thermal correction to Enthalpy=0.347807<br>Thermal correction to Gibbs Free Energy=0.277960<br>Sum of electronic and zero-point Energies=-1072.879131<br>Sum of electronic and thermal Energies=-1072.858649<br>Sum of electronic and thermal Enthalpies=-1072.857705<br>Sum of electronic and thermal Free Energies=-1072.927551
**3a** Conf.2	Imaginary Freq = 0
	Zero-point correction= 0.326210 (Hartree/Particle)<br>Thermal correction to Energy= 0.346824<br>Thermal correction to Enthalpy= 0.347768<br>Thermal correction to Gibbs Free Energy= 0.277181<br>Sum of electronic and zero-point Energies= -1072.879119<br>Sum of electronic and thermal Energies= -1072.858506<br>Sum of electronic and thermal Enthalpies= -1072.857562<br>Sum of electronic and thermal Free Energies= -1072.928149
**3a** Conf.3	Imaginary Freq = 0
	Zero-point correction=0.326461 (Hartree/Particle)<br>Thermal correction to Energy= 0.346951<br>Thermal correction to Enthalpy= 0.347895<br>Thermal correction to Gibbs Free Energy= 0.277636<br>Sum of electronic and zero-point Energies= -1072.879853<br>Sum of electronic and thermal Energies= -1072.859364<br>Sum of electronic and thermal Enthalpies= -1072.858420<br>Sum of electronic and thermal Free Energies= -1072.928679
**3a** Conf.4	Imaginary Freq = 0

	Zero-point correction=0.326424 (Hartree/Particle)<br>Thermal correction to Energy= 0.346909<br>Thermal correction to Enthalpy= 0.347853<br>Thermal correction to Gibbs Free Energy= 0.278003<br>Sum of electronic and zero-point Energies= -1072.878925<br>Sum of electronic and thermal Energies= -1072.858440<br>Sum of electronic and thermal Enthalpies= -1072.857496<br>Sum of electronic and thermal Free Energies= -1072.927347
**3a** Conf.5	Imaginary Freq = 0
	Zero-point correction=0.326343 (Hartree/Particle)<br>Thermal correction to Energy=0.346839<br>Thermal correction to Enthalpy=0.347783<br>Thermal correction to Gibbs Free Energy=0.277849<br>Sum of electronic and zero-point Energies= -1072.878289<br>Sum of electronic and thermal Energies= -1072.857793<br>Sum of electronic and thermal Enthalpies= -1072.856849<br>Sum of electronic and thermal Free Energies= -1072.926783
**3a** Conf.6	Imaginary Freq = 0
	Zero-point correction= 0.326431 (Hartree/Particle)<br>Thermal correction to Energy= 0.346885<br>Thermal correction to Enthalpy= 0.347829<br>Thermal correction to Gibbs Free Energy= 0.278212<br>Sum of electronic and zero-point Energies= -1072.876054<br>Sum of electronic and thermal Energies= -1072.855599<br>Sum of electronic and thermal Enthalpies= -1072.854655<br>Sum of electronic and thermal Free Energies= -1072.924272

Table S5. Imaginary frequencies and absolute energy values of the re-optimized conformers of **4a** at the CAM-B3LYP/6-311G(d,p) level in CH_3OH.

**4a** Conf.1	Imaginary Freq = 0
	Zero-point correction= 0.690266 (Hartree/Particle)<br>Thermal correction to Energy= 0.740225<br>Thermal correction to Enthalpy= 0.741169<br>Thermal correction to Gibbs Free Energy= 0.603607<br>Sum of electronic and zero-point Energies= -3088.772635<br>Sum of electronic and thermal Energies= -3088.722676<br>Sum of electronic and thermal Enthalpies= -3088.721732<br>Sum of electronic and thermal Free Energies= -3088.859294

Figure S1. HPLC profile of the crude extracts from *M. echinospora* SCSIO 04089 and Δ*nes5* mutant fermented on N4 medium with HP20 resin.

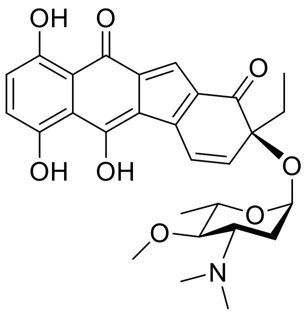

NEN A

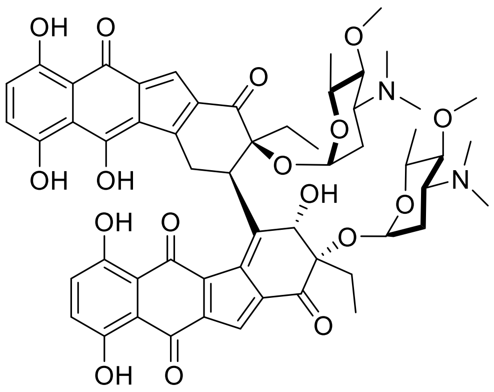

NEN B

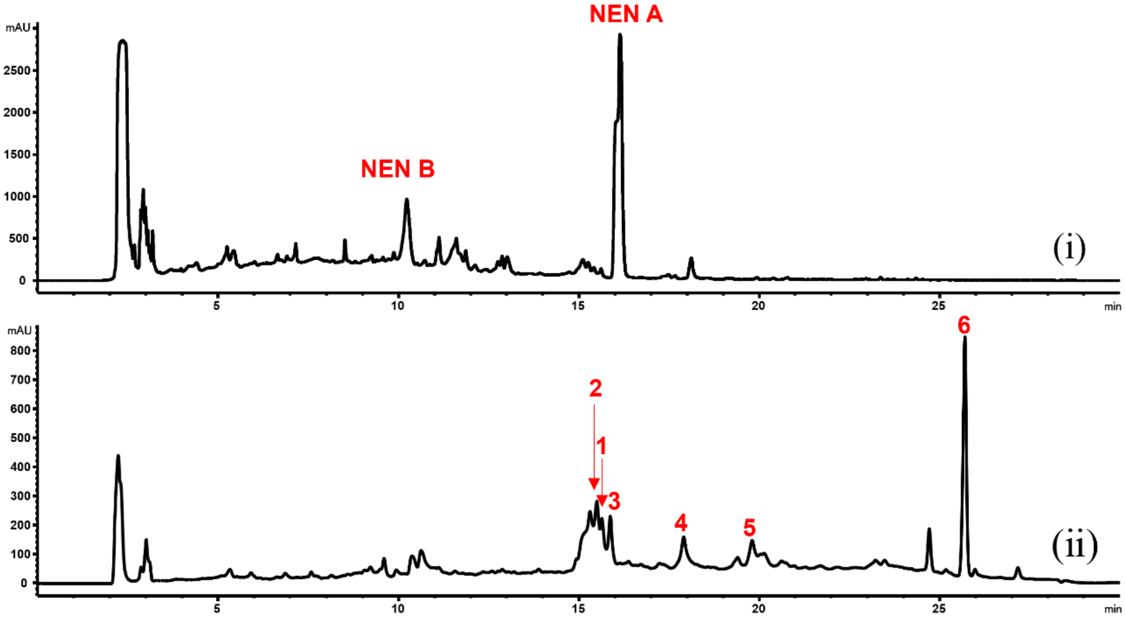

(i) *M. echinospora* SCSIO 04089; (ii) Δ*nes5* mutant

For HPLC analysis, the HP20 resins obtained from 50 mL fermentations of *M. echinospora* SCSIO 04089 and the Δ*nes5* mutant were eluted with 10 mL of CH_3OH, respectively. After drying under vacuum condition, the residue was dissolved in 1 mL of CH_3OH. A 50 μL aliquot of this solution was injected for analysis. HPLC analysis was carried out using a reversed phase column (Phenomenex Luna C18, 150 x 4.6 mm, 5 μm) with UV detection at 254 nm under the following program: solvent system (solvent A, 0.1% formic acid in H_2O; solvent B, 100% CH_3CN); 5% B to 100% B (0 – 20 min), 100% B (20 – 24 min), 100% B to 5% B (24 – 25 min), 5% B (25 – 30 min); flow rate at 1 mL min^{-1}.

Figure S2. The spectroscopic data of NEN E (**1**).

(A) The HRESIMS spectrum of **1**

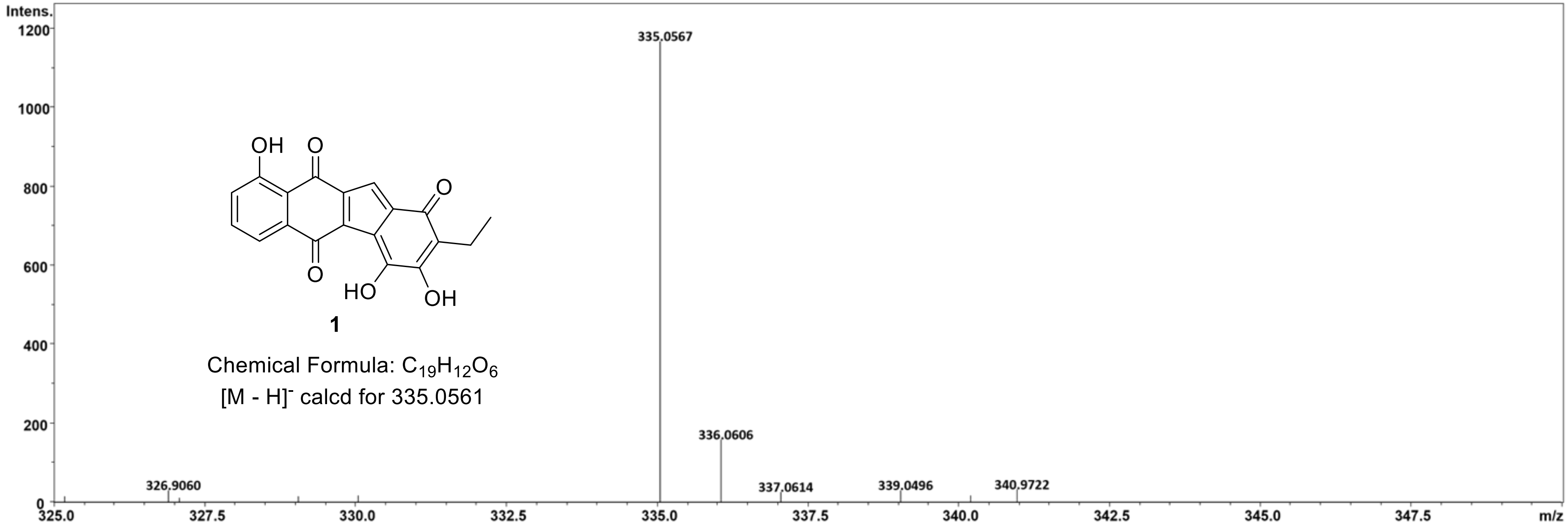

Figure S2. The spectroscopic data of NEN E (**1**).

(B) The ^{1}H-NMR spectrum of **1** (700 MHz for ^{1}H NMR in DMSO-d_6)

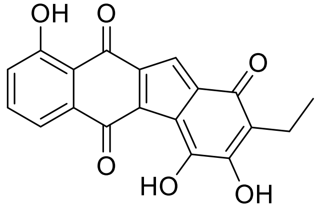

1

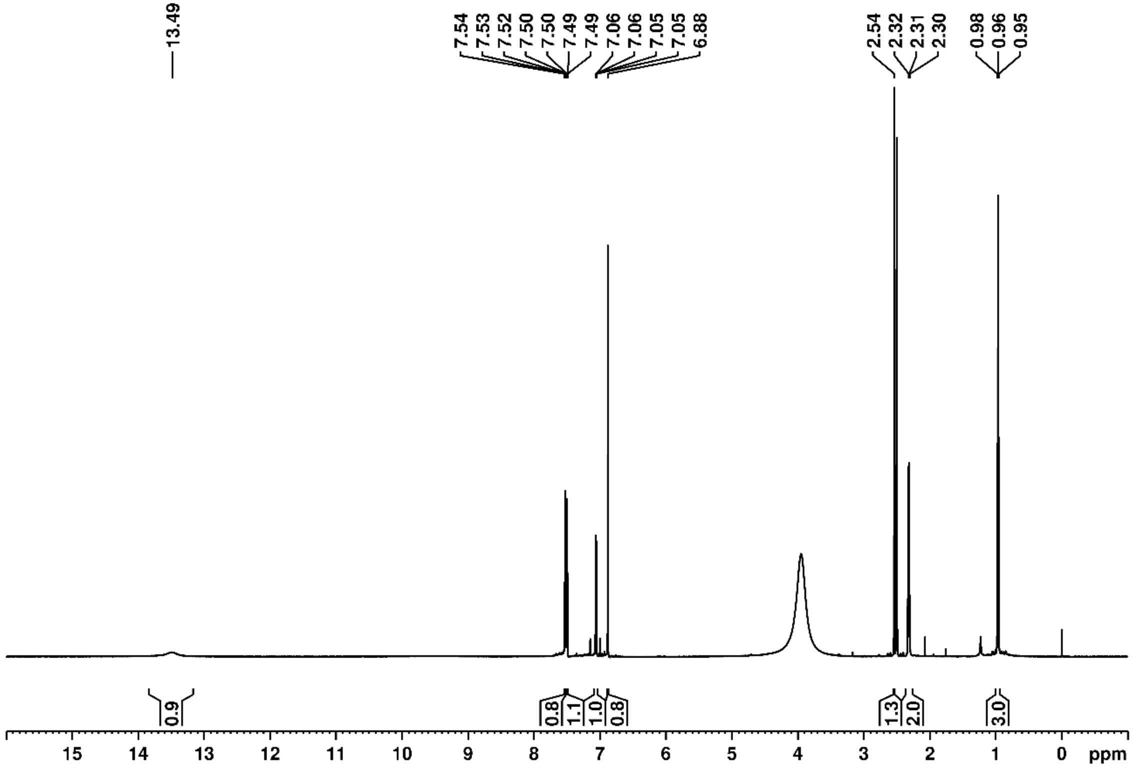

Figure S2. The spectroscopic data of NEN E (**1**).

(C) The ^{13}C-NMR spectrum of **1** (175 MHz for ^{13}C NMR in DMSO-d_6)

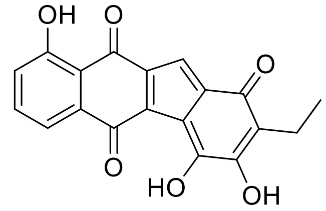

1

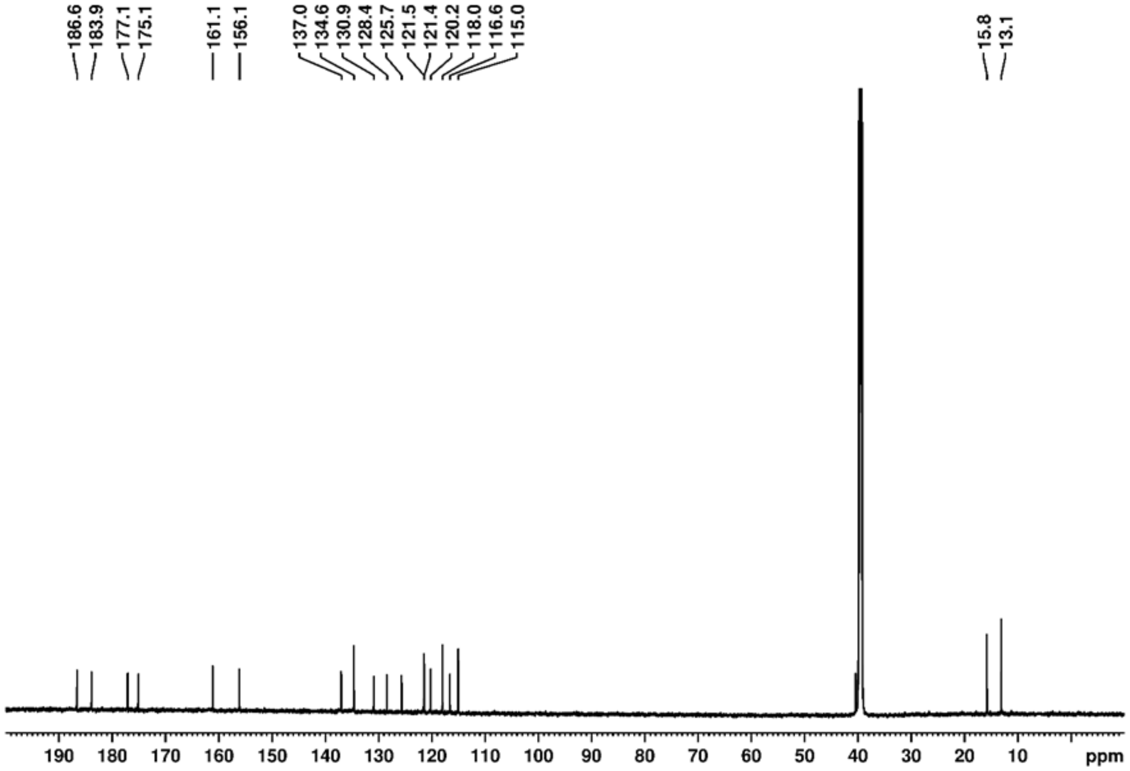

Figure S2. The spectroscopic data of NEN E (**1**).

(D) The ^{1}H-^{1}H COSY spectrum of **1**

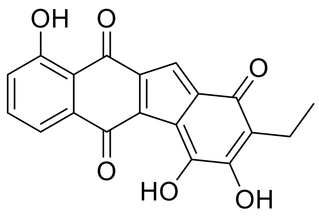

1

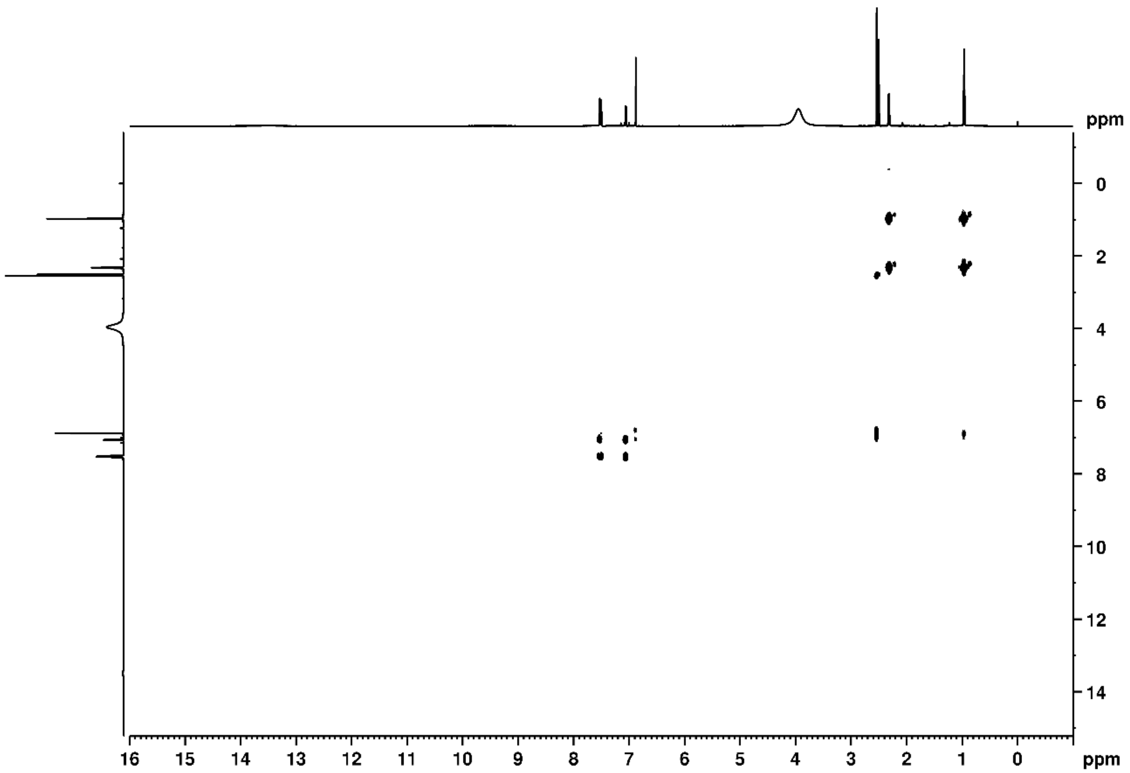

Figure S2. The spectroscopic data of NEN E (**1**).

(E) The HSQC spectrum of **1**

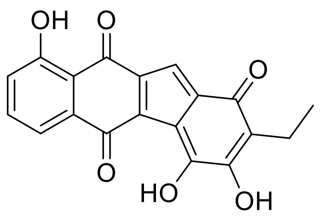

1

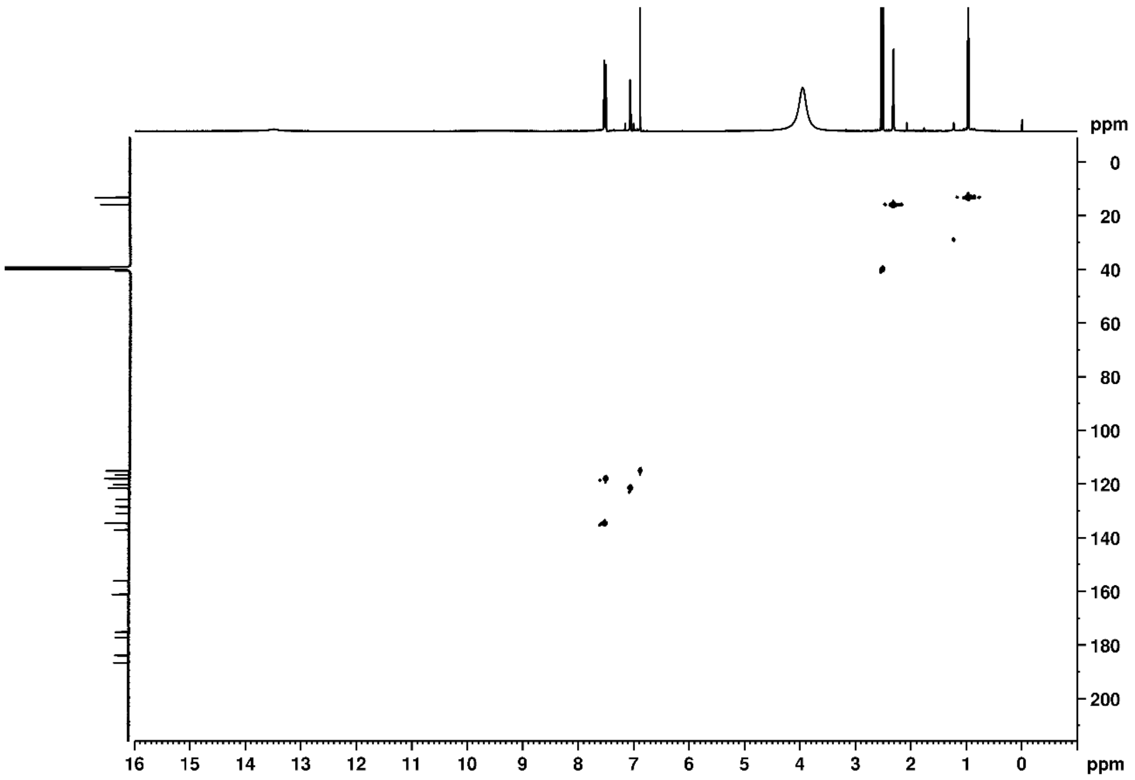

Figure S2. The spectroscopic data of NEN E (**1**).

(F) The HMBC spectrum of **1**

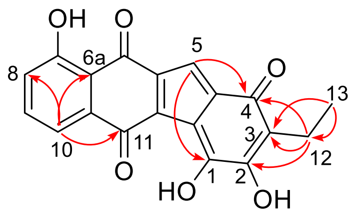

1

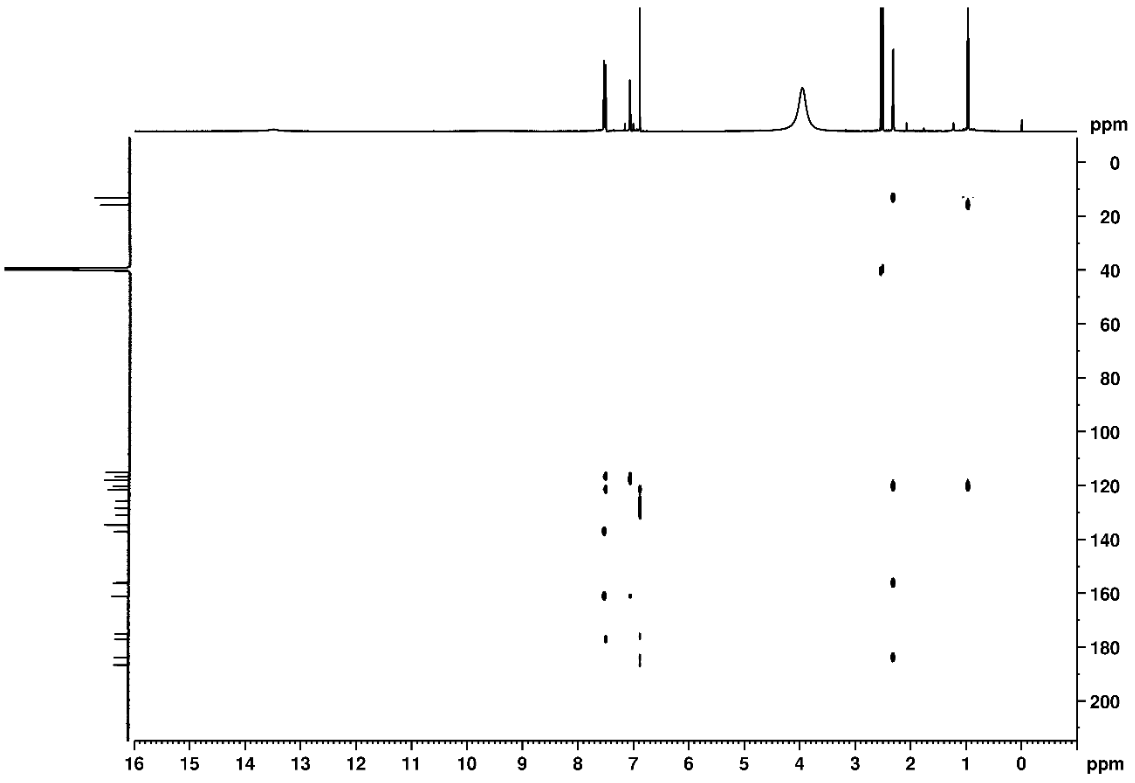

Figure S2. The spectroscopic data of NEN E (**1**).

(G) The NOESY spectrum of **1**

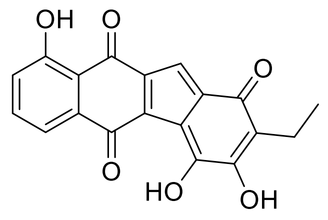

1

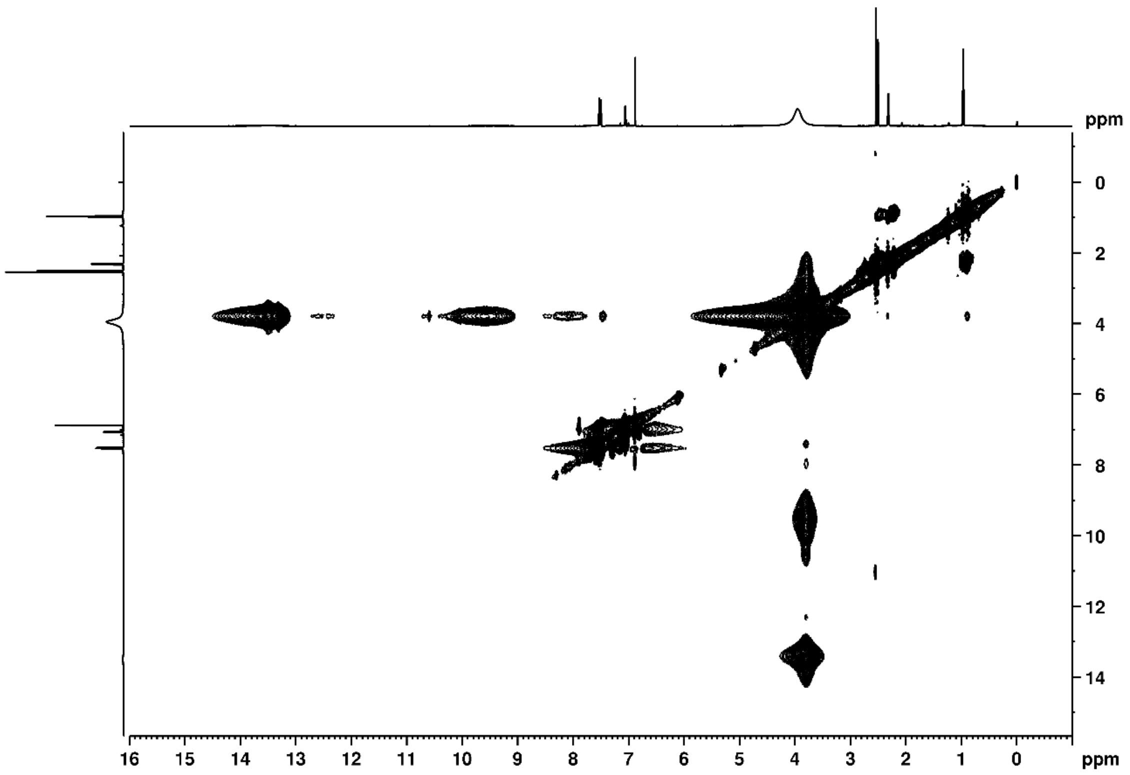

Figure S2. The spectroscopic data of NEN E (**1**).

(H) The UV spectrum of **1**

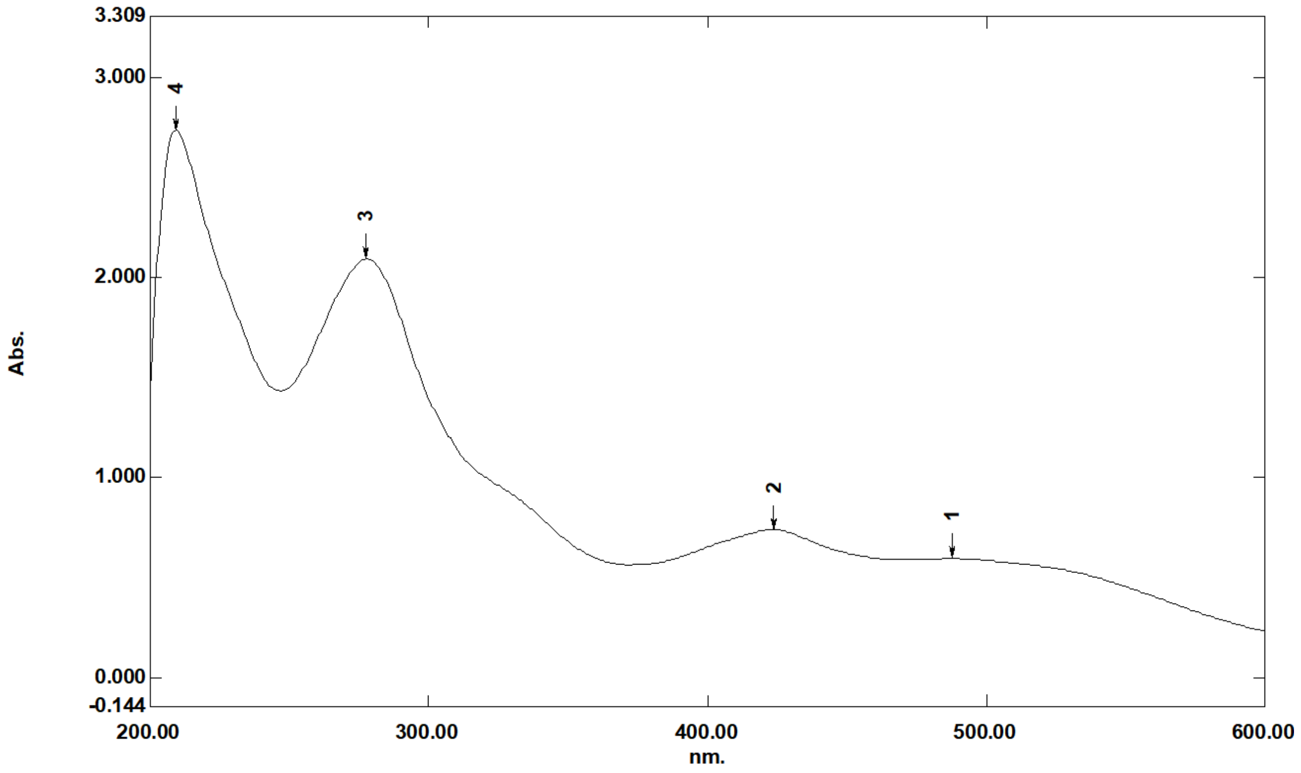

Figure S2. The spectroscopic data of NEN E (**1**).

(I) The IR spectrum of **1**

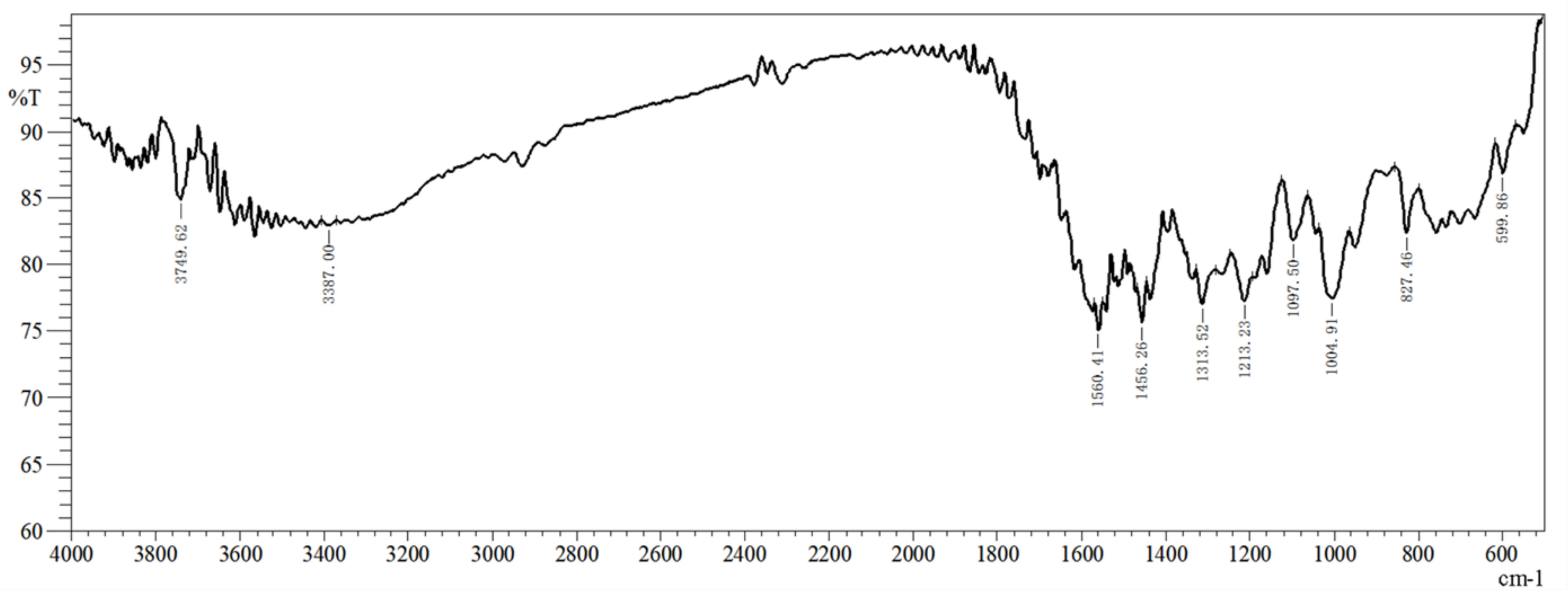

Figure S3. The spectroscopic data of NEN F (**2**).

(A) The HRESIMS spectrum of **2**

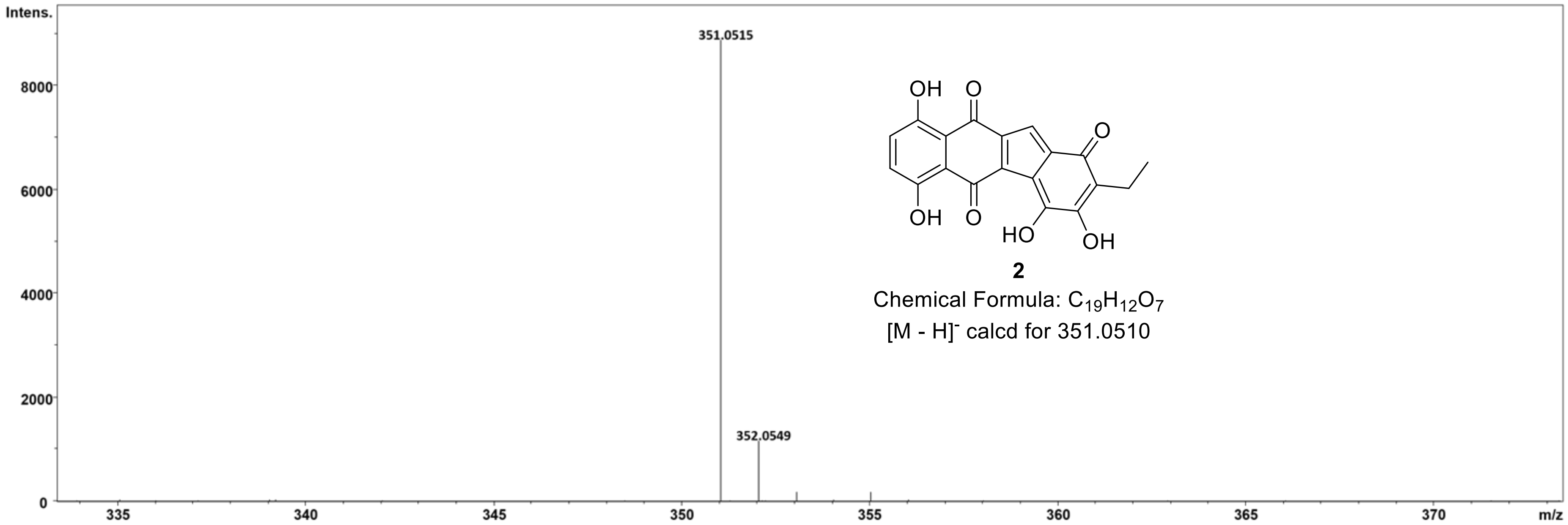

Figure S3. The spectroscopic data of NEN F (**2**).

(B) The ^{1}H-NMR spectrum of **2** (700 MHz for ^{1}H NMR in DMSO-d_6)

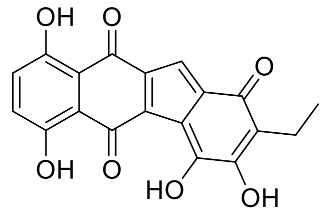

2

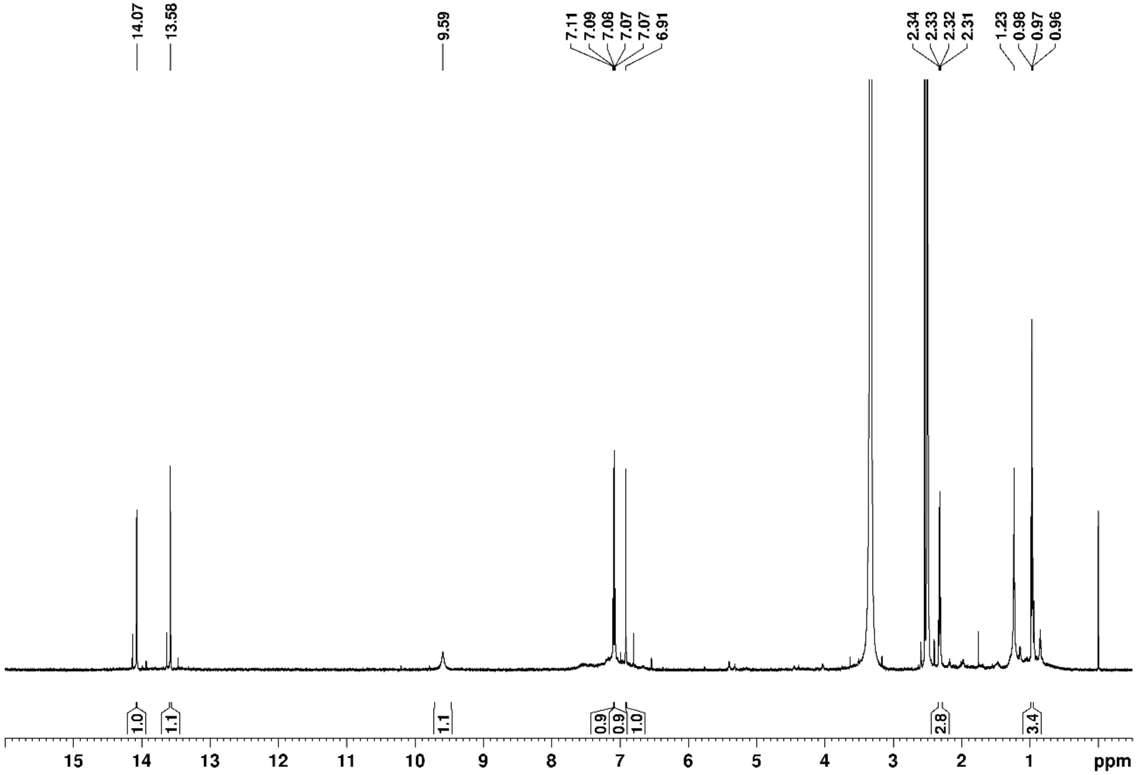

Figure S3. The spectroscopic data of NEN F (**2**).

(C) The ^{13}C-NMR spectrum of **2** (175 MHz for ^{13}C NMR in DMSO-d_6)

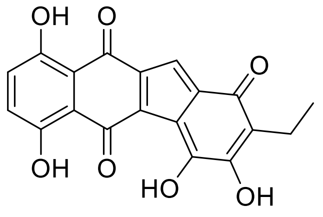

2

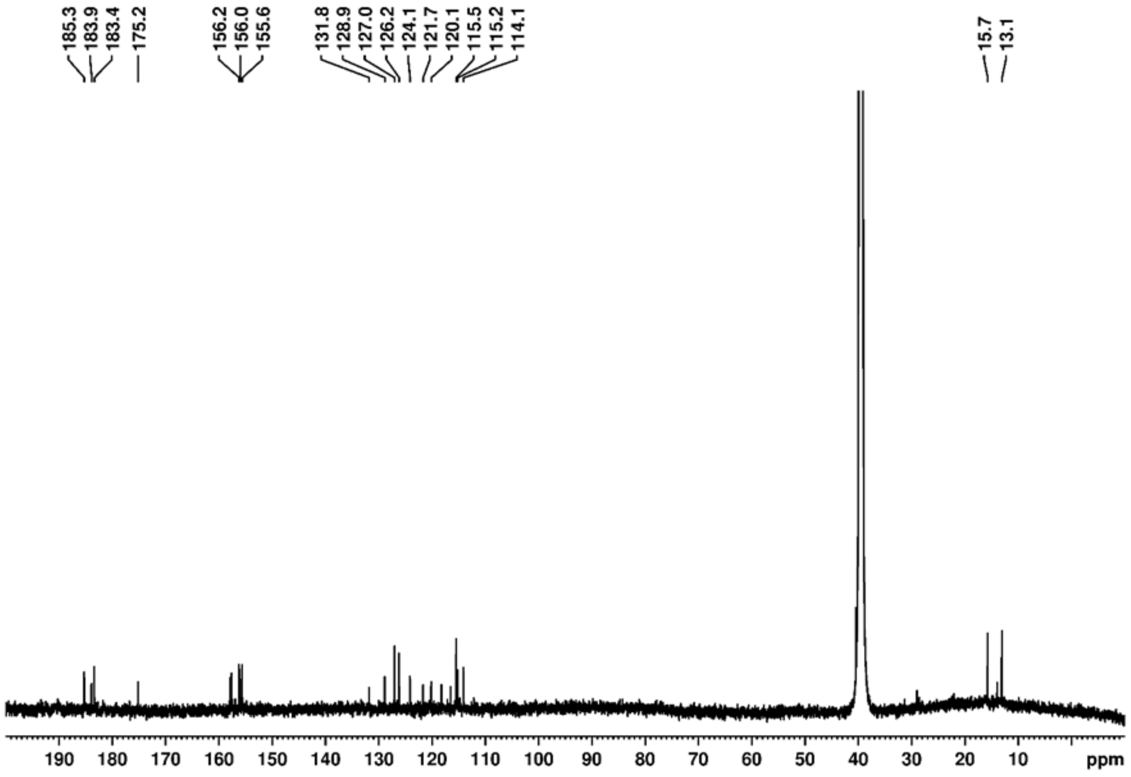

Figure S3. The spectroscopic data of NEN F (**2**).

(D) The ^{1}H-^{1}H COSY spectrum of **2**

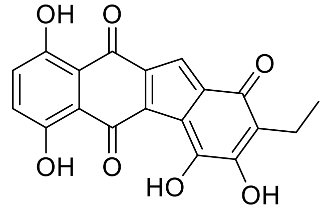

2

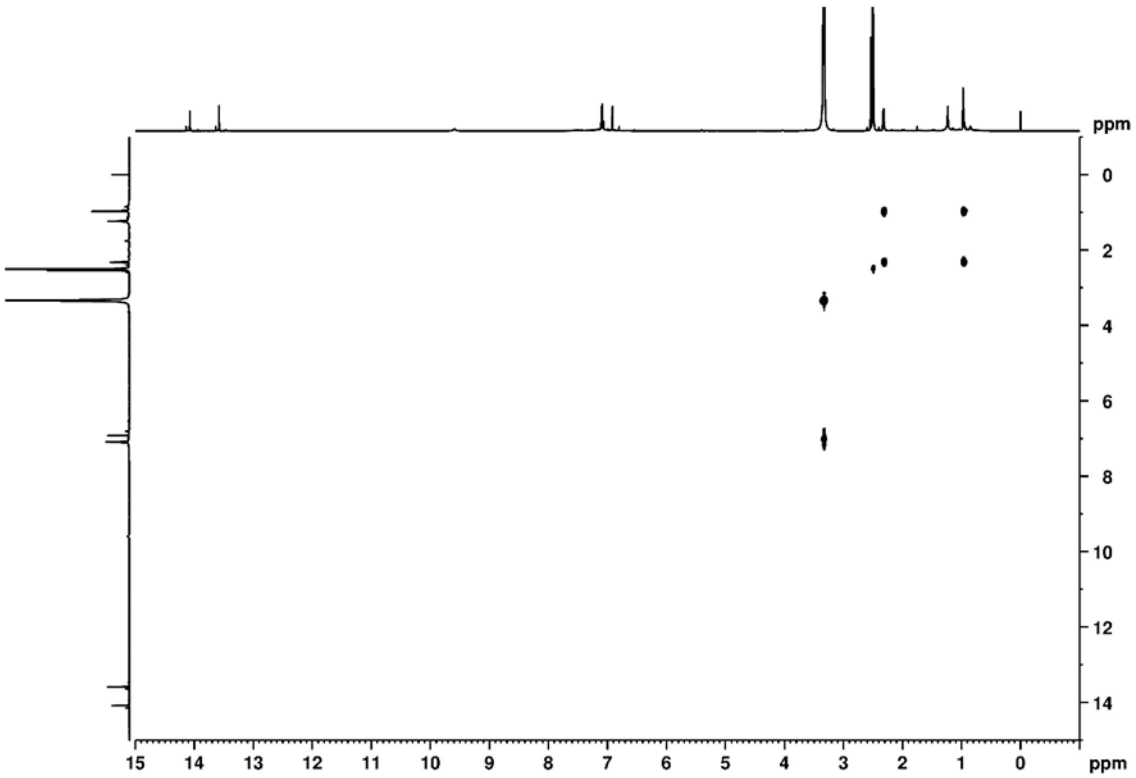

Figure S3. The spectroscopic data of NEN F (**2**).

(E) The HSQC spectrum of **2**

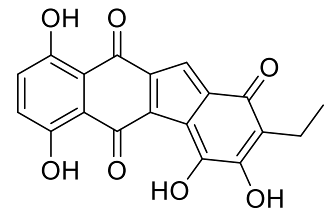

2

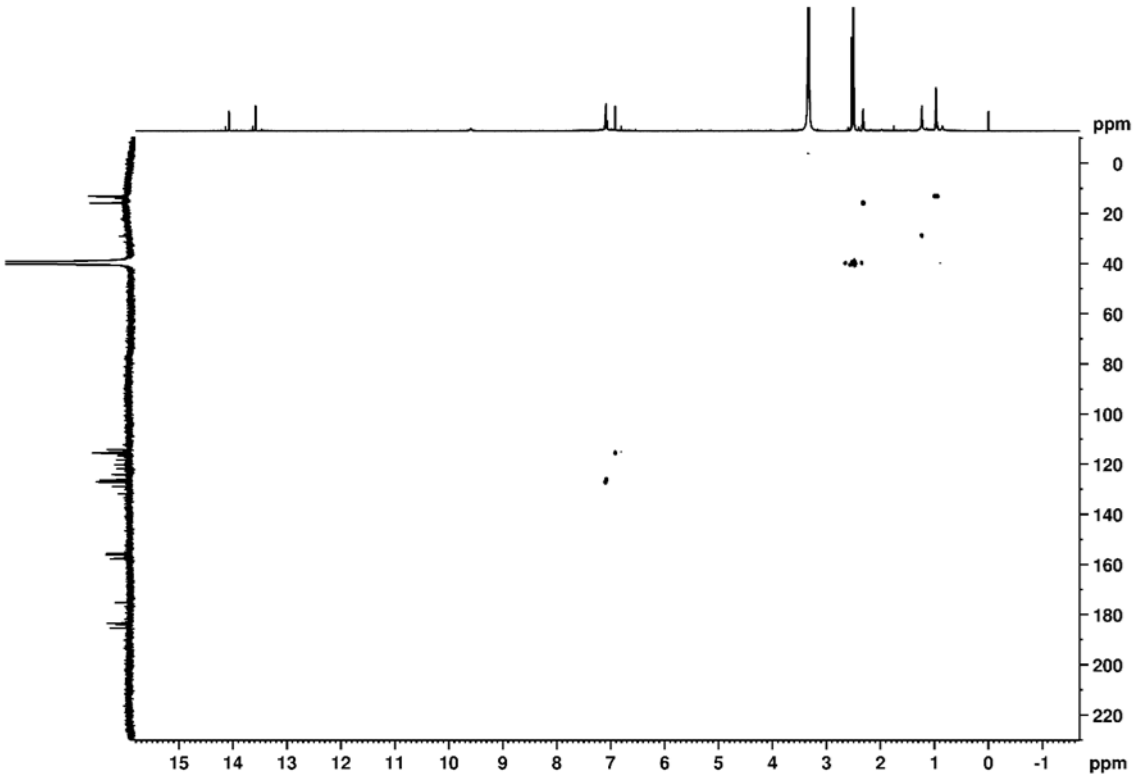

Figure S3. The spectroscopic data of NEN F (**2**).

(F) The HMBC spectrum of **2**

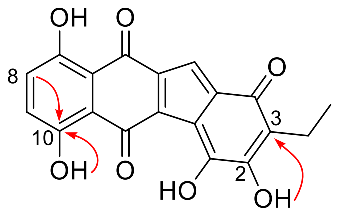

2

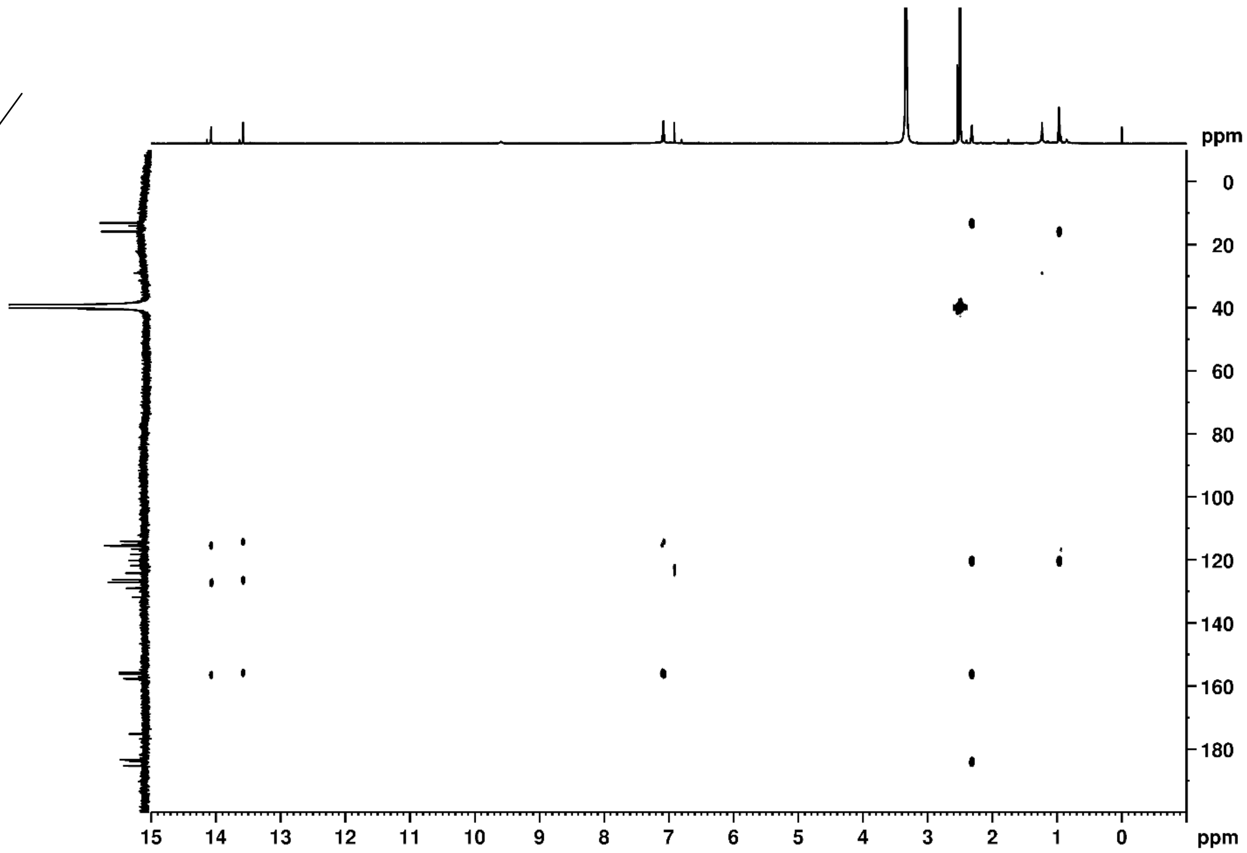

Figure S3. The spectroscopic data of NEN F (**2**).

(G) The HMBC spectrum of **2** (enlarged figure)

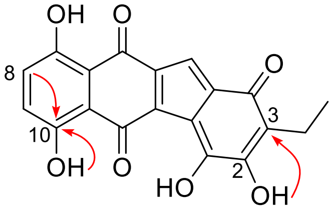

2

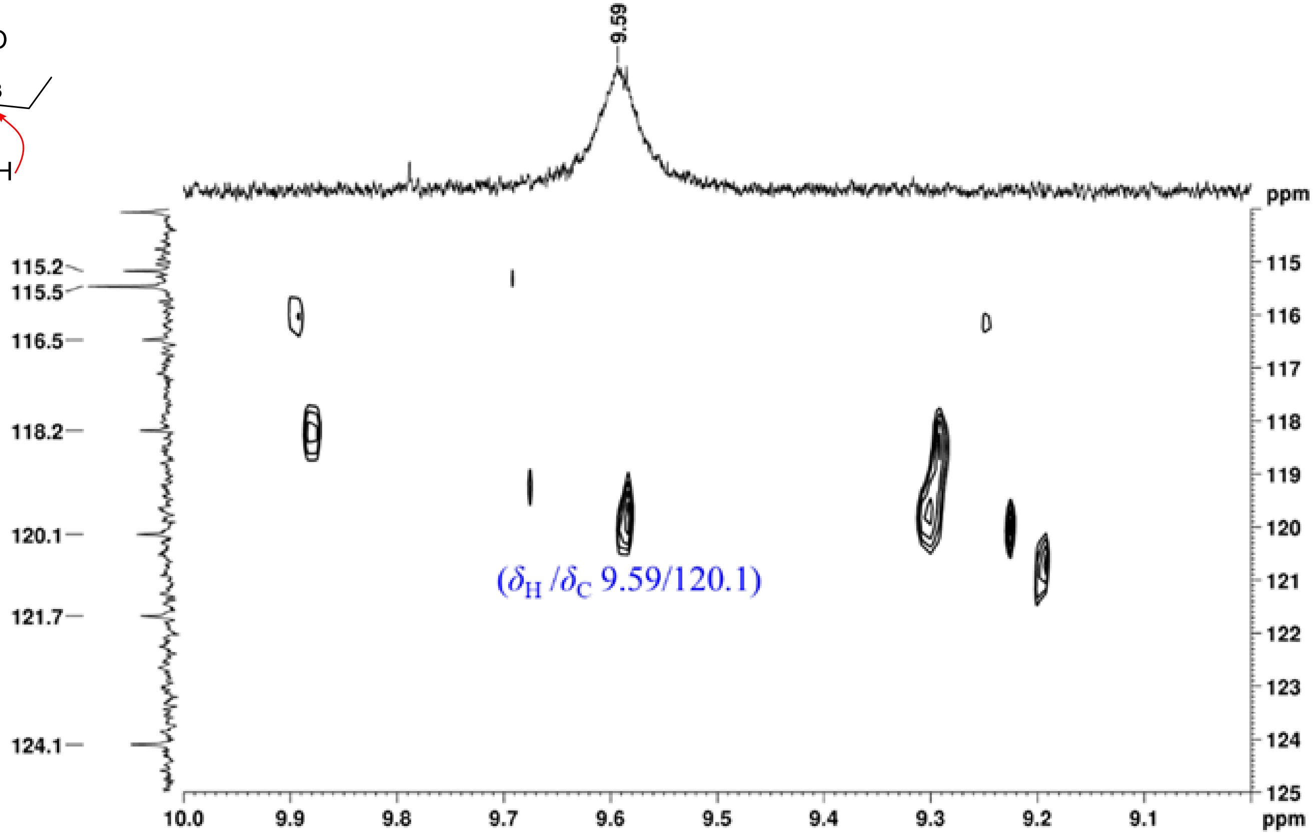

Figure S3. The spectroscopic data of NEN F (**2**).

(H) The NOESY spectrum of **2**

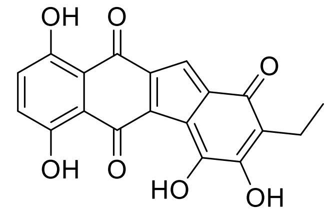

2

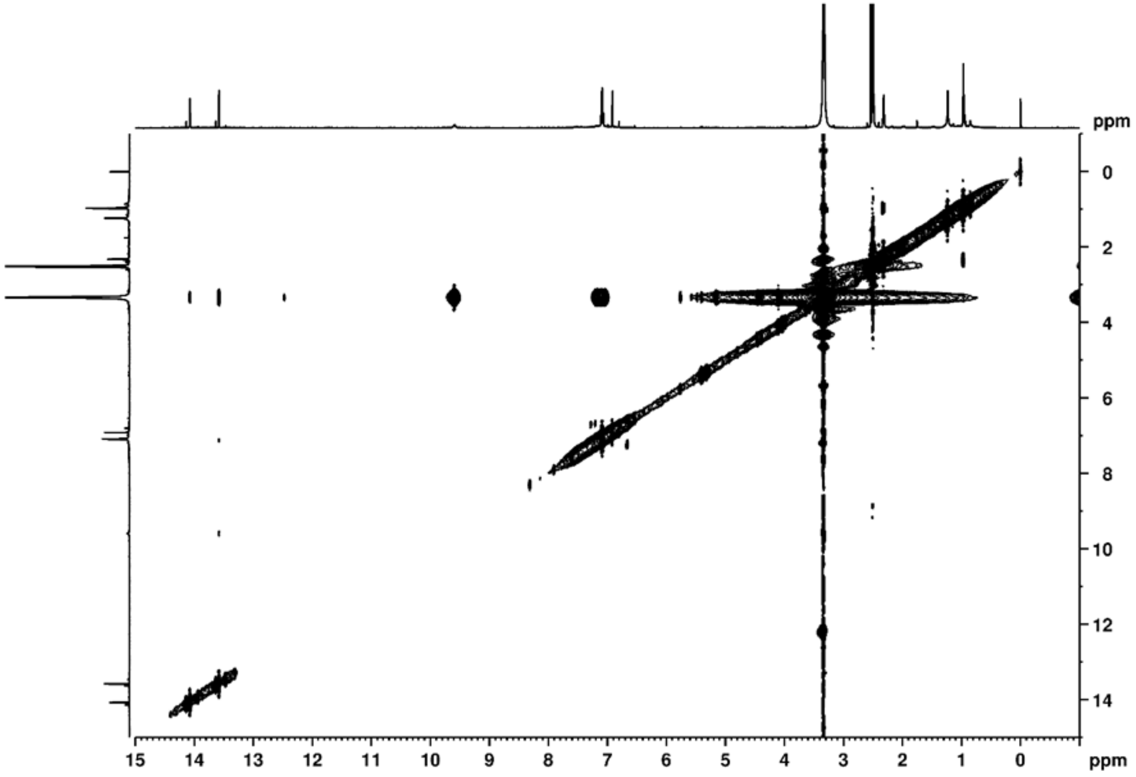

Figure S3. The spectroscopic data of NEN F (**2**).

(I) The UV spectrum of **2**

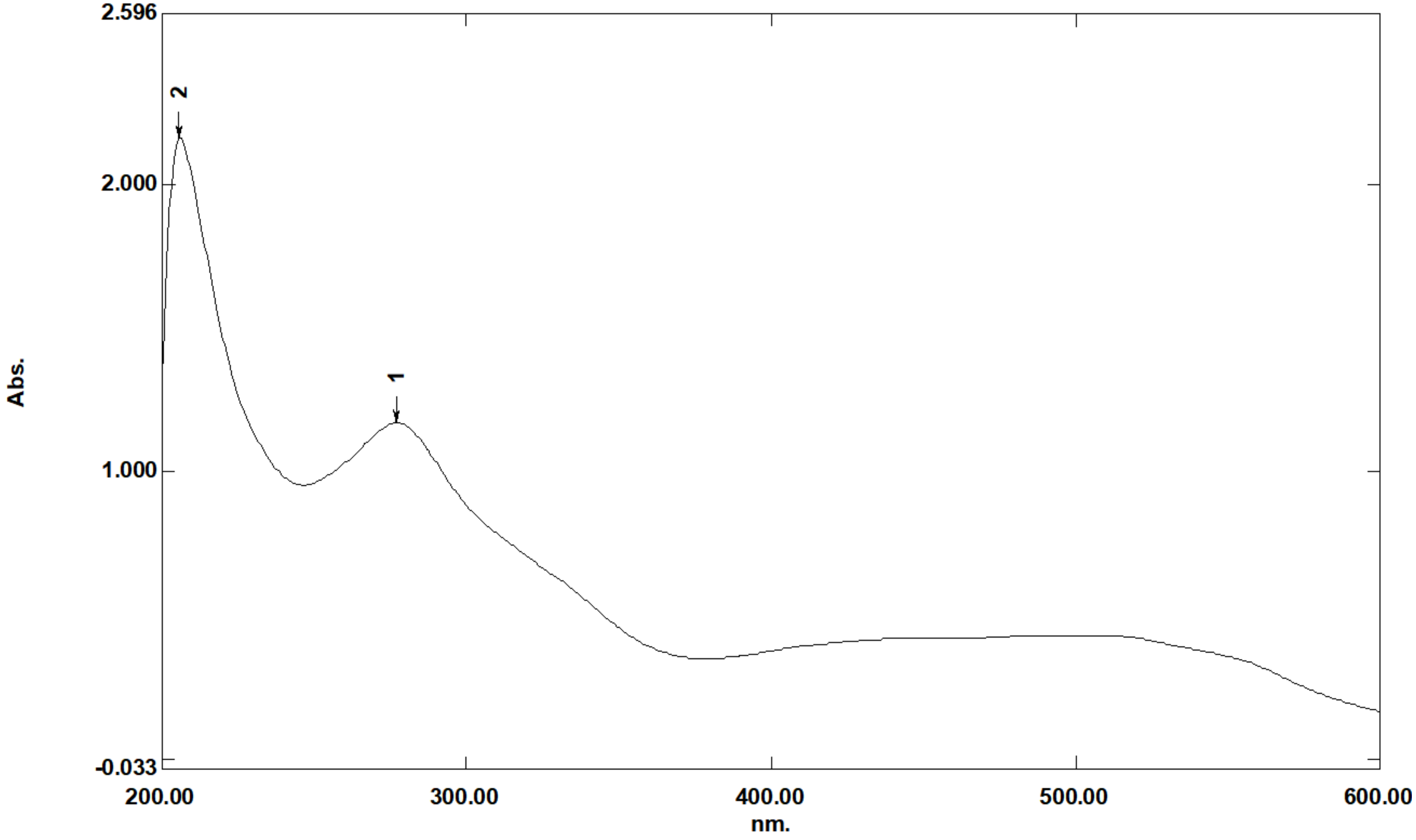

Figure S3. The spectroscopic data of NEN F (**2**).

(J) The IR spectrum of **2**

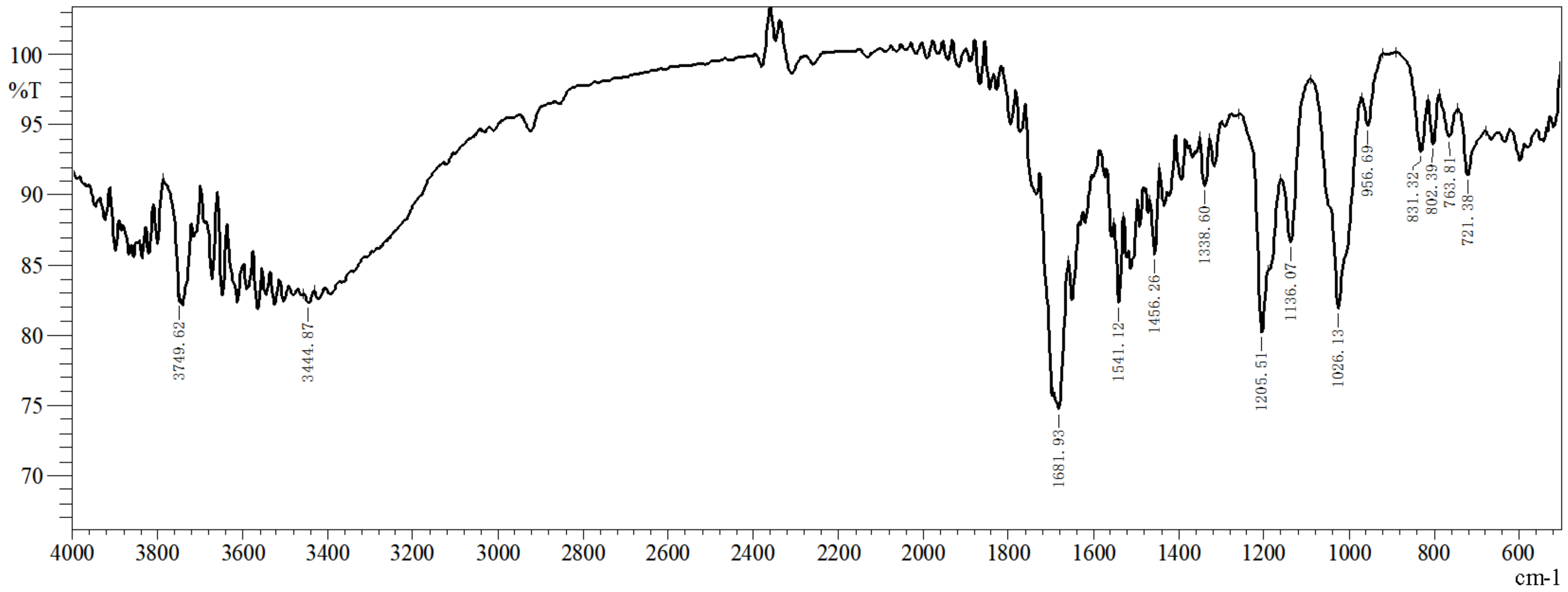

Figure S4. The spectroscopic data of NEN G (**3**).

(A) The HRESIMS spectrum of **3**

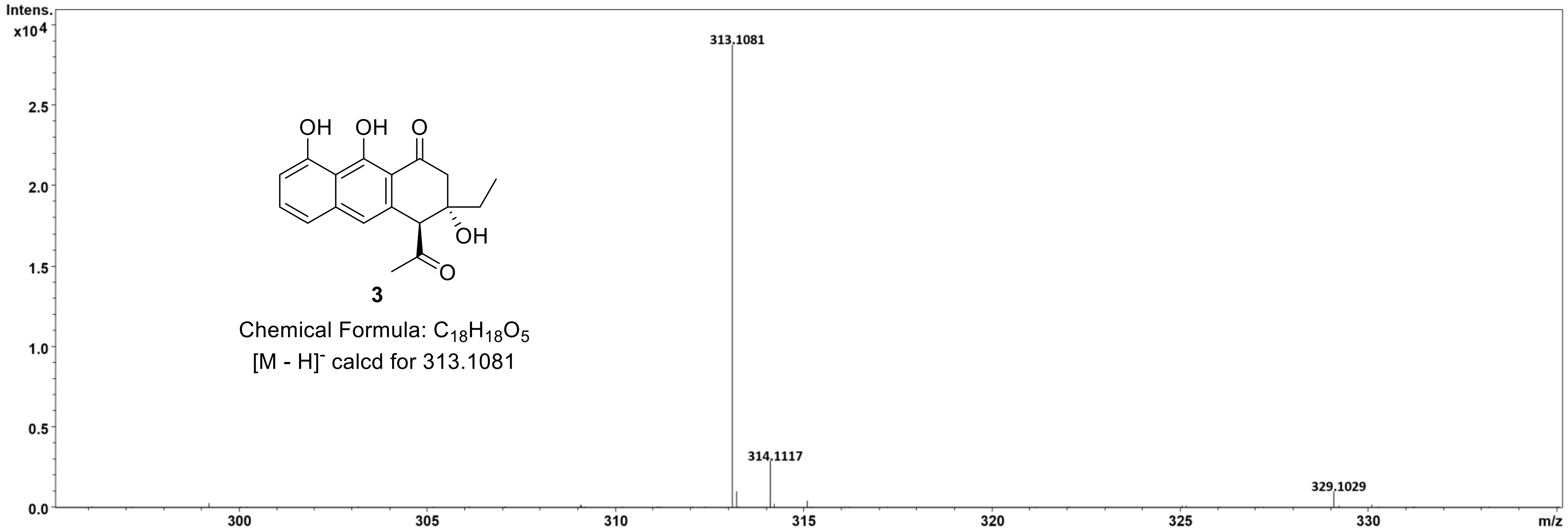

Figure S4. The spectroscopic data of NEN G (**3**).

(B) The ^{1}H-NMR spectrum of **3** (700 MHz for ^{1}H NMR in DMSO-d_6)

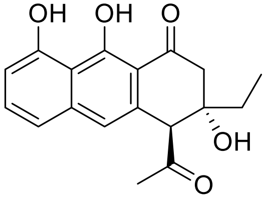

3

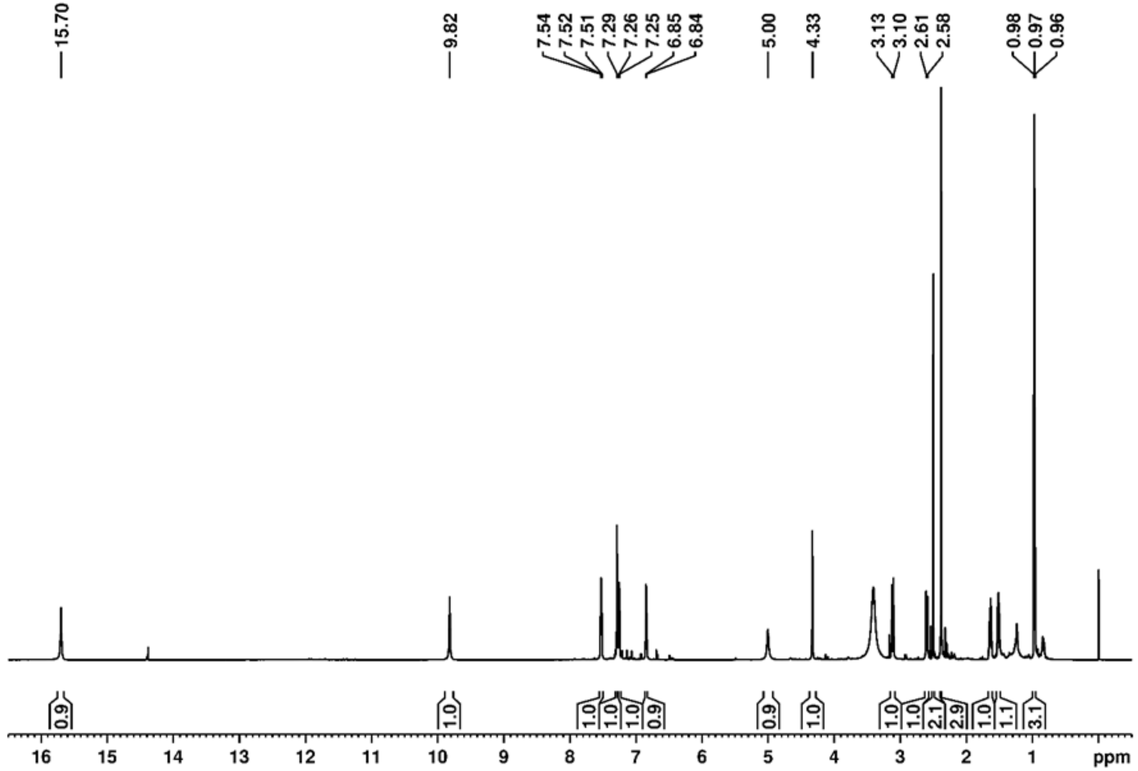

Figure S4. The spectroscopic data of NEN G (**3**).

(C) The ^{13}C-NMR spectrum of **3** (175 MHz for ^{13}C NMR in DMSO-d_6)

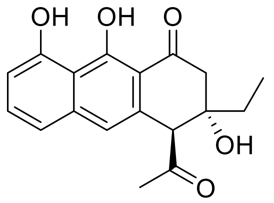

3

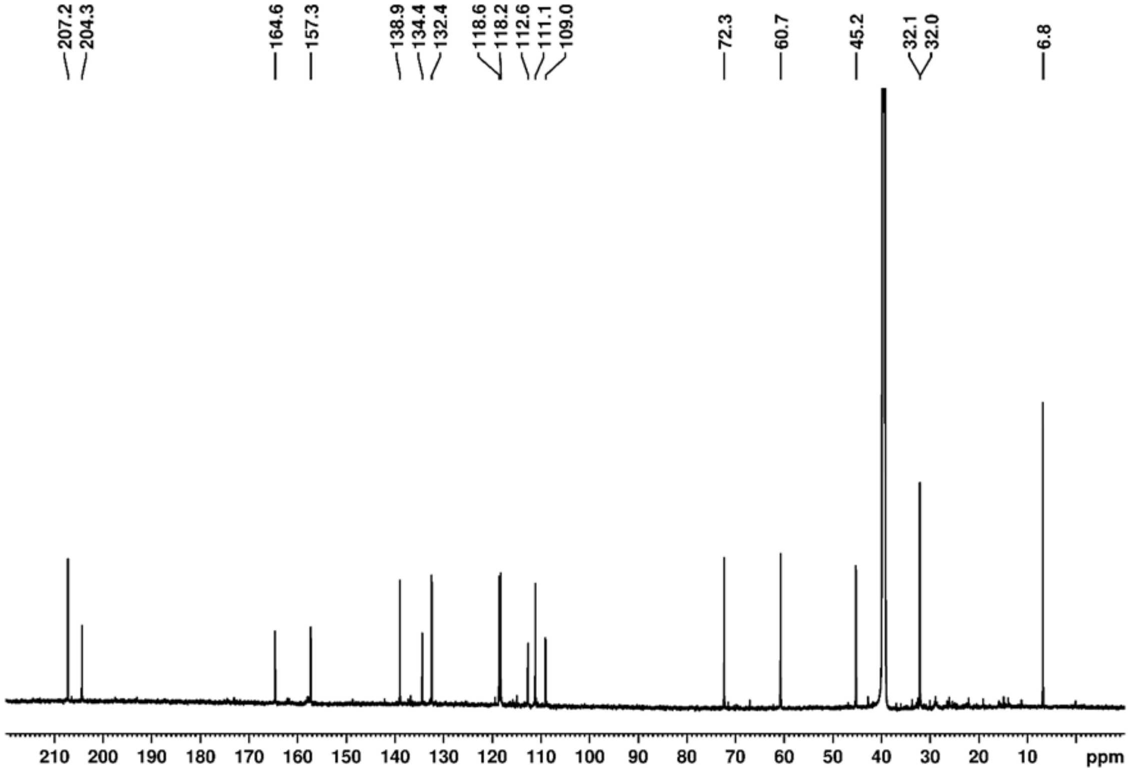

Figure S4. The spectroscopic data of NEN G (**3**).

(D) The HSQC spectrum of **3** (in DMSO-*d*$_6$)

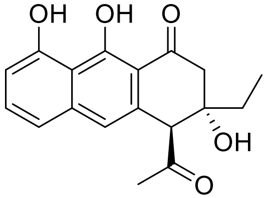

3

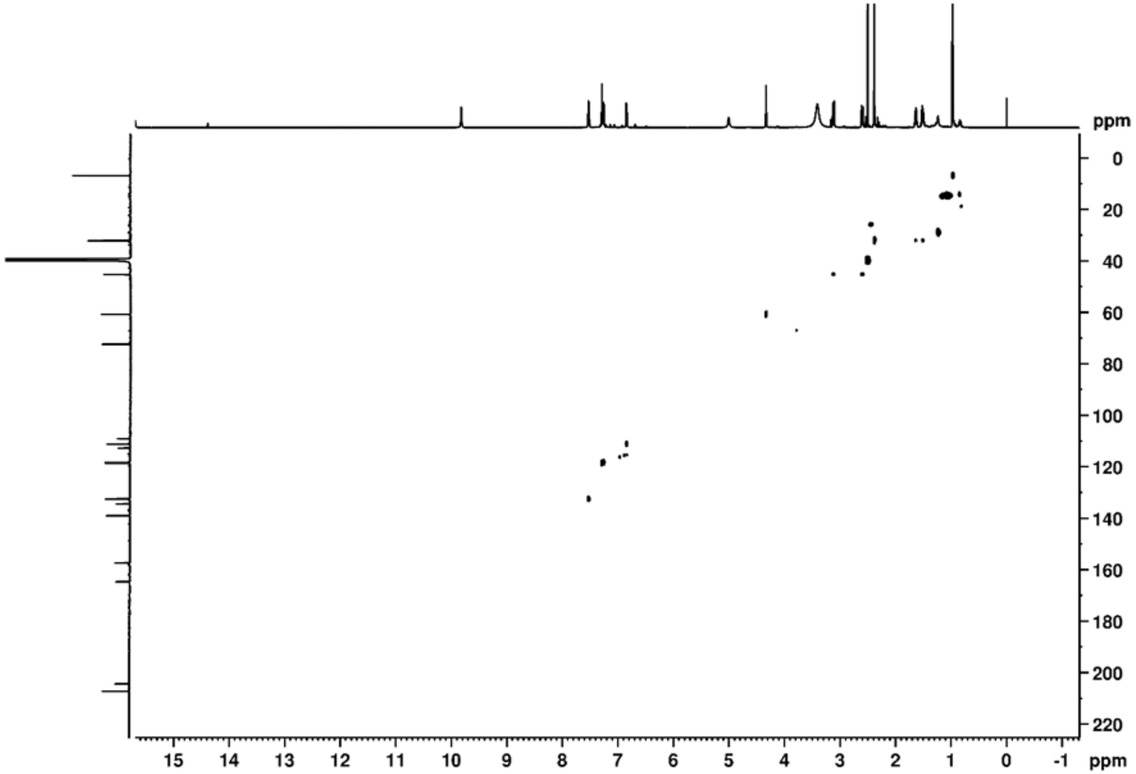

Figure S4. The spectroscopic data of NEN G (**3**).

(E) The HMBC spectrum of **3** (in DMSO-d_6)

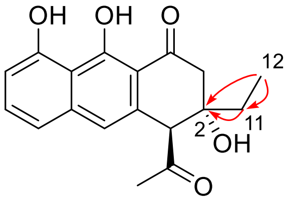

3

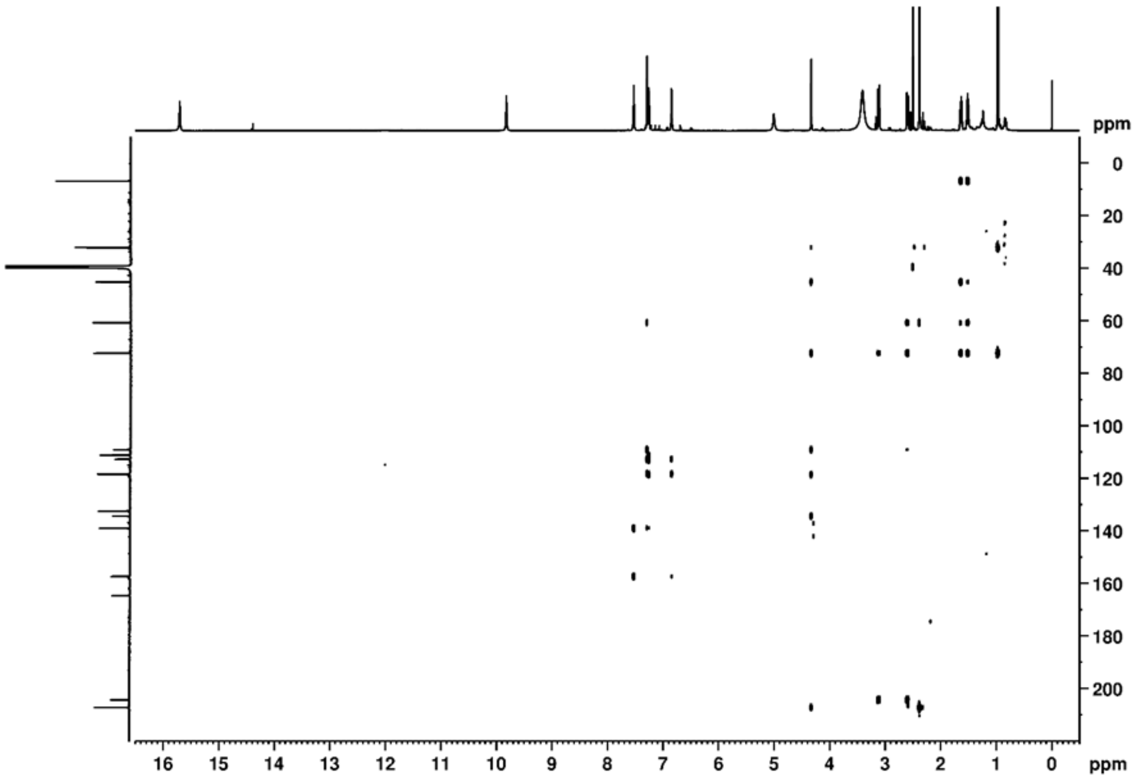

Figure S4. The spectroscopic data of NEN G (**3**).

(F) The NOESY spectrum of **3** (in DMSO-d_6)

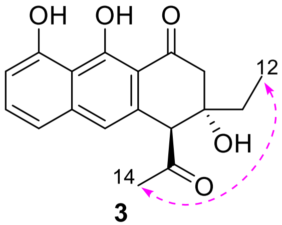

3

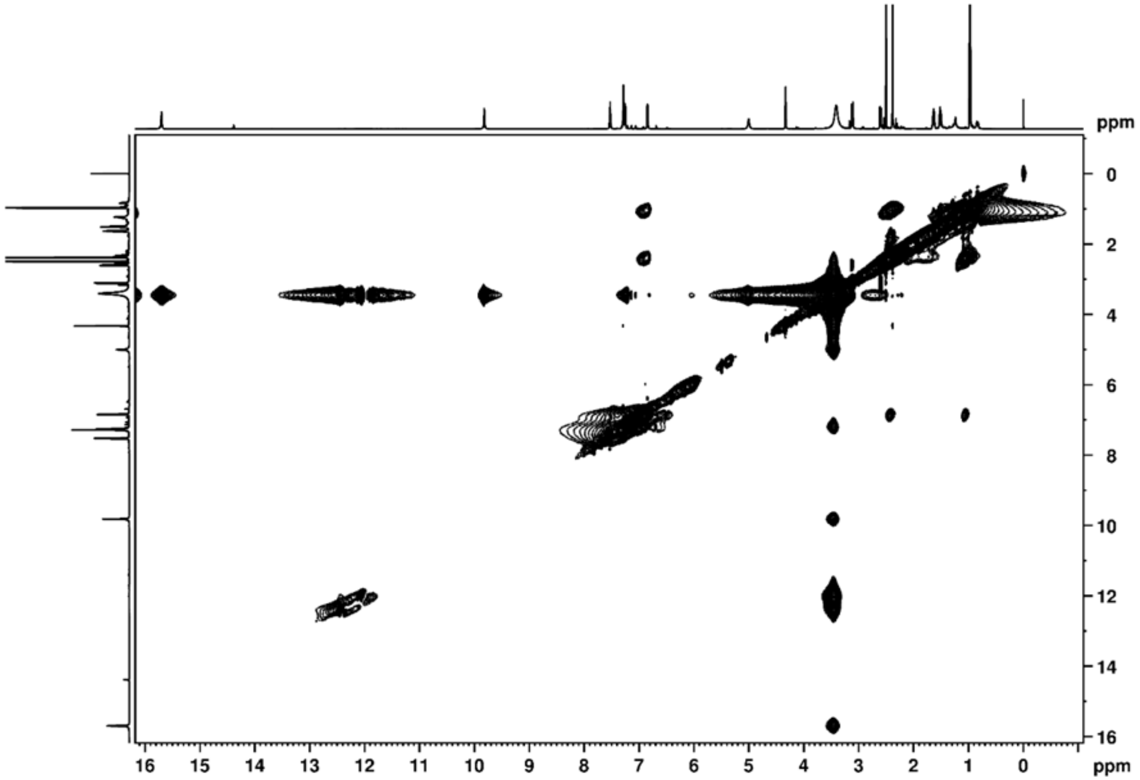

Figure S4. The spectroscopic data of NEN G (**3**).

(G) The ^{1}H-NMR spectrum of **3** (700 MHz for ^{1}H NMR in CD_3OD)

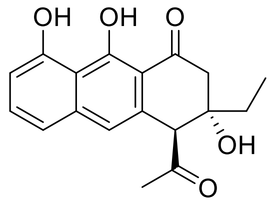

3

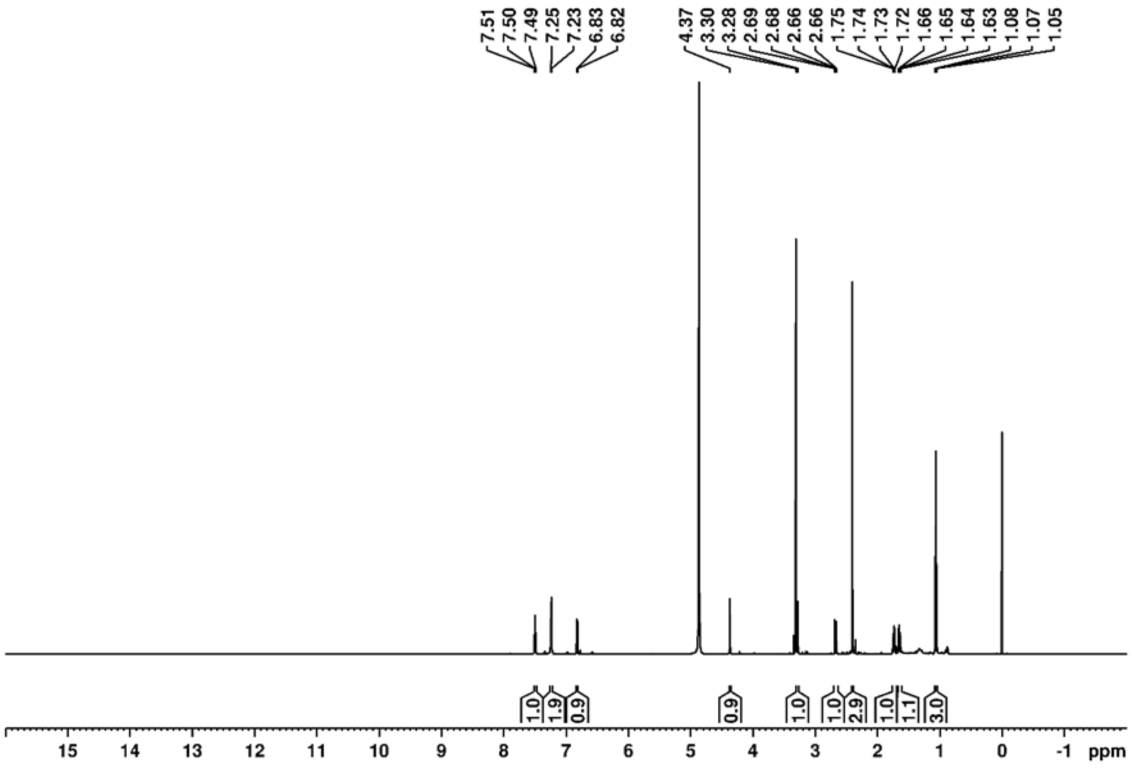

Figure S4. The spectroscopic data of NEN G (**3**).

(H) The ^{13}C-NMR spectrum of NEN G (3) (700 MHz for ^{13}C NMR in CD_3OD)

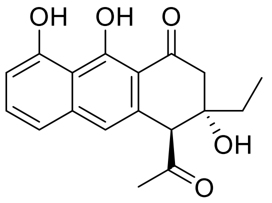

3

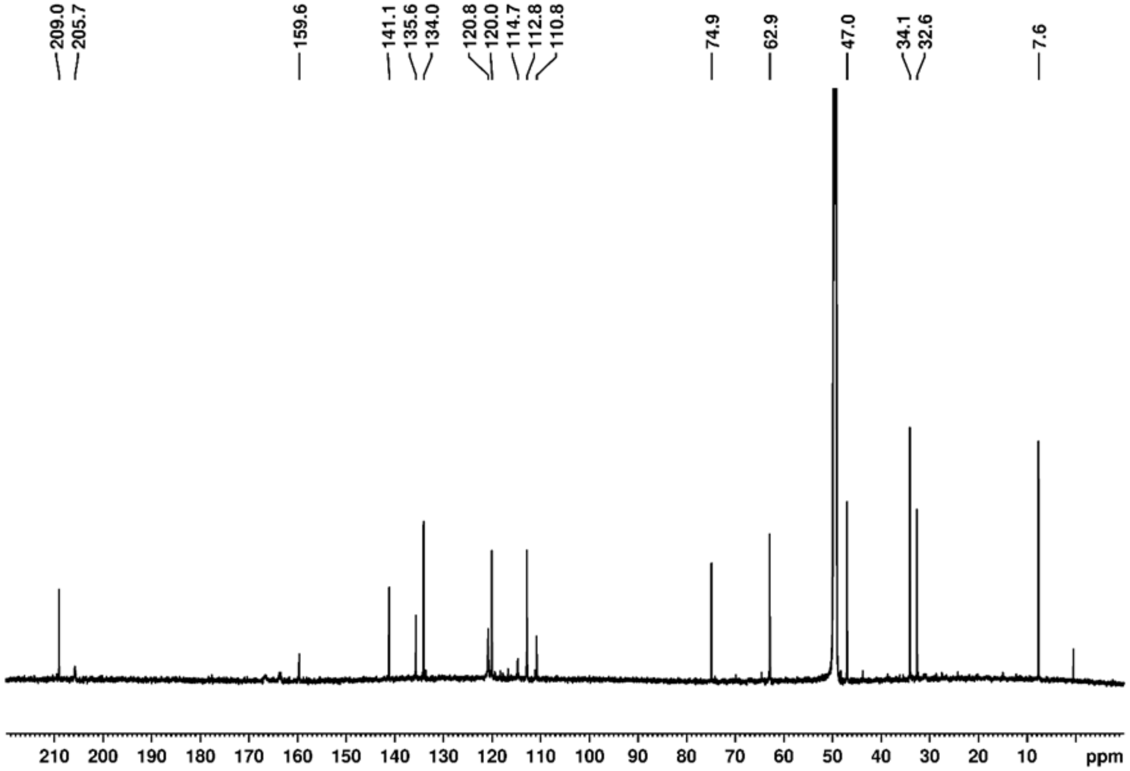

Figure S4. The spectroscopic data of NEN G (**3**).

(I) The HSQC spectrum of **3** (in CD_3OD)

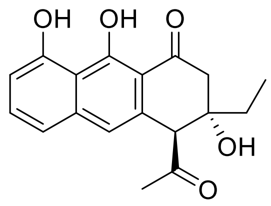

3

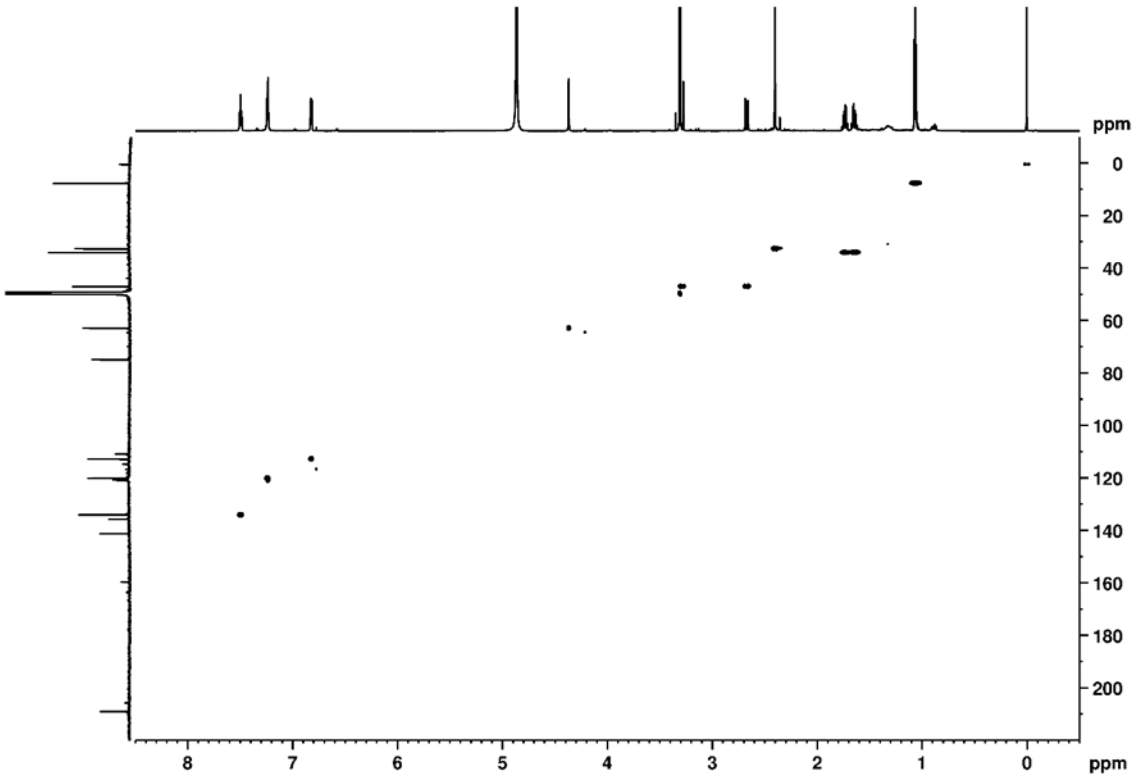

Figure S5. The spectroscopic data of NEN G (**3**).

(J) The ^{1}H-^{1}H COSY spectrum of **3** (in CD_3OD)

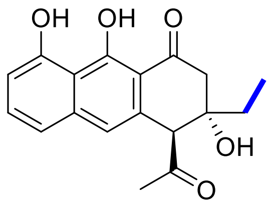

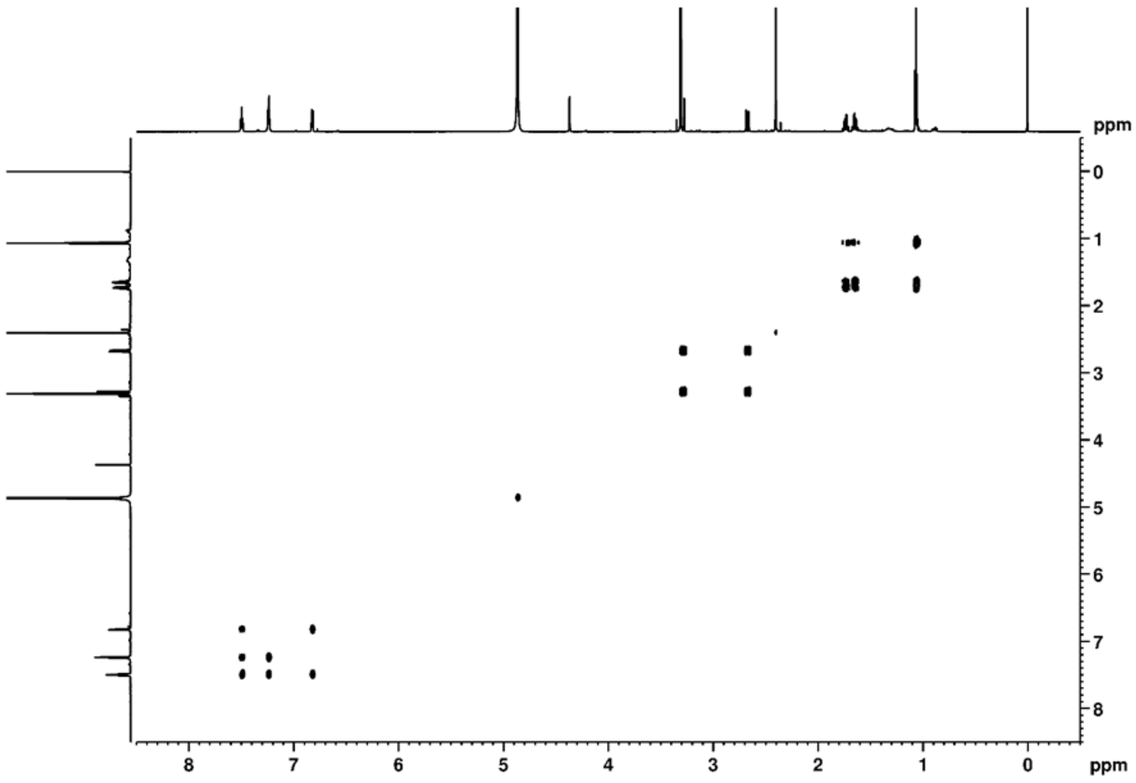

Figure S4. The spectroscopic data of NEN G (**3**).

(K) The HMBC spectrum of **3** (in CD_3OD)

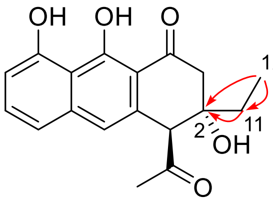

3

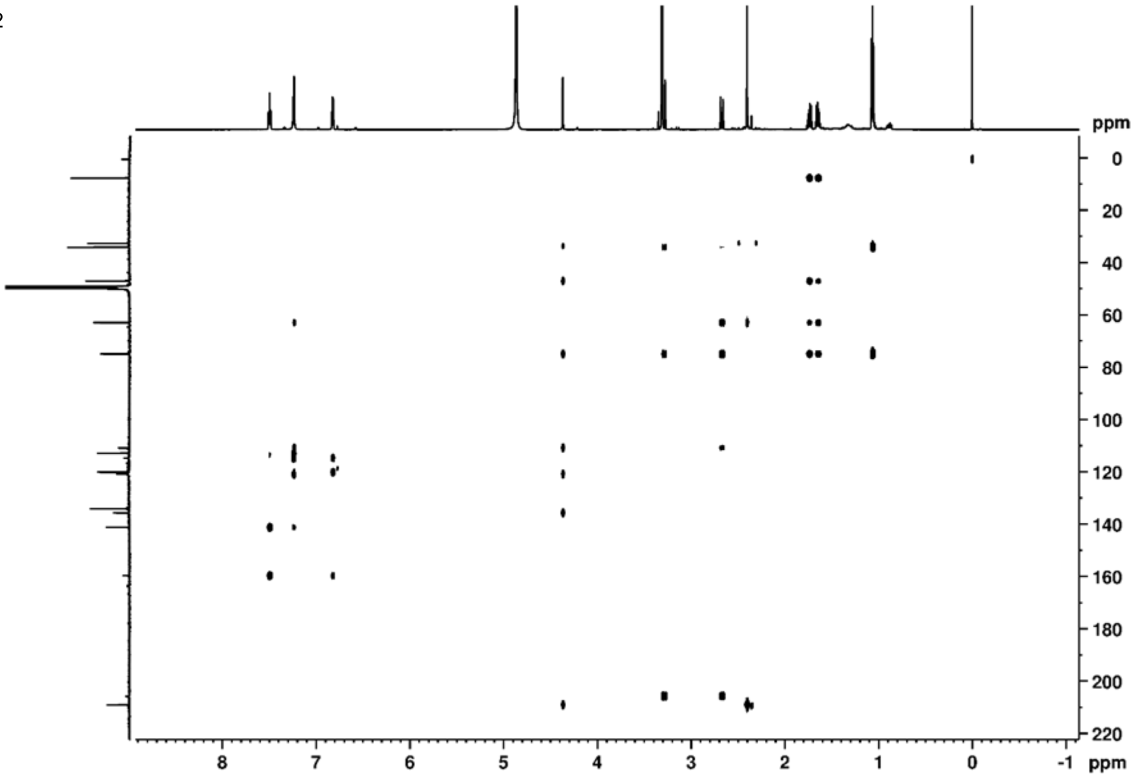

Figure S4. The spectroscopic data of NEN G (**3**).

(L) The UV spectrum of **3**

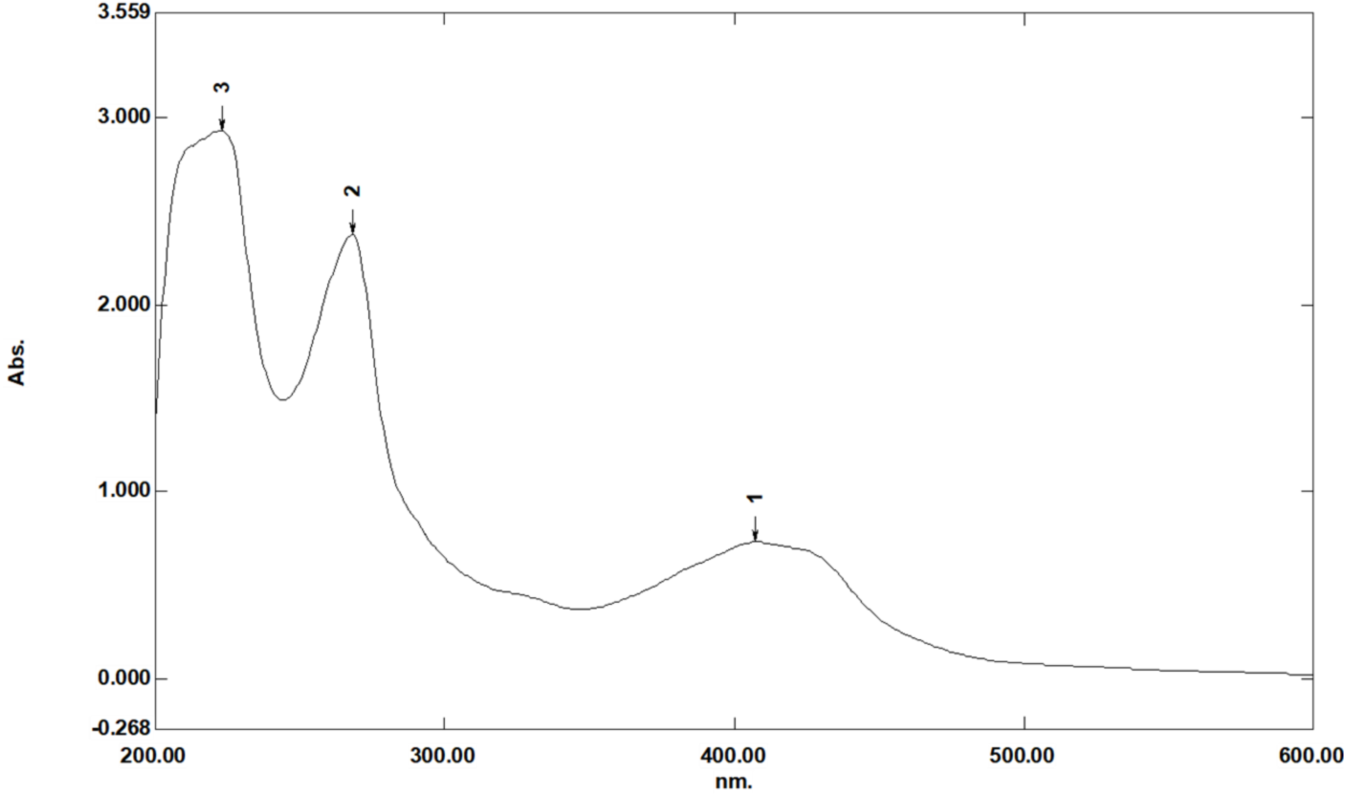

Figure S4. The spectroscopic data of NEN G (**3**).

(M) The IR spectrum of **3**

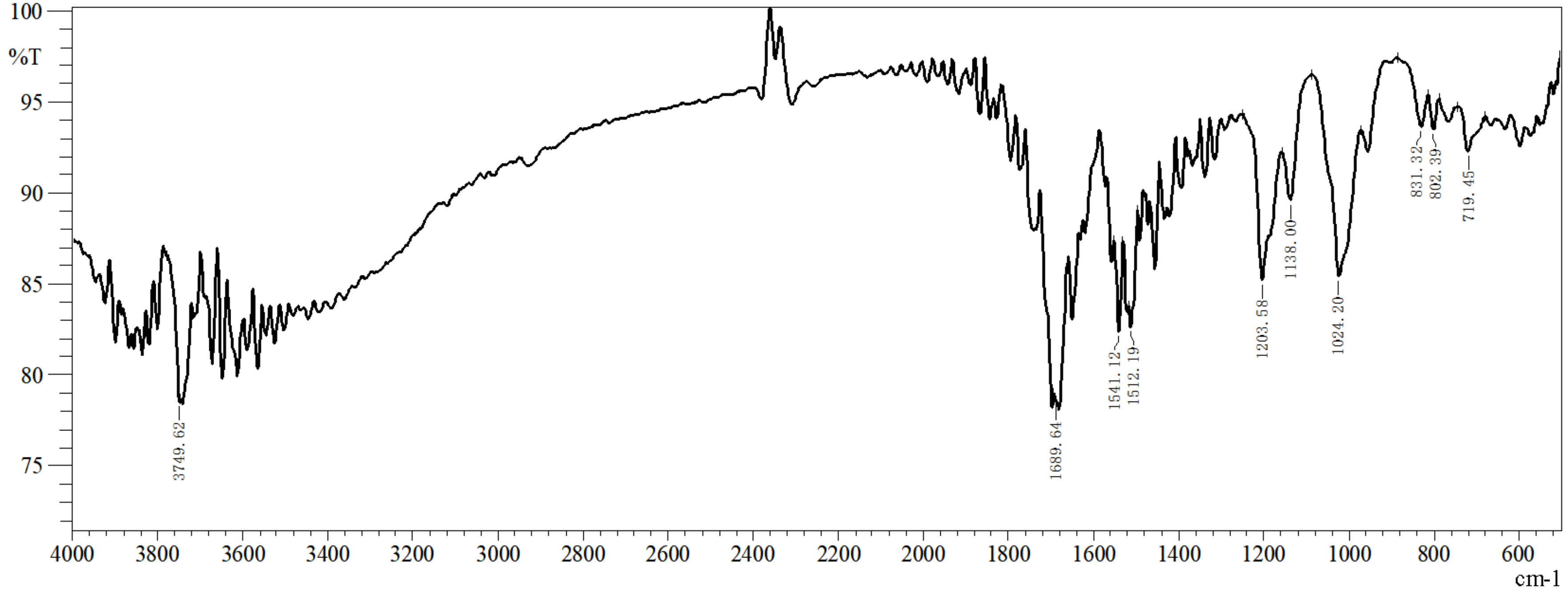

Figure S5. The optimized conformers above 1% population of **3a**.

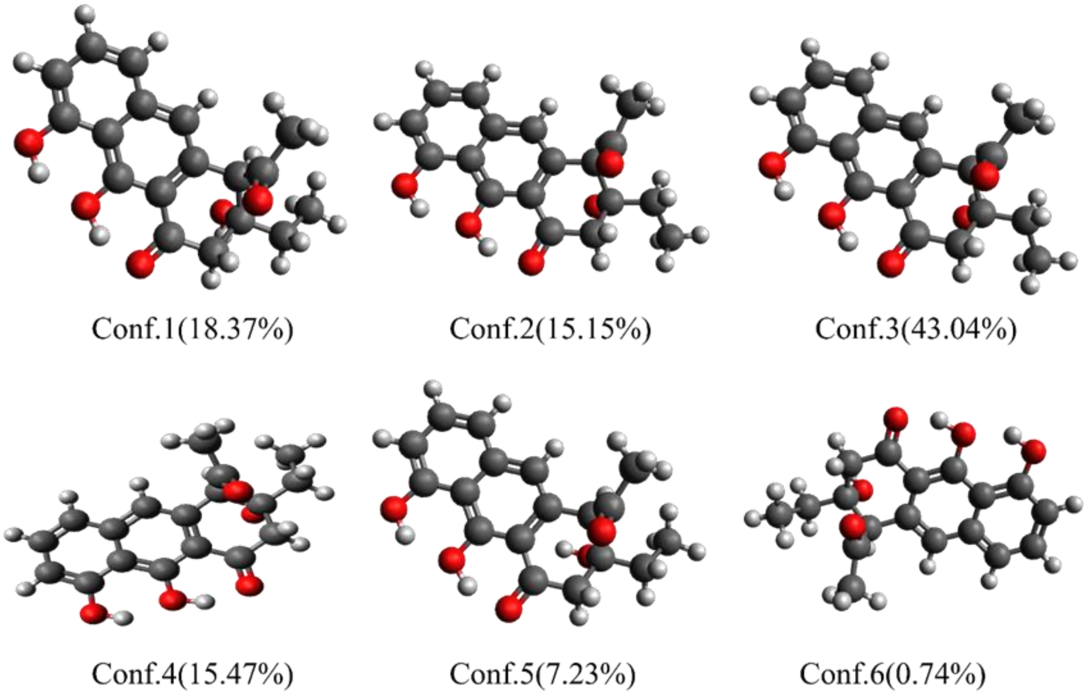

Figure S6. The spectroscopic data of NEN H (**4**).

(A) The HRESIMS spectrum of **4**

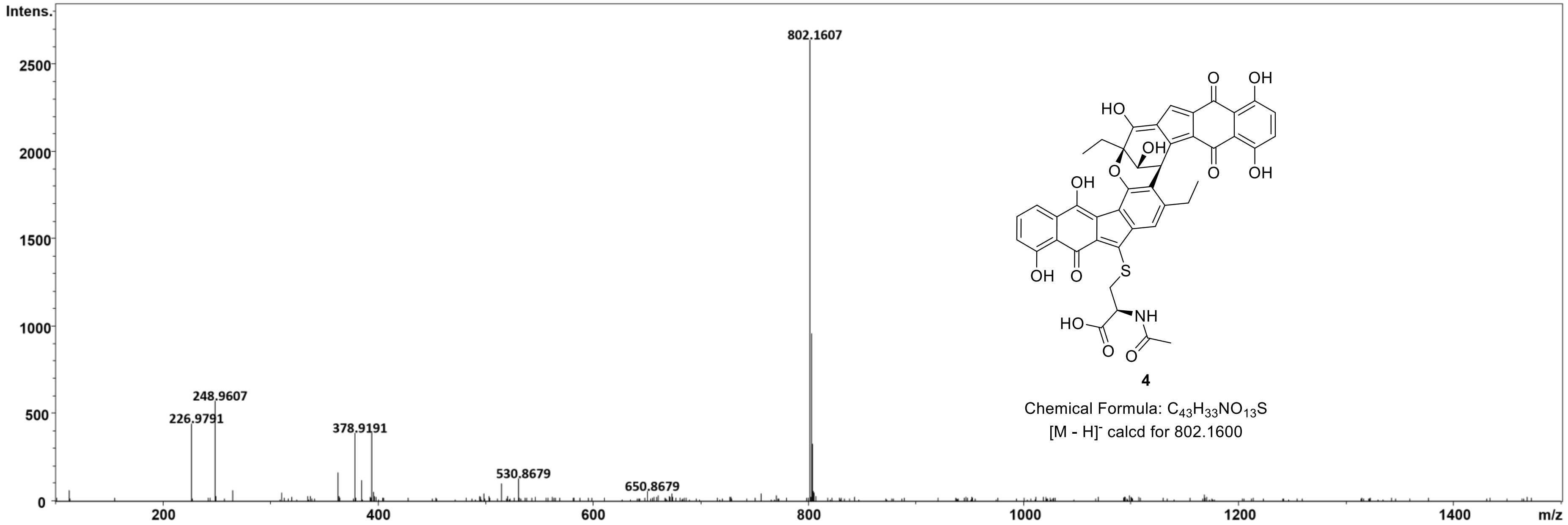

Figure S6. The spectroscopic data of NEN H (**4**).

(B) The ^{1}H-NMR spectrum of **4** (700 MHz for ^{1}H NMR in DMSO-d_6)

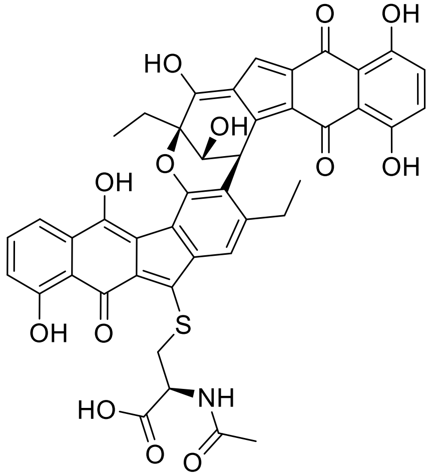

4

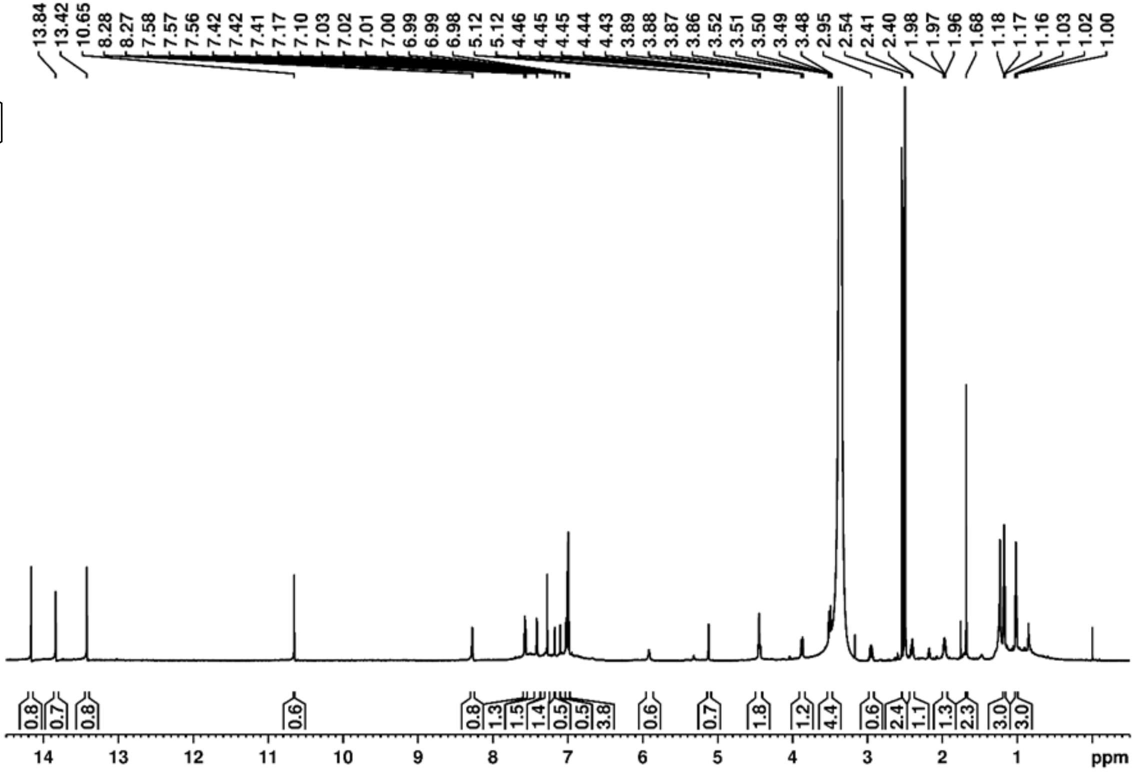

Figure S6. The spectroscopic data of NEN H (**4**).

(C) The ^{13}C-NMR spectrum of **4** (175 MHz for ^{13}C NMR in DMSO-d_6)

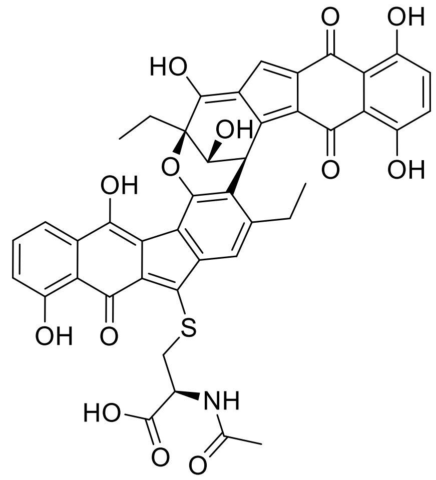

4

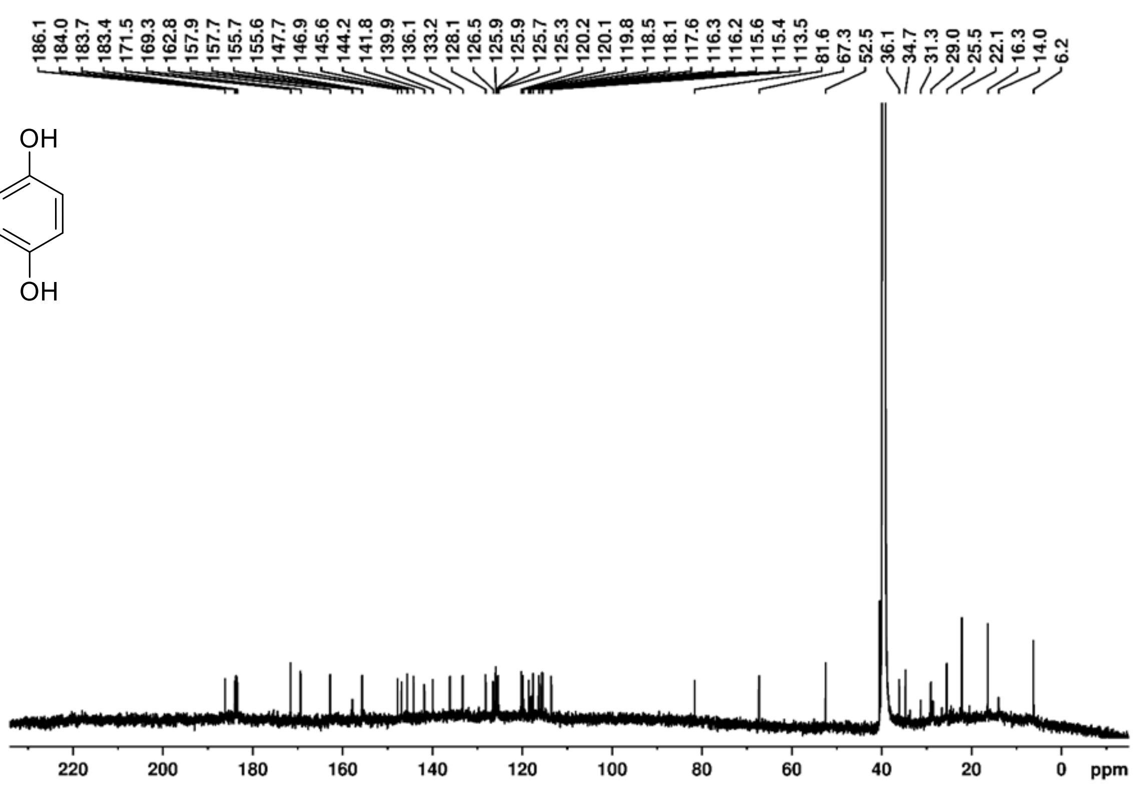

Figure S6. The spectroscopic data of NEN H (**4**).

(D) The DEPT135 spectrum of **4** (175 MHz for ^{13}C NMR in DMSO-d_6)

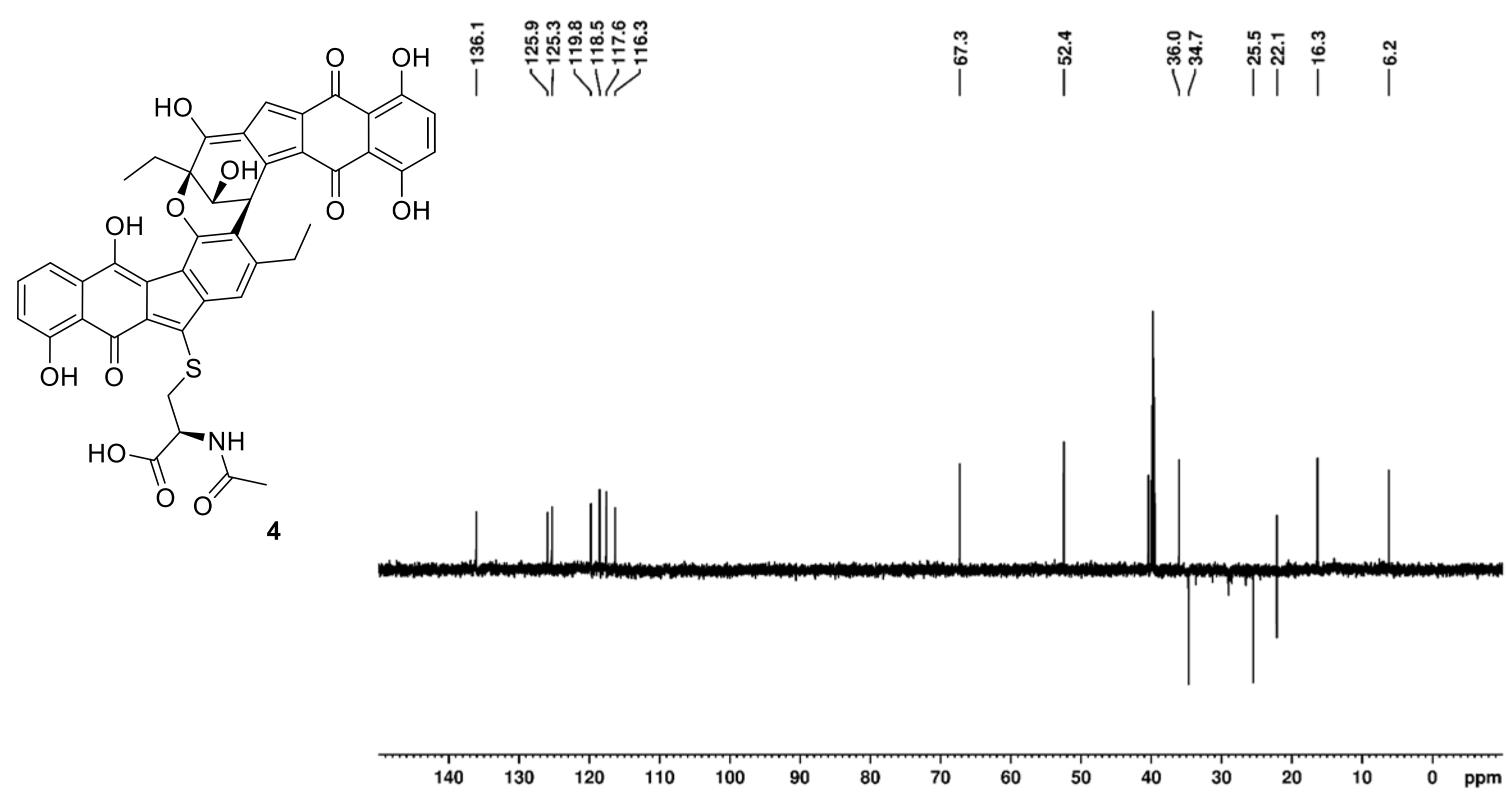

Figure S6. The spectroscopic data of NEN H (**4**).

(E) The ^{1}H-^{1}H COSY spectrum of **4**

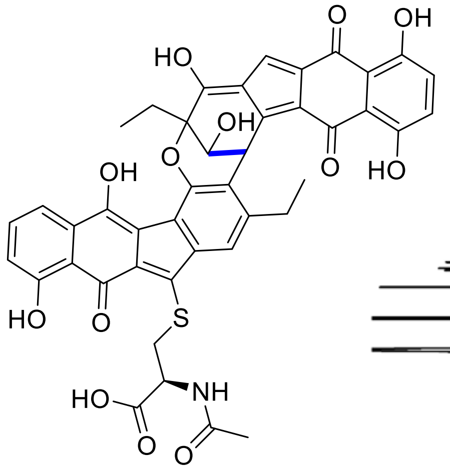

4

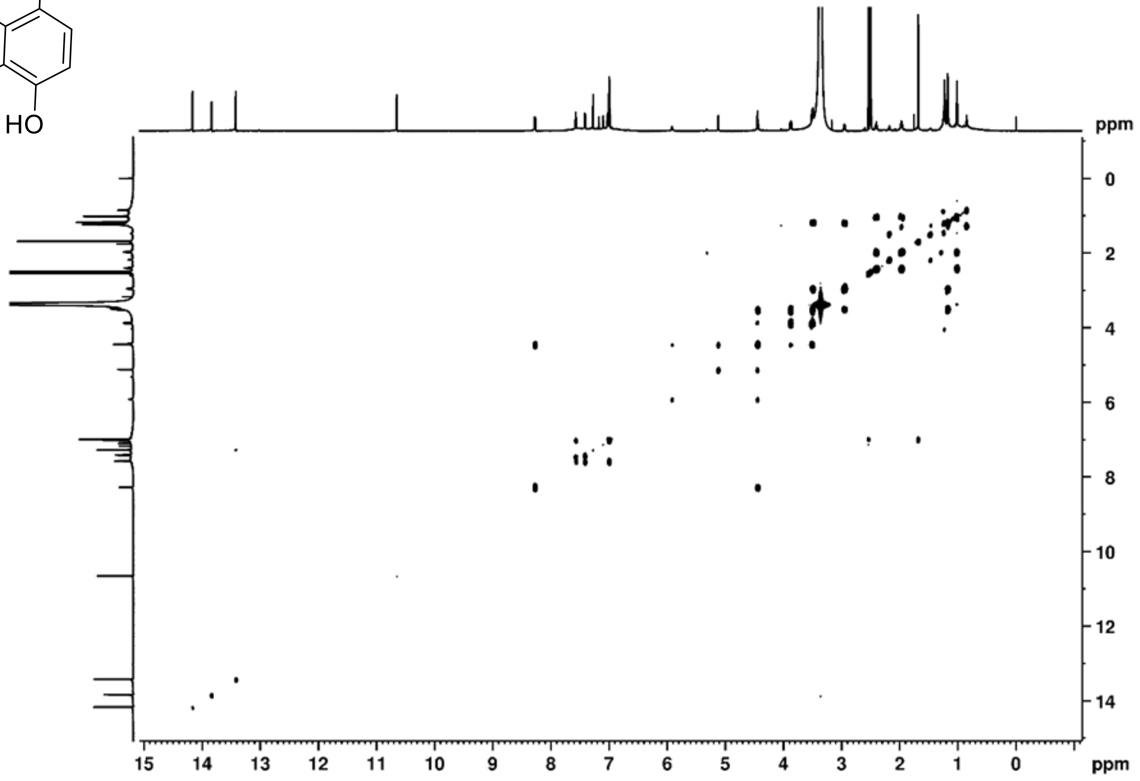

Figure S6. The spectroscopic data of NEN H (**4**).

(F) The HSQC spectrum of **4**

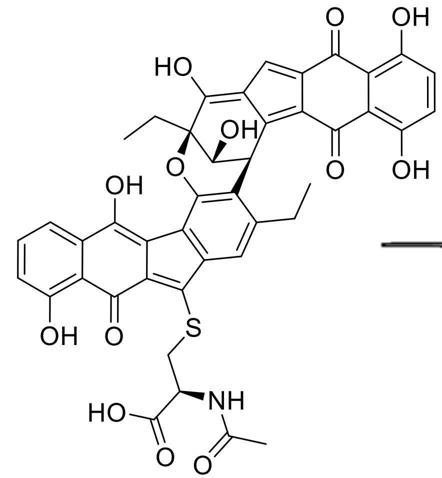

4

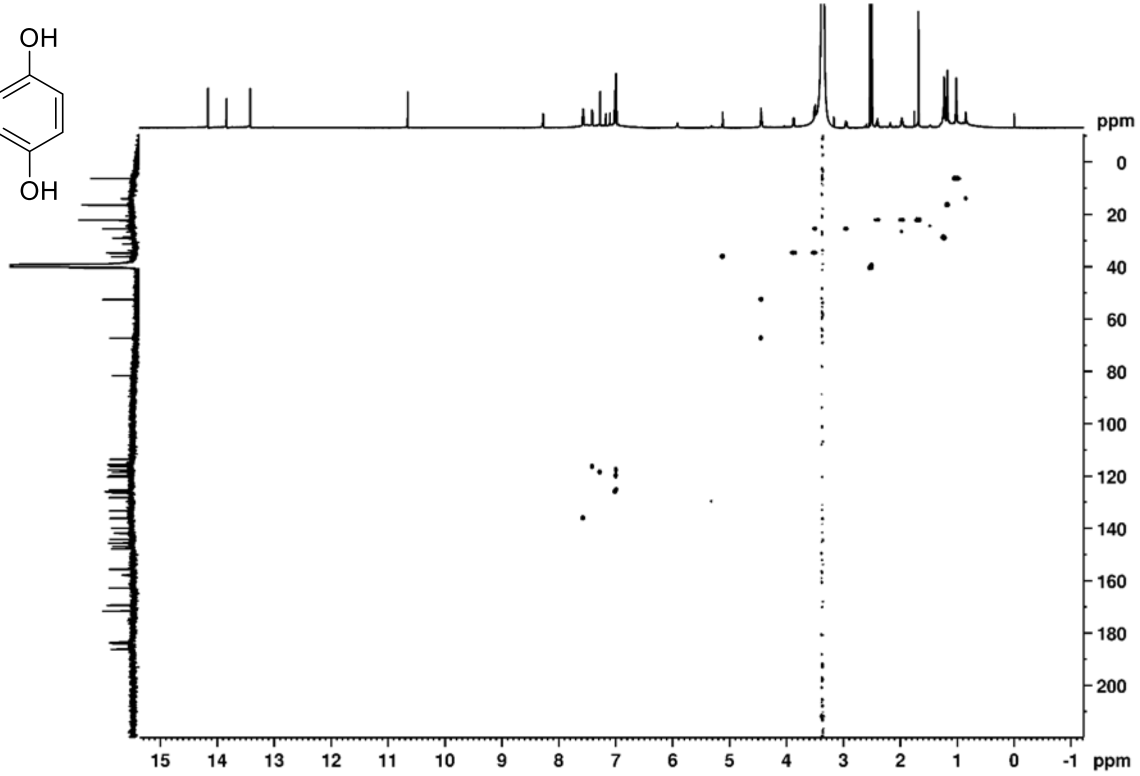

Figure S6. The spectroscopic data of NEN H (**4**).

(G) The HMBC spectrum of **4**

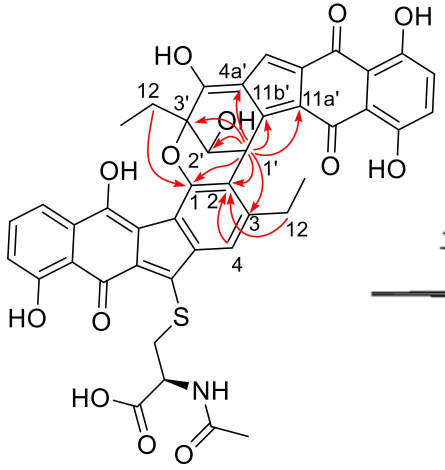

4

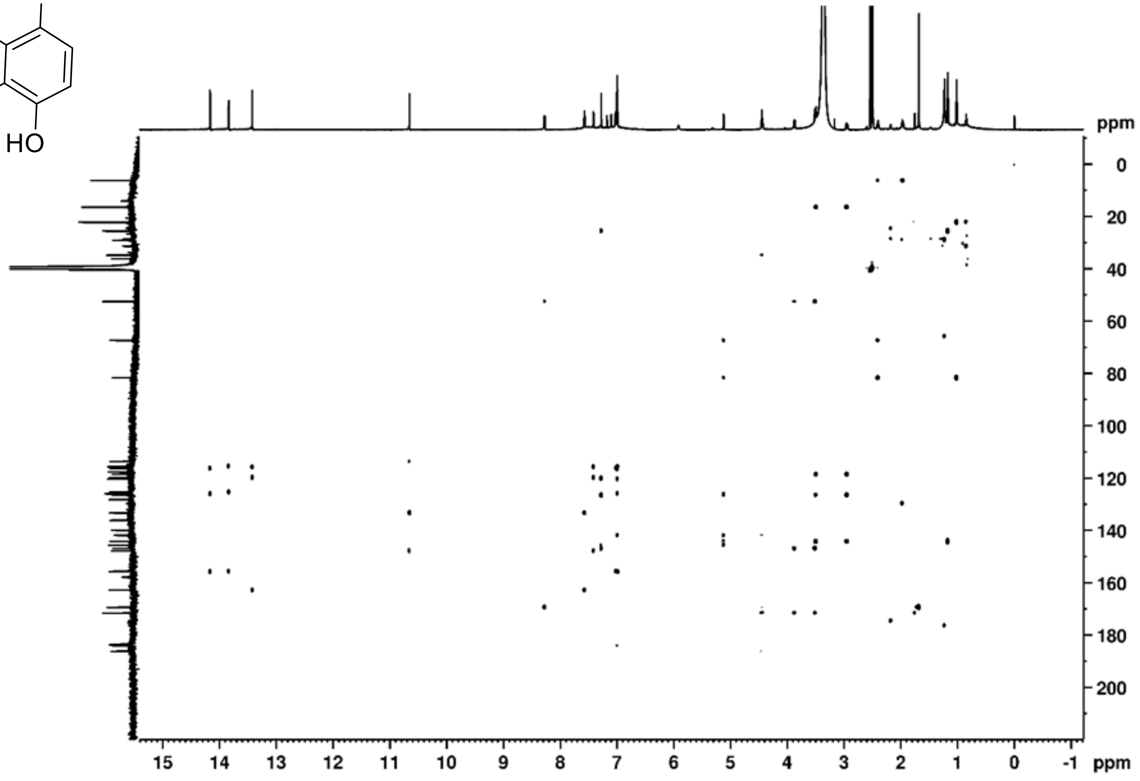

Figure S6. The spectroscopic data of NEN H (**4**).

(H) The NOESY spectrum of **4**

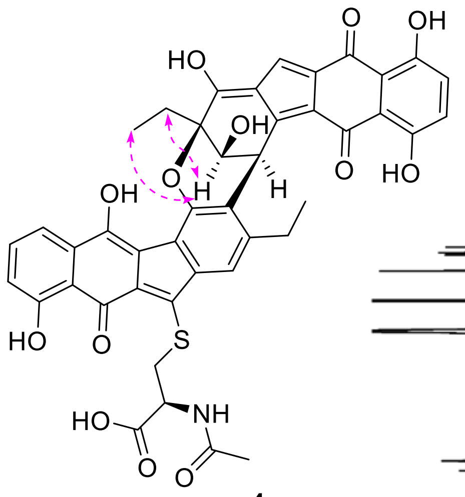

4

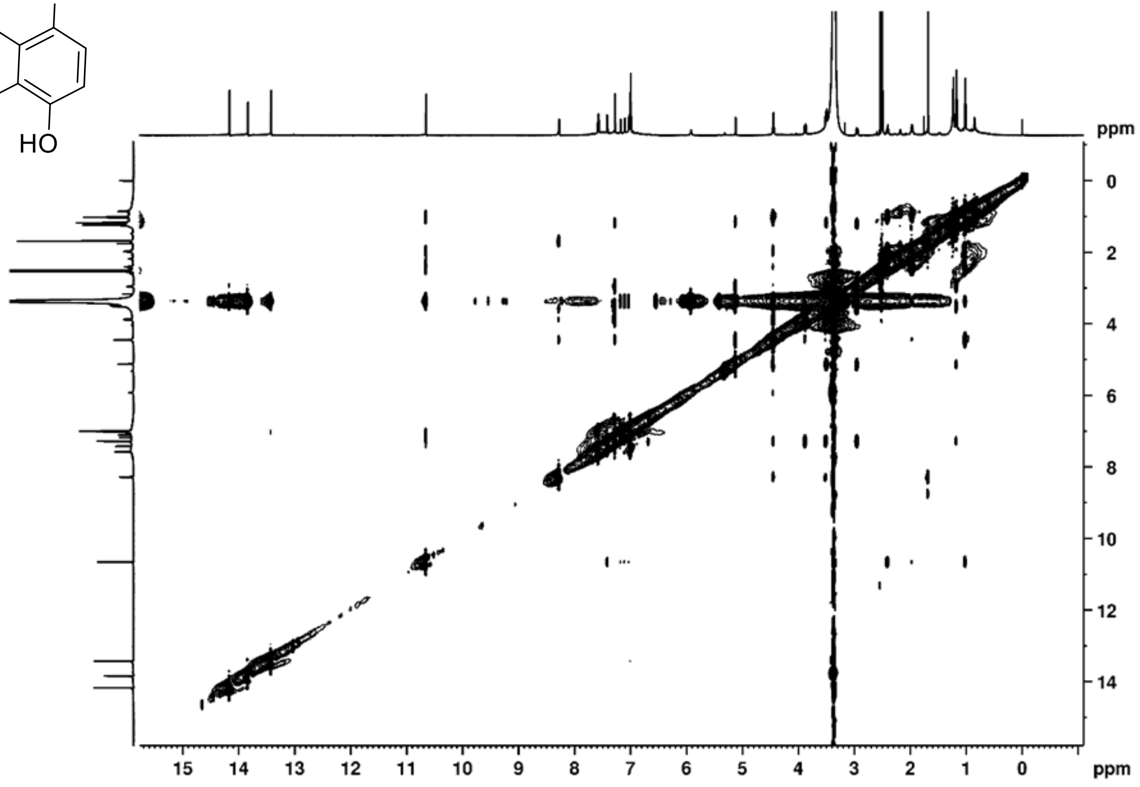

Figure S6. The spectroscopic data of NEN H (**4**).

(I) The UV spectrum of **4**

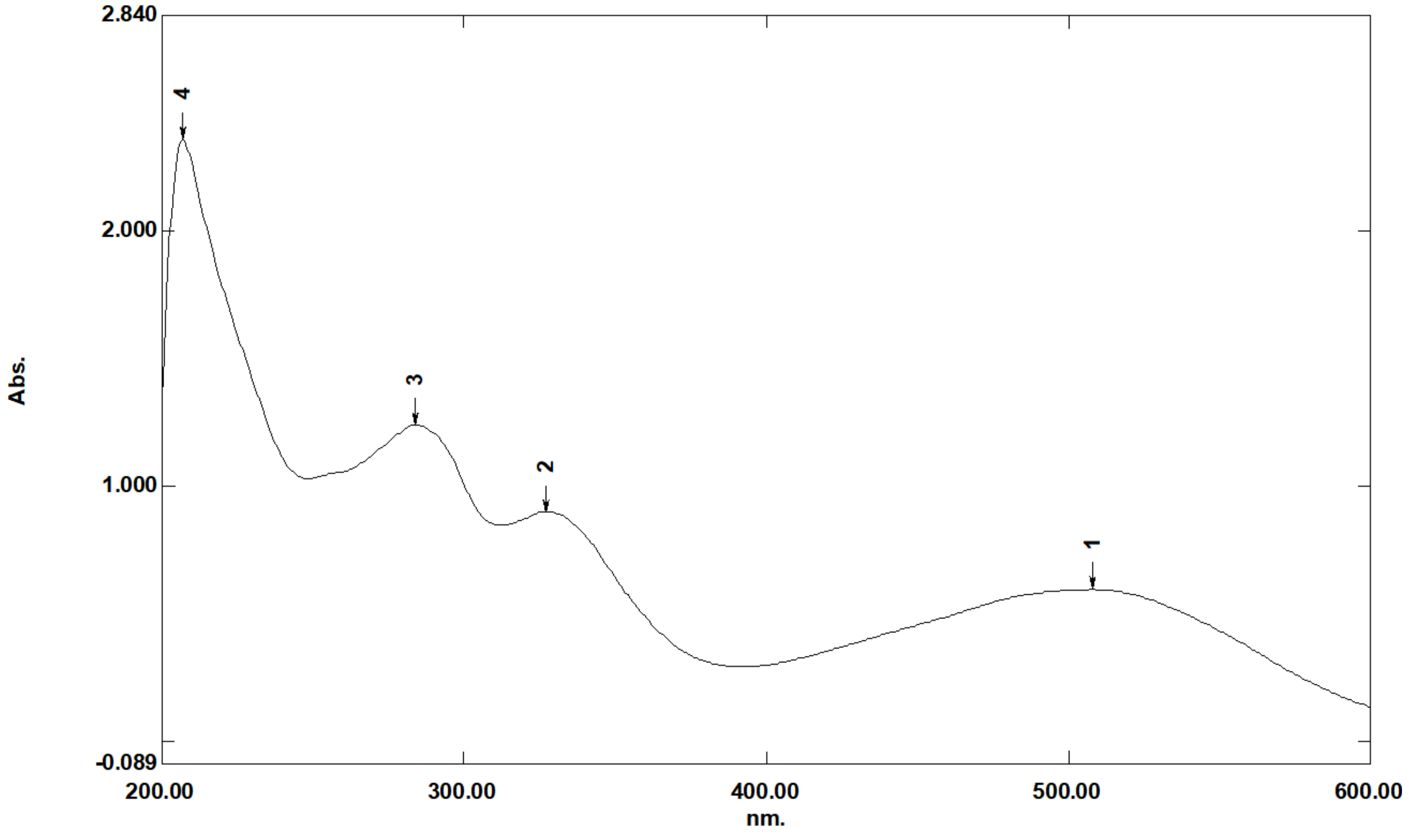

Figure S6. The spectroscopic data of NEN H (**4**).

(J) The IR spectrum of **4**

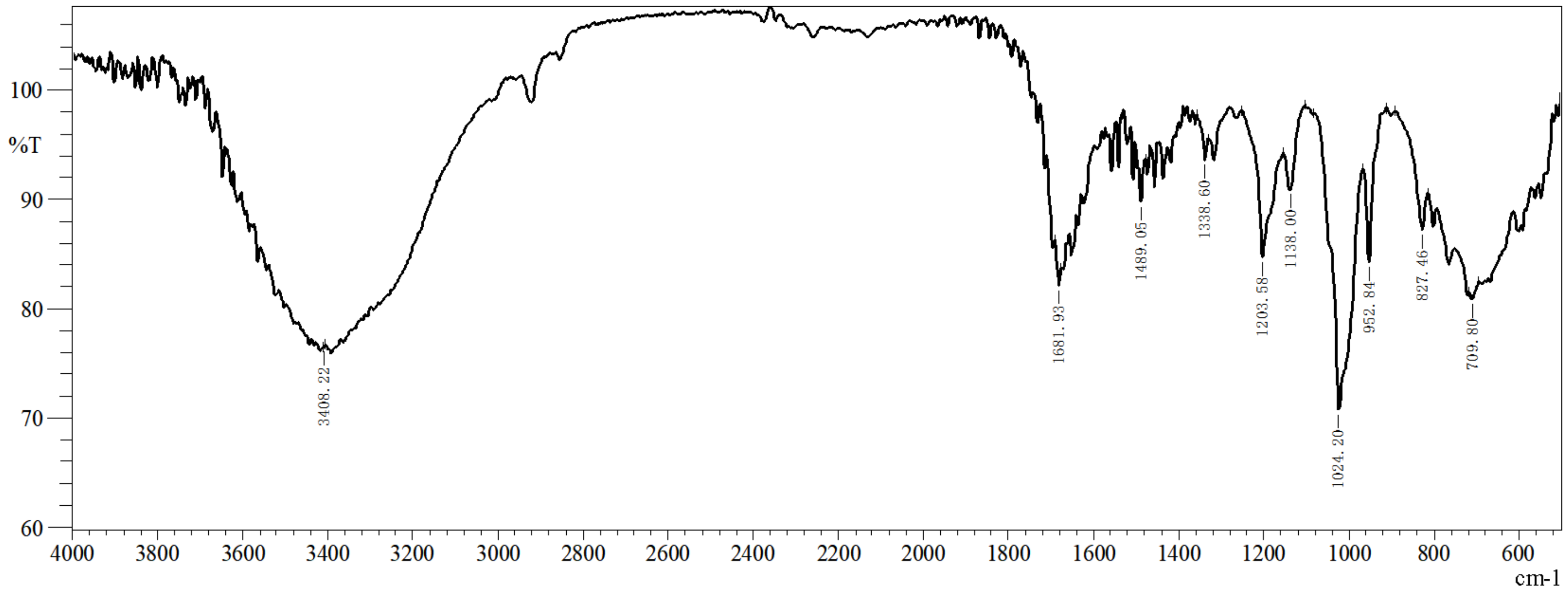

Figure S7. Comparison of the Exp ECD of **4** and **5** with those Calcd for **4c** and **4d**.

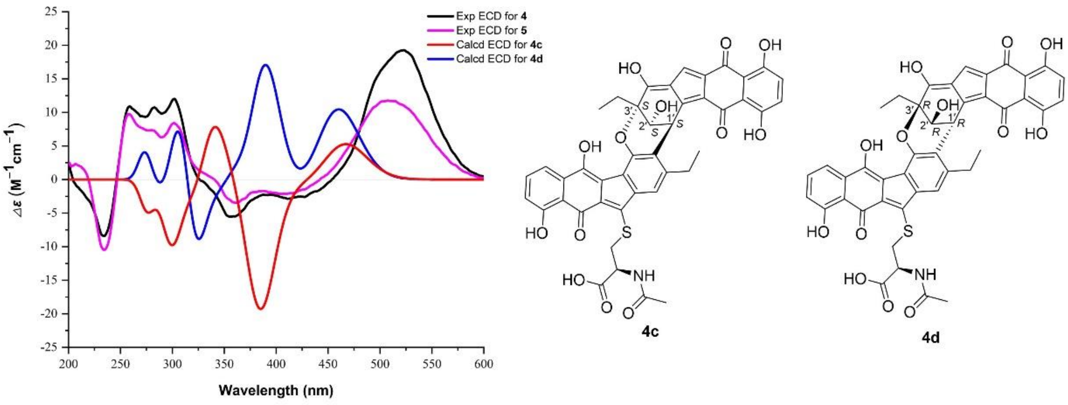

Figure S8. The optimized conformers above 1% population of **4a**.

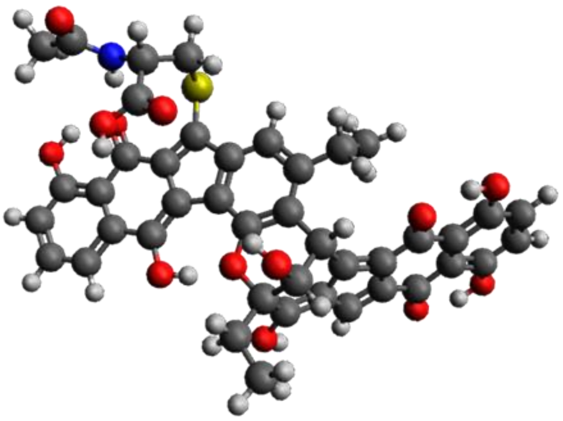

Conf.1(100%)

Figure S9. The spectroscopic data of NEN I (**5**).

(A) The HRESIMS spectrum of **5**

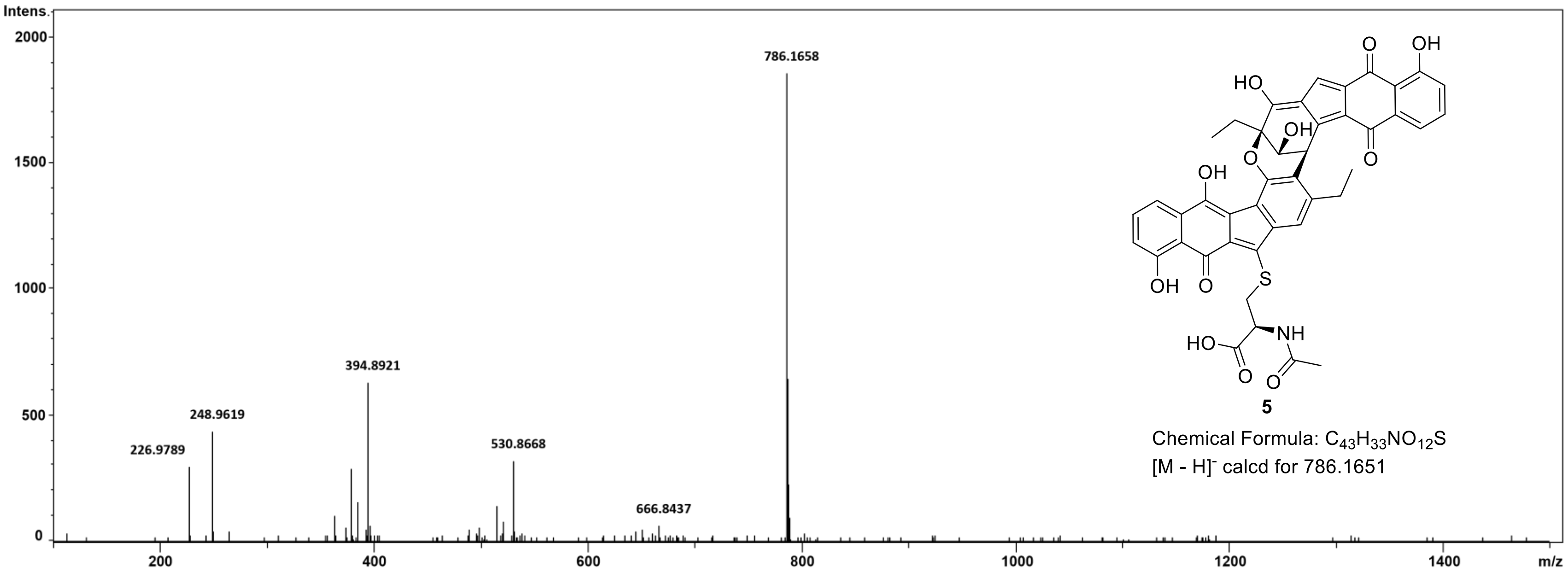

Figure S9. The spectroscopic data of NEN I (**5**).

(B) The ^{1}H-NMR spectrum of **5** (700 MHz for ^{1}H NMR in DMSO-d_6)

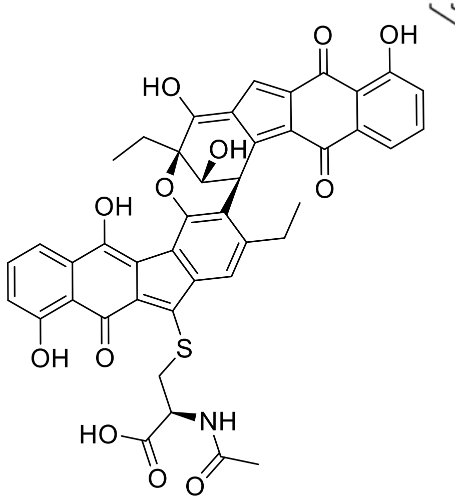

5

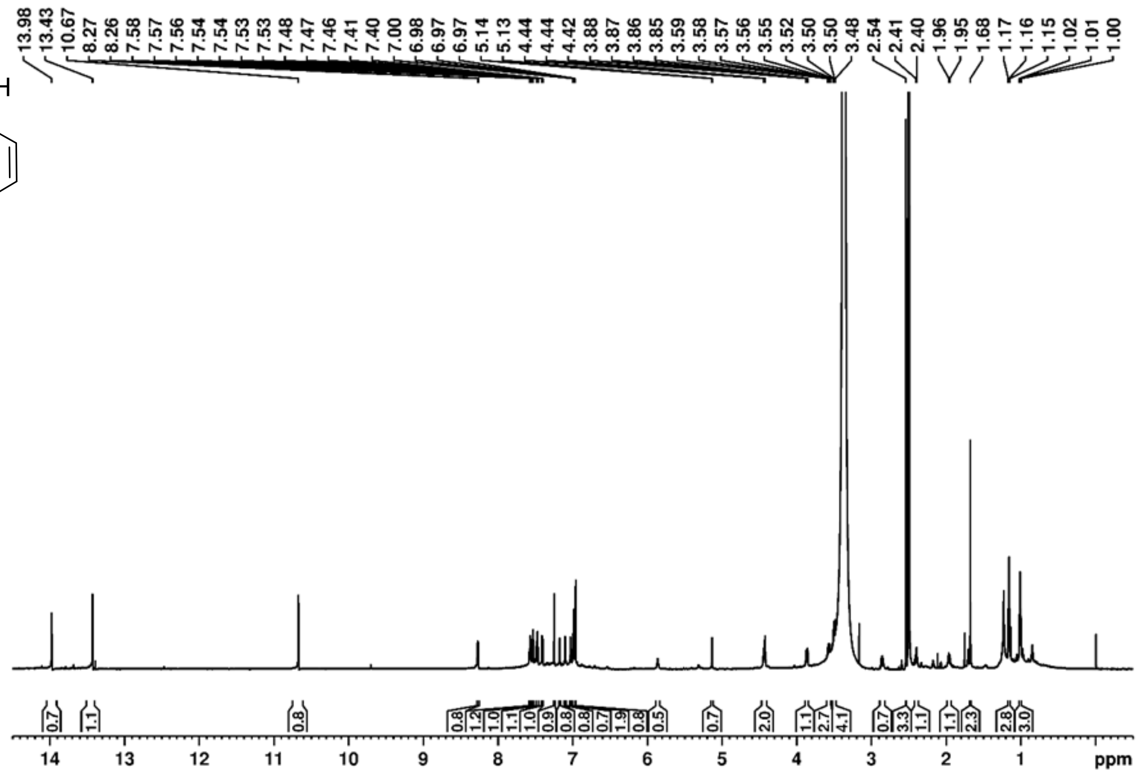

Figure S9. The spectroscopic data of NEN I (**5**).

(C) The ^{13}C-NMR spectrum of NEN I (**5**) (175 MHz for ^{13}C NMR in DMSO-d_6)

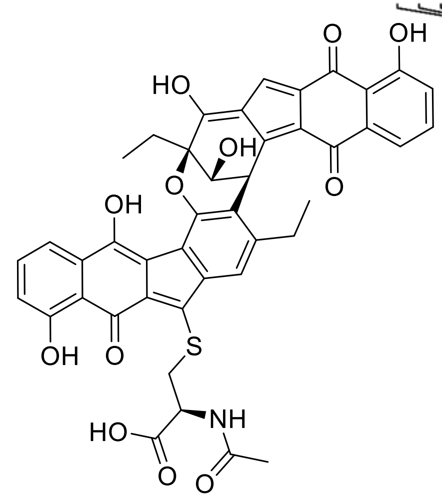

5

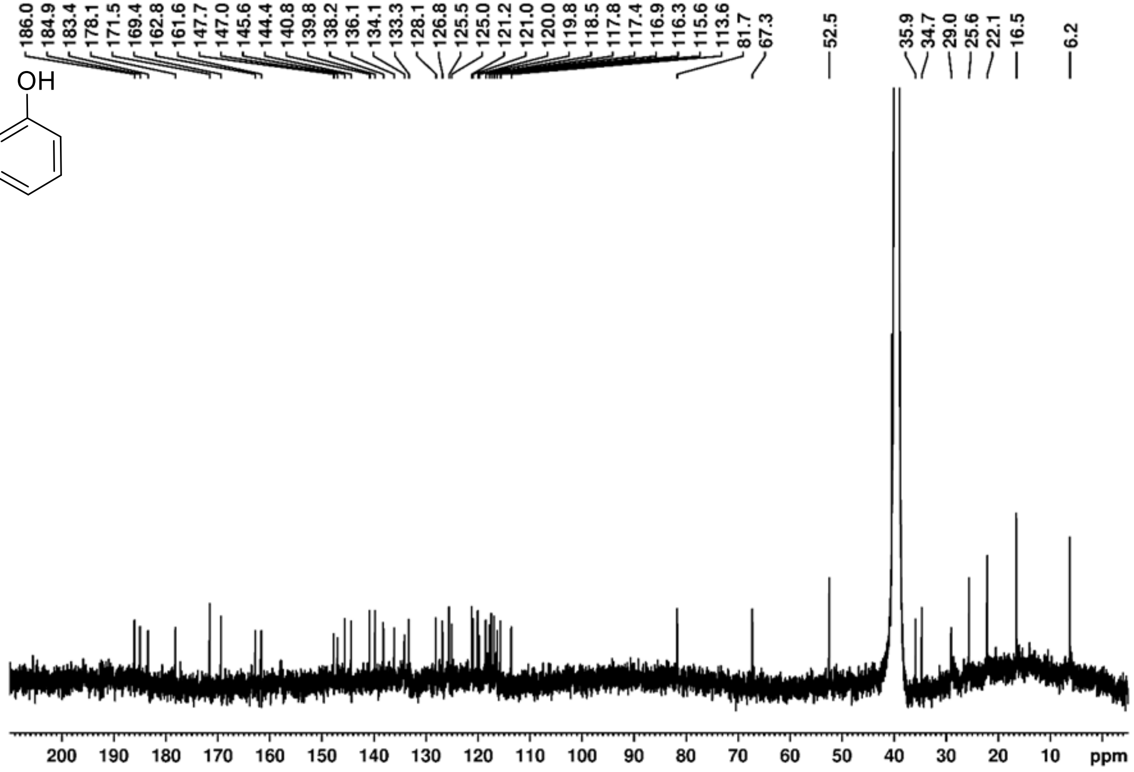

Figure S9. The spectroscopic data of NEN I (**5**).

(D) The DEPT135 spectrum of **5** (175 MHz for ^{13}C NMR in DMSO-d_6)

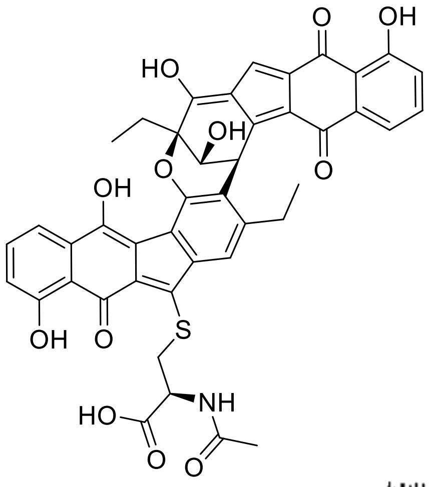

5

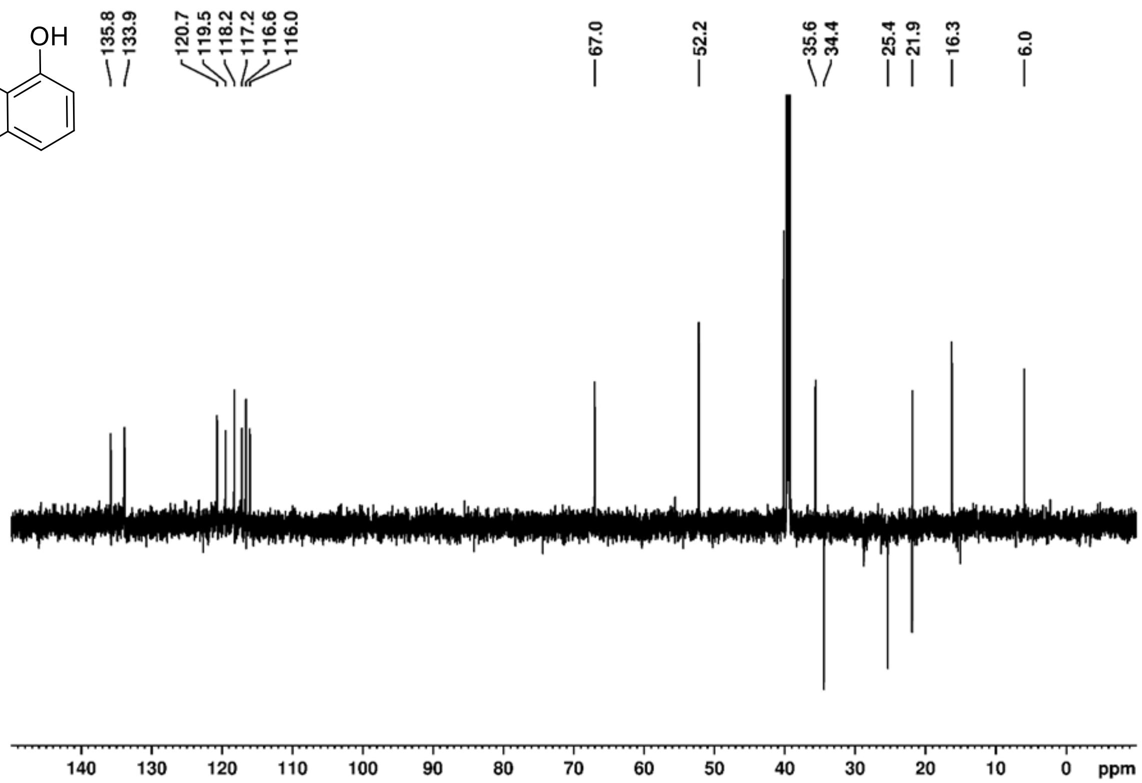

Figure S9. The spectroscopic data of NEN I (**5**).

(E) The ^{1}H-^{1}H COSY spectrum of **5**

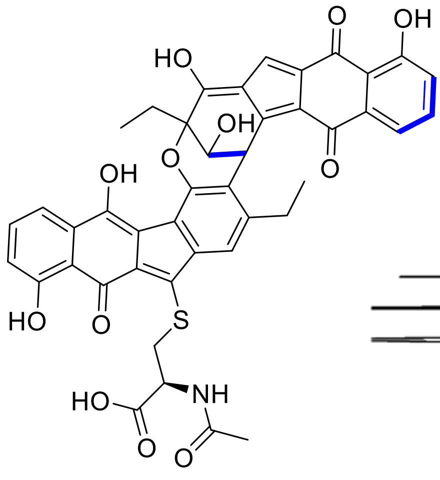

5

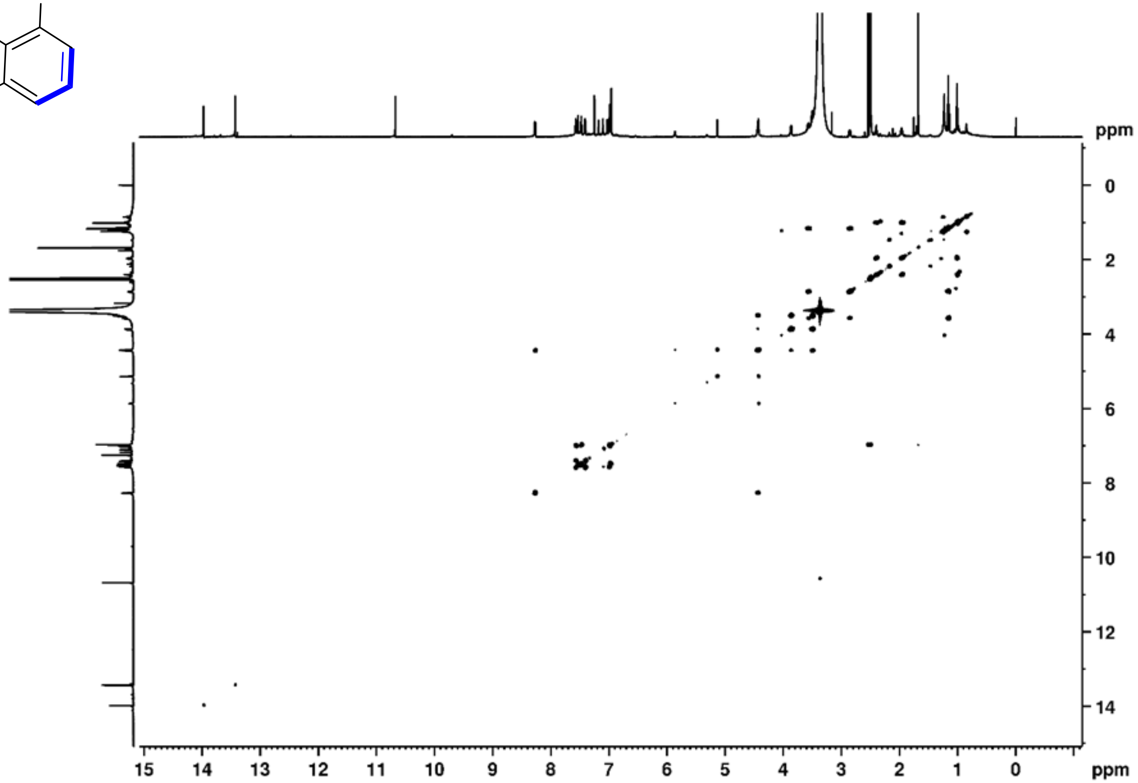

Figure S9. The spectroscopic data of NEN I (**5**).

(F) The HSQC spectrum of **5**

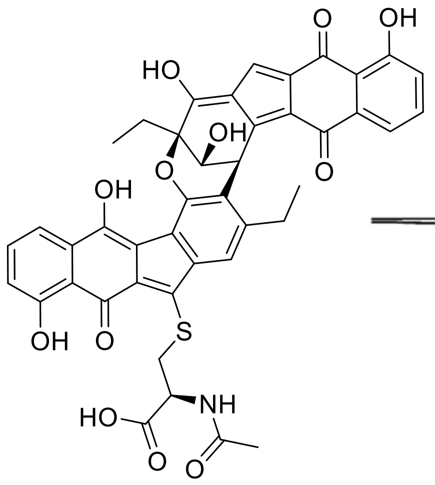

5

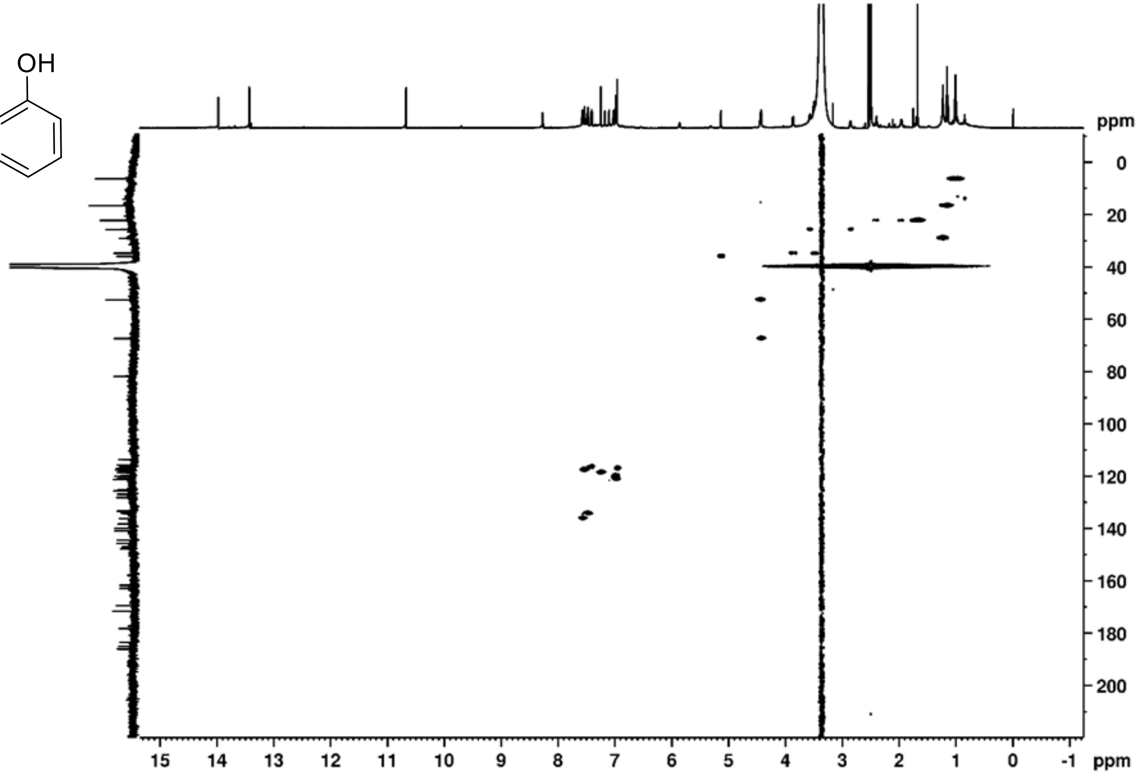

Figure S9. The spectroscopic data of NEN I (**5**).

(G) The HMBC spectrum of **5**

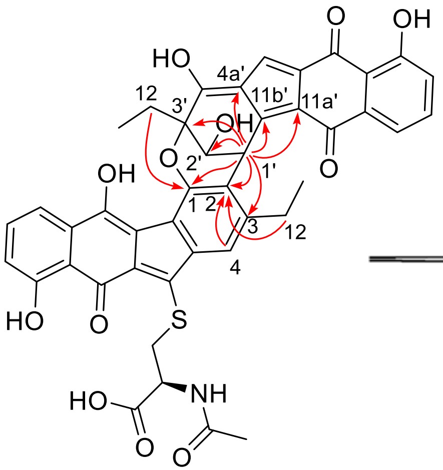

5

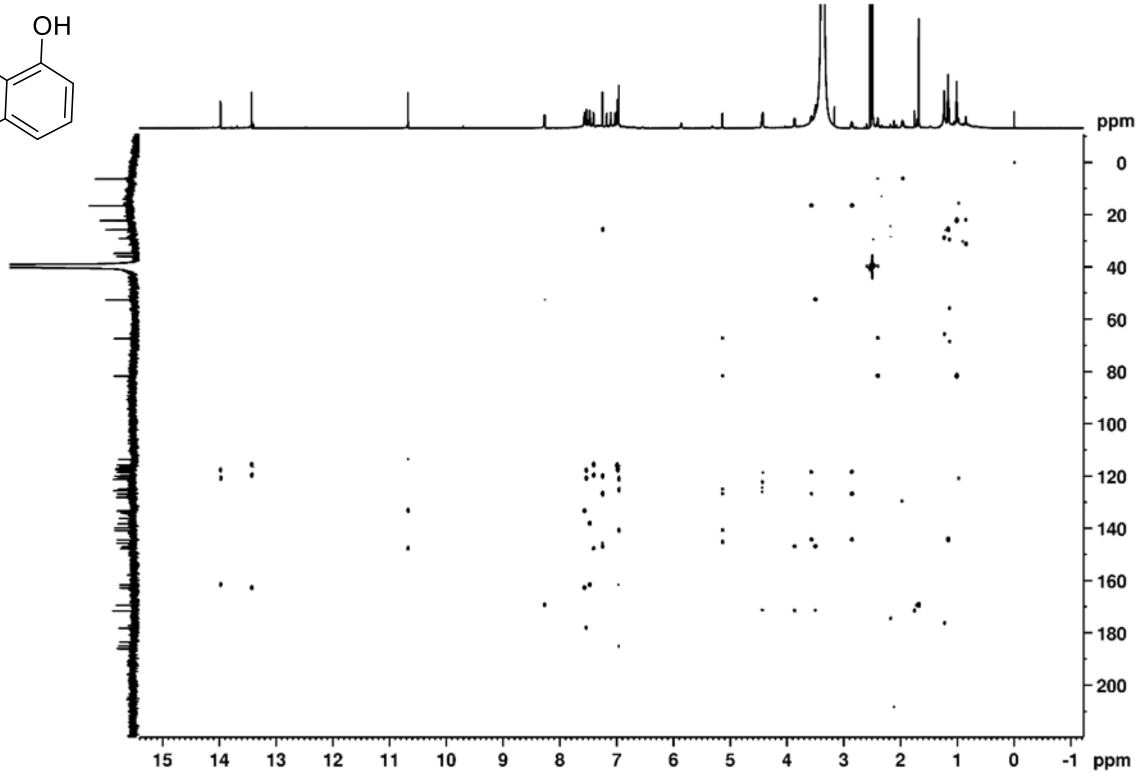

Figure S9. The spectroscopic data of NEN I (**5**).

(H) The NOESY spectrum of **5**

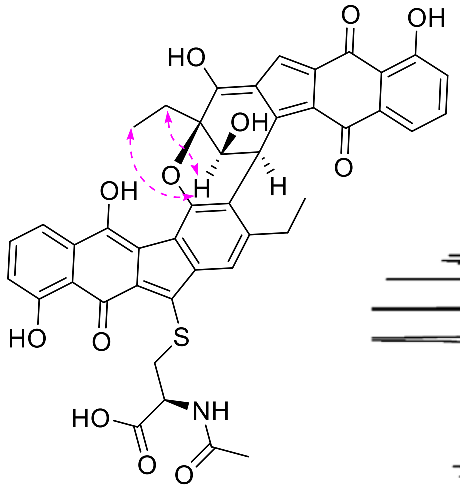

5

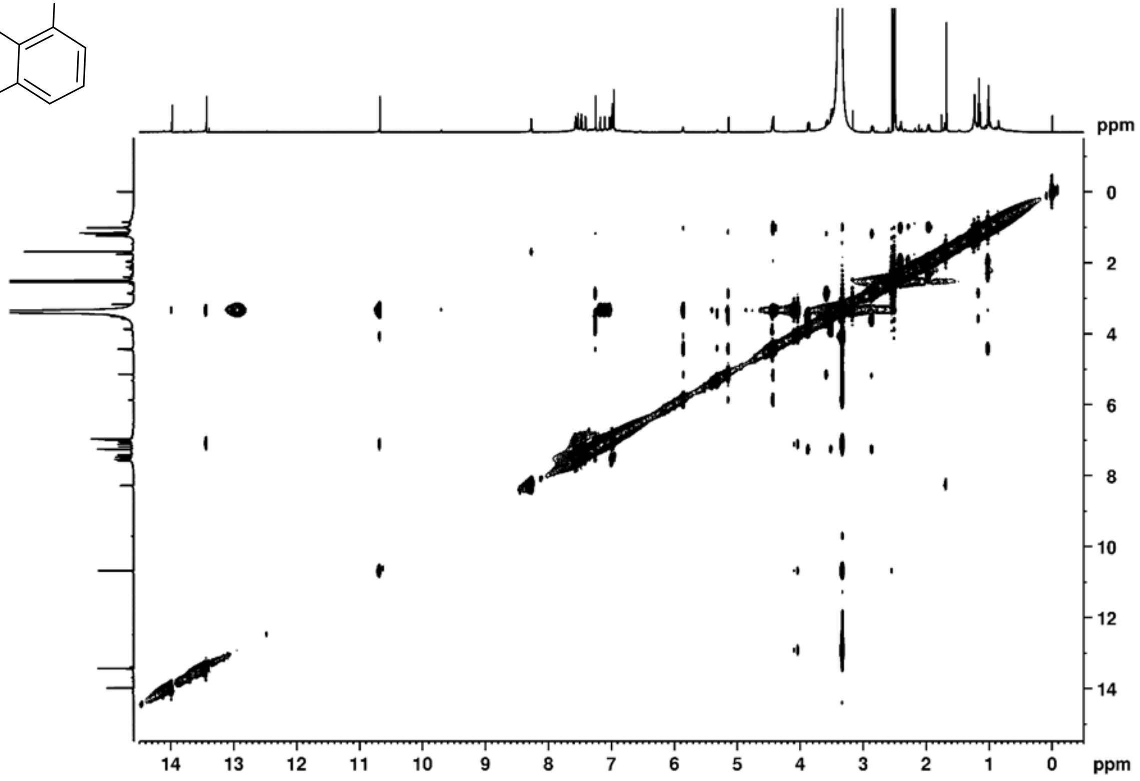

Figure S9. The spectroscopic data of NEN I (**5**).

(I) The UV spectrum of **5**

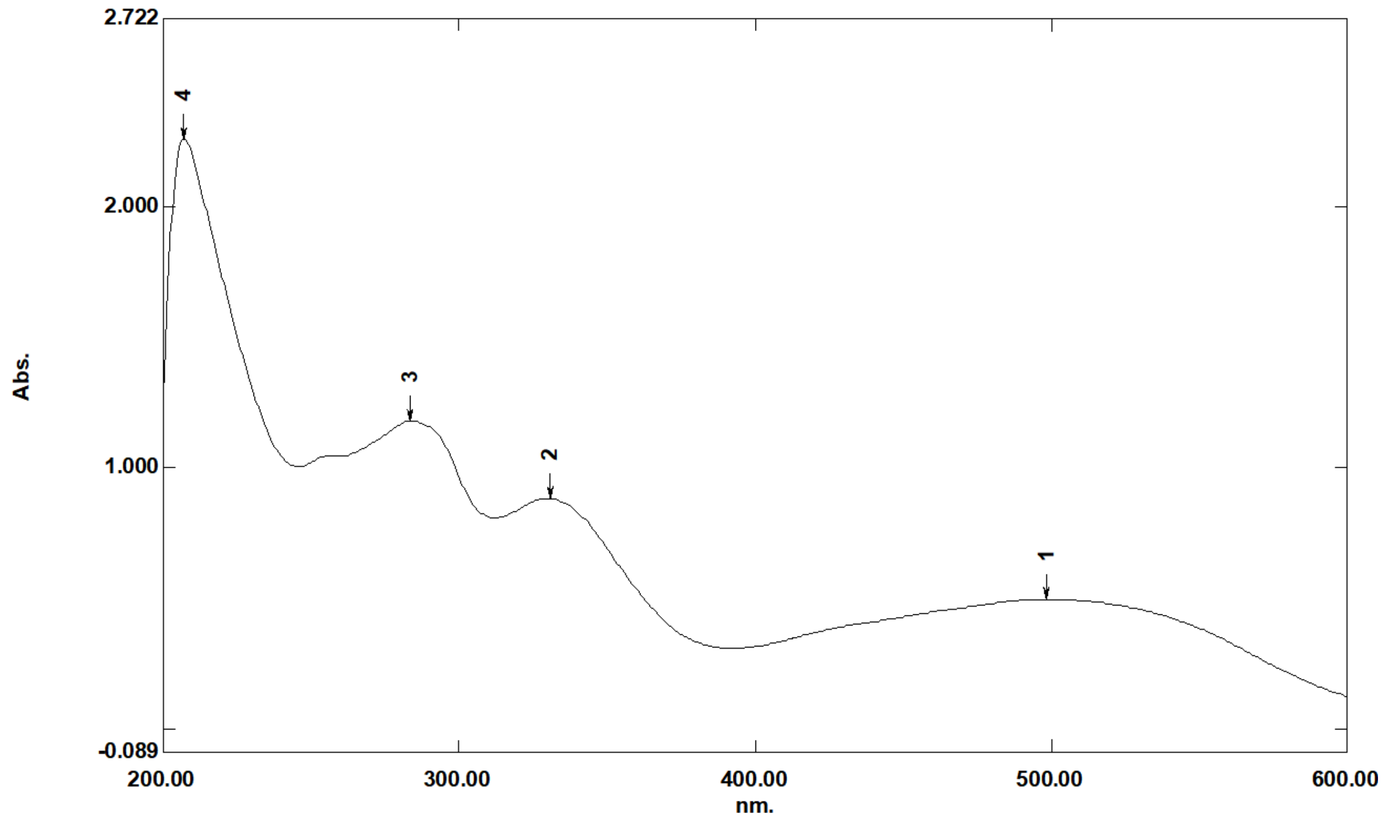

Figure S9. The spectroscopic data of NEN I (**5**).

(J) The IR spectrum of **5**

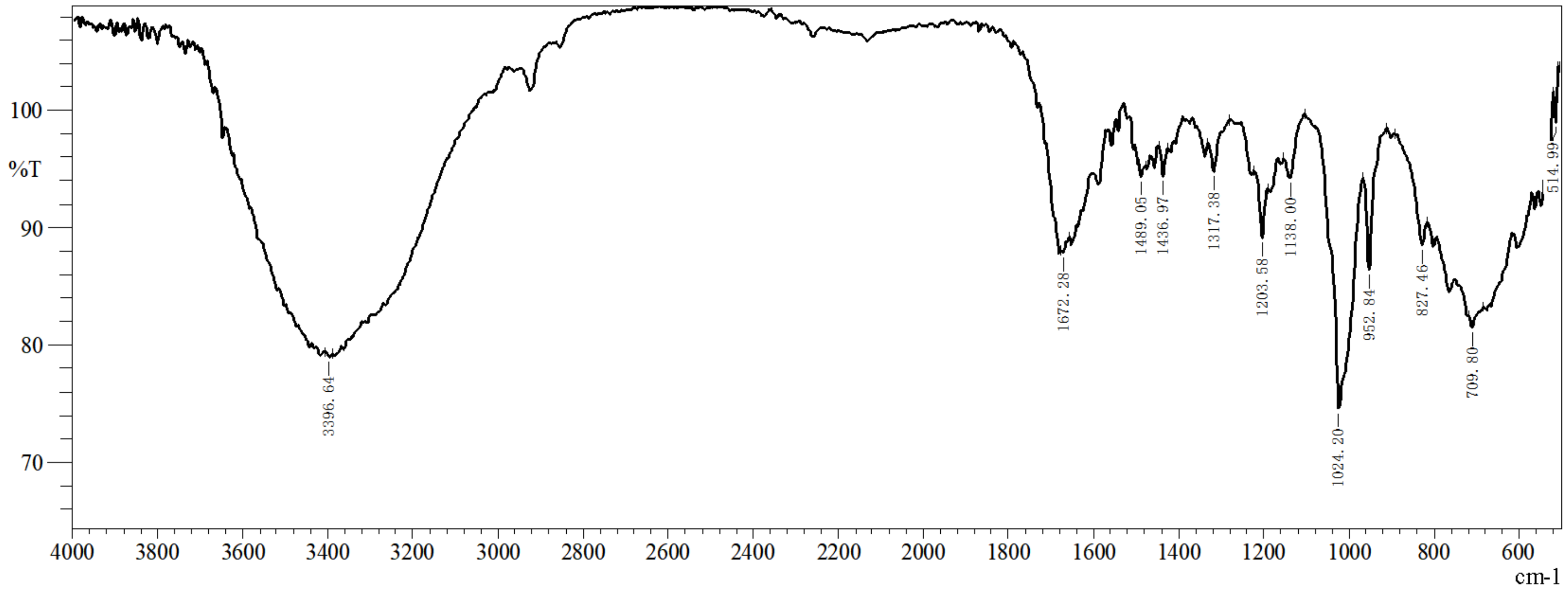

Figure S10. The spectroscopic data of homo-dehydrorabelomycin E (**6**).

(A) The HRESIMS spectrum of **6**

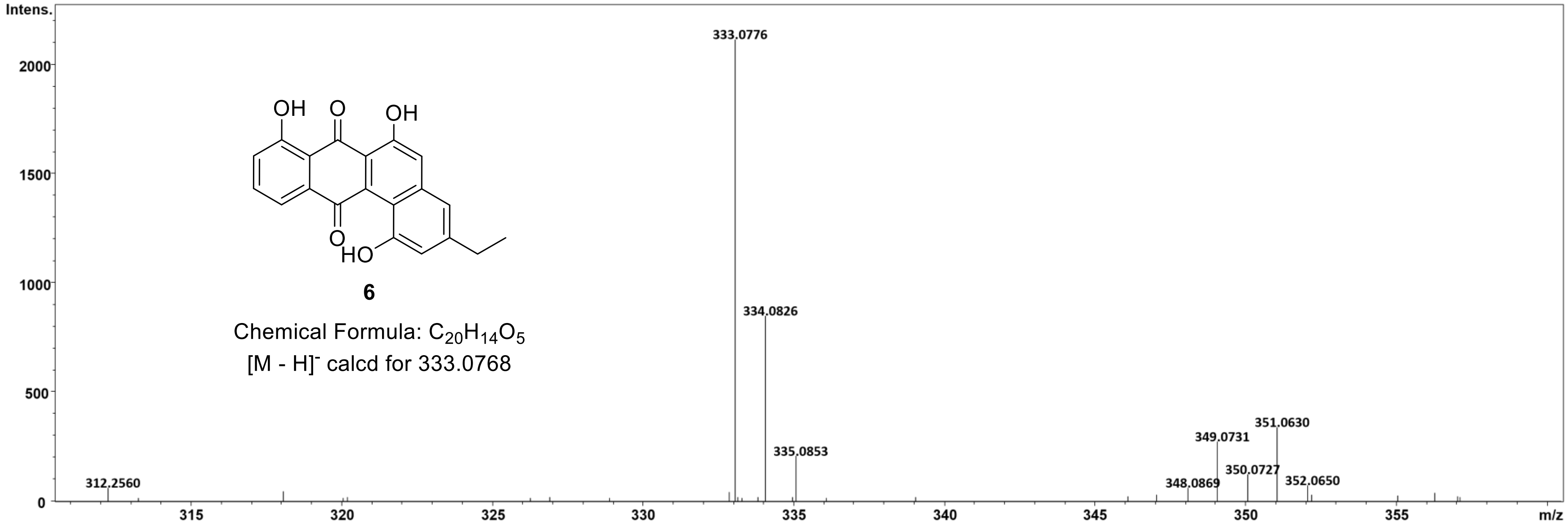

Figure S10. The spectroscopic data of homo-dehydrorabelomycin E (**6**).

(B) The ^{1}H-NMR spectrum of **6** (700 MHz for ^{1}H NMR in DMSO-d_6)

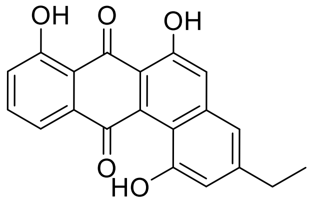

6

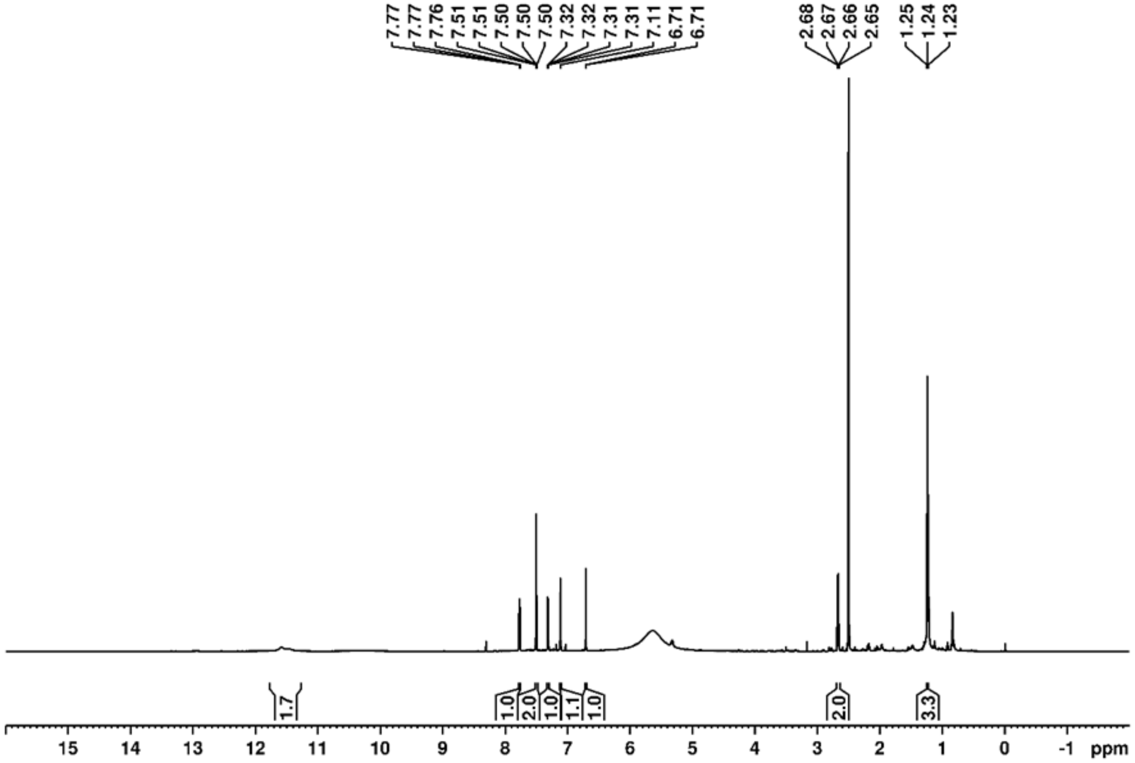

Figure S10. The spectroscopic data of homo-dehydrorabelomycin E (**6**).

(C) The UV spectrum of **6**

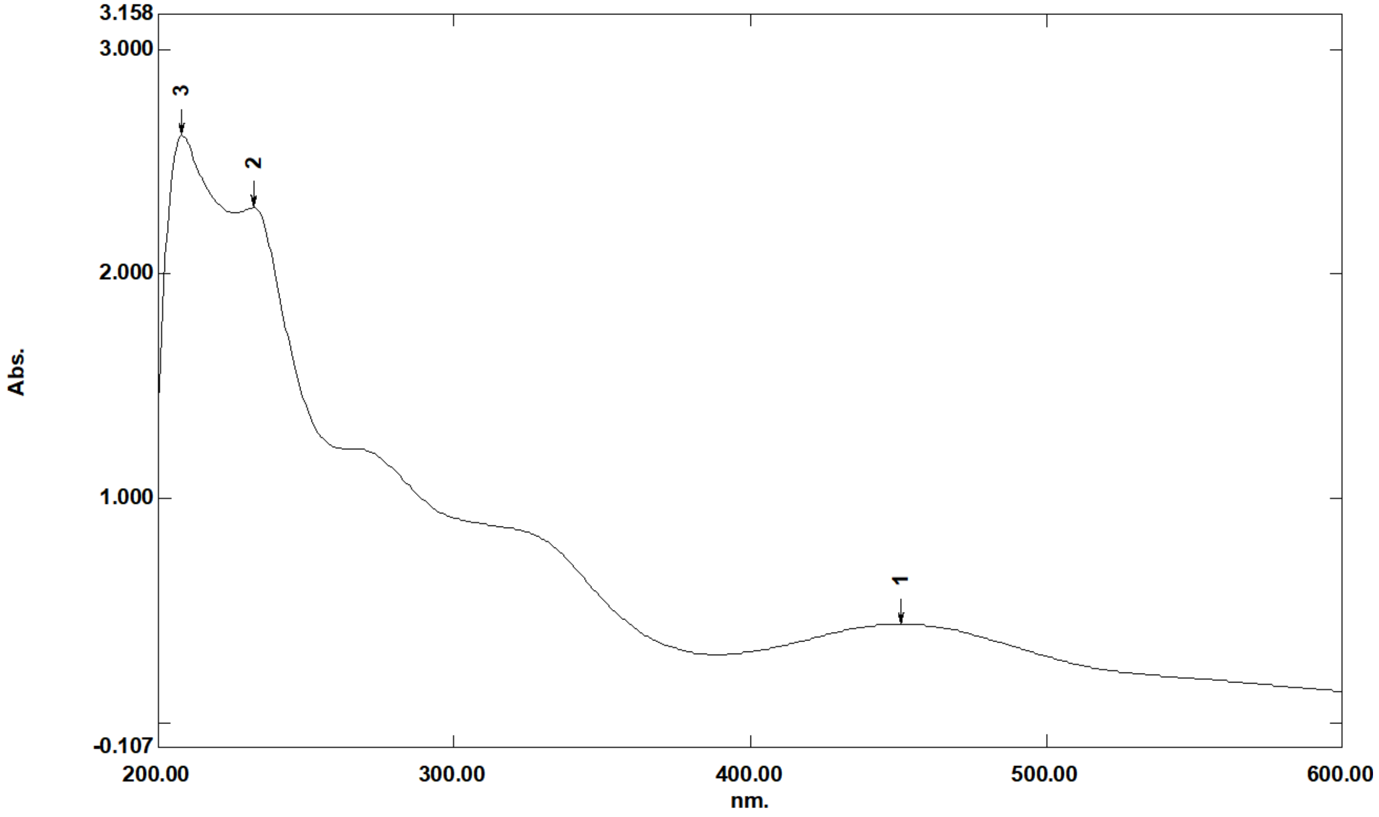

Figure S10. The spectroscopic data of homo-dehydrorabelomycin E (**6**).

(D) The IR spectrum of **6**

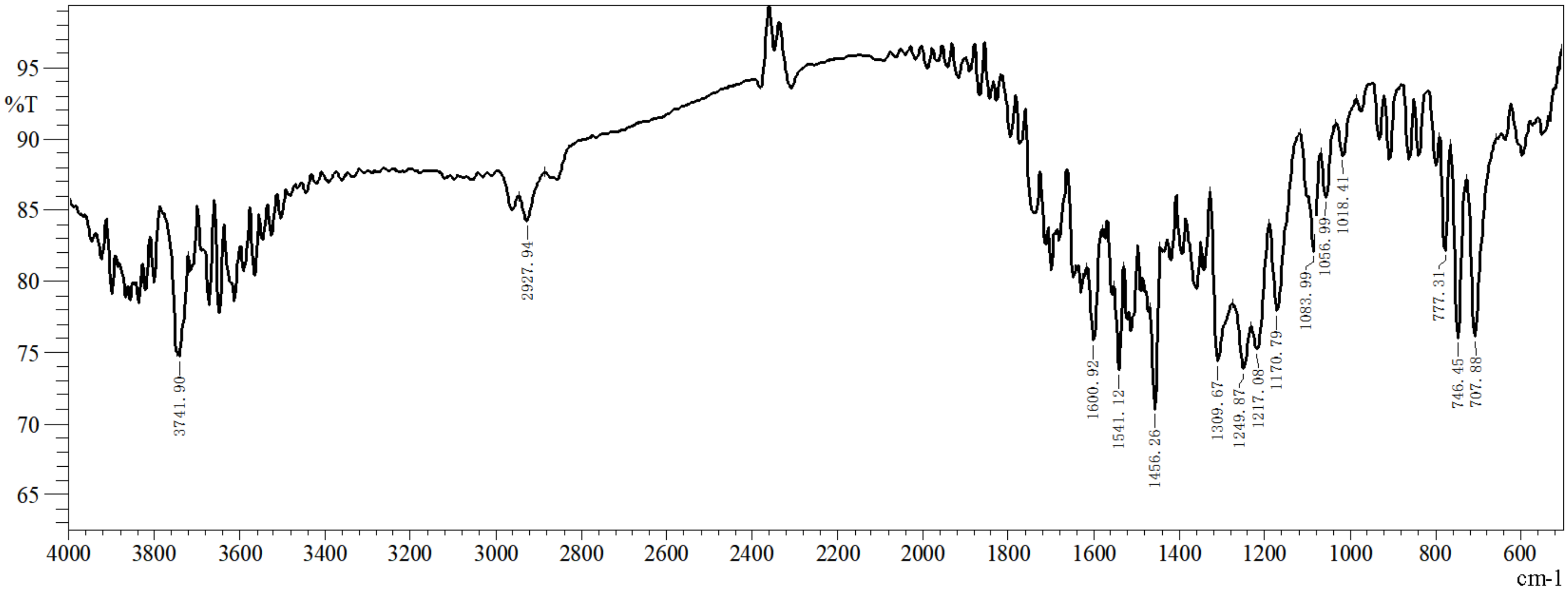

Figure S11. Sequence alignment of Nes5, Lom6, FlsH and Alp1U.

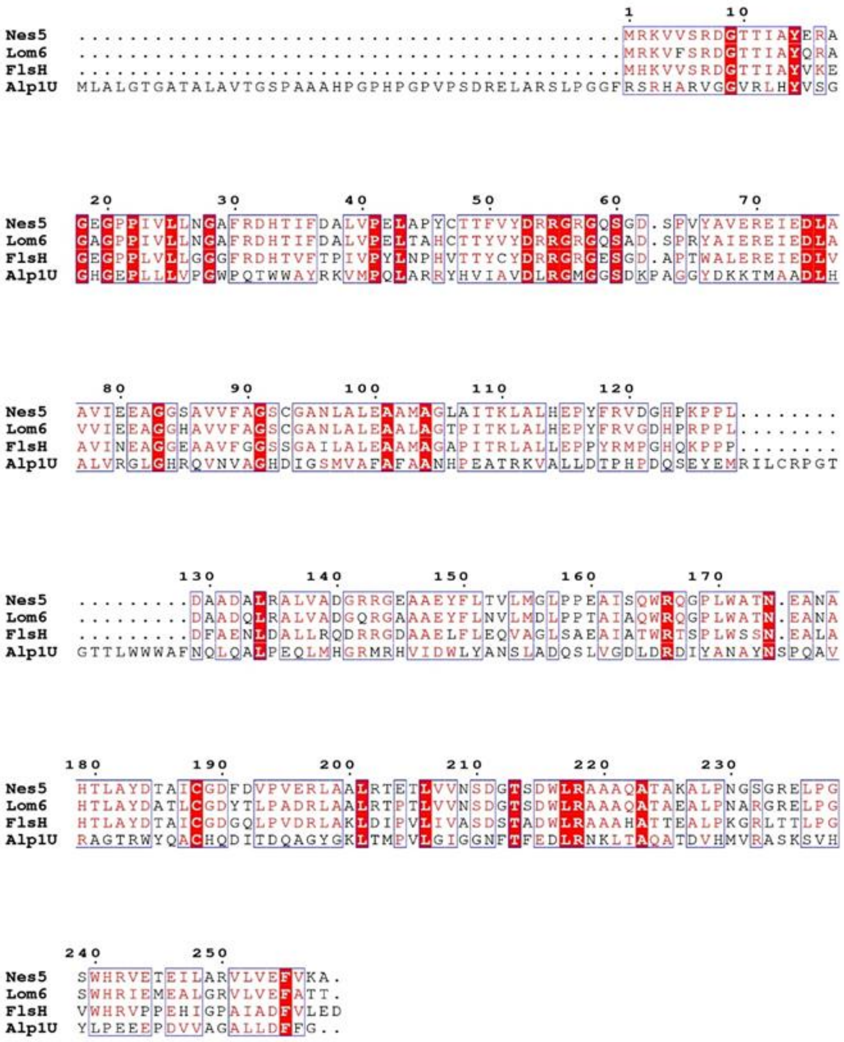

Sequences (Nes5:Lom6) Aligned. Score: 82.9457
Sequences (Nes5:FlsH) Aligned. Score: 62.7907
Sequences (Nes5:Alp1U) Aligned. Score: 13.5659
Sequences (Lom6:FlsH) Aligned. Score: 59.6899
Sequences (Lom6:Alp1U) Aligned. Score: 13.1783
Sequences (FlsH:Alp1U) Aligned. Score: 16.2162

Figure S12. The proposed biosynthetic pathway of **3**.

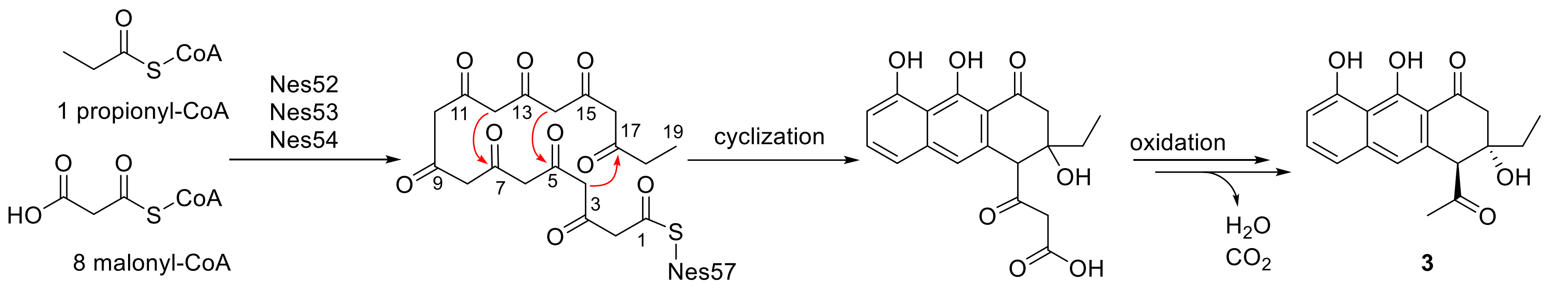

Supplement: Supplementary file 1 [file marinedrugs-24-00103-s001.zip › marinedrugs-4162483-supplementary.pdf]
